# Phytochemicals, Antioxidant Activities, and Toxicological Screening of Native Australian Fruits Using Zebrafish Embryonic Model

**DOI:** 10.3390/foods11244038

**Published:** 2022-12-14

**Authors:** Akhtar Ali, Sarah M. Kiloni, Paolin R. Cáceres-Vélez, Patricia R. Jusuf, Jeremy J. Cottrell, Frank R. Dunshea

**Affiliations:** 1 School of Agriculture and Food, The University of Melbourne, Parkville, VIC 3010, Australia; 2 School of Biosciences, The University of Melbourne, Parkville, VIC 3010, Australia; 3 Faculty of Biological Sciences, The University of Leeds, Leeds LS2 9JT, UK

**Keywords:** kakadu plum, davidson plum, quandong peach, muntries, molecular docking, bioactive compounds, flavonoids, safety evaluations

## Abstract

Phytochemicals play a pivotal role in human health and drug discovery. The safety evaluation of plant extracts is a prerequisite to ensure that all phytochemicals are safe before translational development and human exposure. As phytochemicals are natural, they are generally considered safe, although this is not always true. The objective of this study was to investigate and compare the phytochemical composition, antioxidant potential, and safety evaluation of native Australian Muntries (*Kunzea pomifera*), Kakadu plum (*Terminalia ferdinandiana*), Davidson plum (*Davidsonia*) and Quandong peach (*Santalum acuminatum*) through the in vivo vertebrate zebrafish embryonic model. The highest total phenolic content (TPC; 793.89 ± 22.27 μg GAE/mg) was quantified in Kakadu plum, while the lowest TPC (614.44 ± 31.80 μg GAE/mg) was quantified in Muntries. Developmental alterations, mortality, and morbidity were assessed for toxicological screening of these selected native Australian fruit extracts. In this study, muntries were quantified as having the least LC_50_ value (169 mg/L) compared to Davidson plum (376 mg/L), Kakadu plum (>480 mg/L), and Quandong peach (>480 mg/L), which indicates that muntries extract was more toxic than other fruit extracts. Importantly, we found that adverse effects were not correlated to the total phenolic content and antioxidant potential of these native Australian fruits and cannot simply be predicted from the in vitro analysis. Conclusively, these selected native Australian fruit extracts are categorized as safe. This study could explore the use of these native Australian fruits in cosmetics, pharmaceuticals, and drug discovery.

## 1. Introduction

Plant bioactive constituents are a vital component of traditional medicine used to treat various ailments [1]. In the past few decades, plants or plant-derived natural products have become the leading source of drug discovery [2]. Natural products from plants, marine organisms, or microorganisms have several nutritional and therapeutic properties. Many drugs worldwide are obtained from plants and classed as plant derivatives due to their therapeutic value [3]. Fruits, vegetables, herbs, spices, and other medicinal plants are widely used in natural and conventional medicine due to the increasing recognition of their health properties [4]. Although thousands of synthetic drugs have been introduced into the market for primary health care; plants or plant-derived bioactive compounds are still in use as potent alternative options. The assumption that natural medicines from plants are safe and free from adverse effects is misleading unless and until their properties are thoroughly investigated and assessed through different cell or in vivo models [4]. Phenolic metabolites can also exhibit potential adverse effects; they can act as endocrine disruptors, disturb iron absorption, interact with drugs, and be carcinogenic/genotoxic [5]. Alkaloids, lectins, phorbol esters, essential oils, and phenolic compounds have proven responsible for adverse effects when consumed in higher amounts. The extent of the toxic effects depends on the plant material, species, amount consumed, and susceptibility of the target. Some studies reported the presence of carcinogenic, mutagenic, and genotoxic substances in plant-derived traditional medicines [4]. Therefore, the potential toxic effect of natural products should be evaluated to ensure (adequate) protection of humans who consume these plants or their products.

Furthermore, toxicological studies may provide knowledge regarding the potential risks associated with using bioactive compounds. It is estimated that about 25 % of drug-associated problems are due to drug toxicity. Thus, evaluating potential toxicity levels remains a significant concern in clinical medicine and drug discovery, as quantitative data on toxicological studies is limited.

The toxicological screening of plant extracts through zebrafish has some advantages because they have rapid embryonic development, are transparent, and do not require invasive exposure techniques [6]. Embryonic zebrafish are a cheap, reliable, and convenient animal model for statistically dose-dependent toxicological studies [6]. This model can be used for high-throughput screening of compounds/drugs due to rapid development, less dose requirement of compounds/drugs, advantages of dissolving compounds/drugs directly in the same medium where they grow/develop, and high fecundity [7,8]. In recent times, this model has emerged in the field of pharmacology and toxicology due to its quickly reproducible results [9]. The zebrafish model has been established as an ideal complementary model breaching the gap between cell-based in vitro studies and other in vivo animal models. The genome of the zebrafish shares 70% homology with the human genome, while about 84% of zebrafish genes are associated with human pathologies [5,8,10]. The main objective of this study was to investigate the phytochemical composition, antioxidant activities and toxicological screening of native Australian fruits using zebrafish embryos. This study could explore the use of selected fruits in pharmaceuticals and drug discovery. 

## 2. Materials and Methods

### 2.1. Chemical and Reagents

Analytical-grade reagents were used in this experiment [11,12]. Phenolic contents and antioxidant activities of fruit extracts were measured using; Folin–Ciocalteu (F-C) phenol reagent, sodium carbonate, gallic acid, aluminum chloride, sodium acetate, quercetin, DPPH, ABTS, potassium persulfate, ferrous chloride, ferrozine, ethylenediaminetetraacetic acid (EDTA), iron (II) heptahydrate, *L*-ascorbic acid, and 3-hydroxybenzoic acid were purchased from Sigma Aldrich (St. Louis, MO, United States) while 30% hydrogen peroxide (H_2_O_2_) and 98% sulfuric acid (H_2_SO_4_) were purchased from Chem-Supply Pty Ltd. (Adelaide, SA, Australia) and RCI Labscan Ltd. (Bangkok, Thailand). Pure phenolic standards and LC-MS grade formic acid were also purchased from Sigma Aldrich (St. Louis, MI, USA). HPLC vials were purchased from Agilent Technologies (Santa Clara, CA, USA) while 96 well plates were purchased from Thermo Fisher Scientific Inc. (Scoresby, VIC, Australia). 

### 2.2. Preparation of Plant Extracts

Native Australian fruits in freeze-dried form were purchased from the Australian Super Food Co (https://austsuperfoods.com.au, accessed on 21 September 2021) (www.austsuperfoods.com.au, accessed on 21 September 2021). The method of Ali et al. [13], with some modifications, was used to extract bioactive compounds from selected fruits. Phenolics were extracted from the selected fruits using a 2/30 (*w/v*) sample-to-solvent ratio with 80% analytical-grade methanol in hexaplicates. The extraction process was completed in an orbital shaker (ZWYR-240 incubator shaker, Labwit Scientific, Ashwood, VIC, Australia) for 16 h at 150 rpm and 4 °C followed by centrifugation (Allegra X-12R centrifuge, Brea, CA, USA) at 8000 rpm for 20 min. All supernatants were pooled, concentrated using a rotary evaporator (Heidolph, Schwabach, Germany) and freeze-dried for at least 72 h, and stored at −80 °C for further analysis. 

### 2.3. Measurement of Total Phenolic Content (TPC) and Total Flavonoid Content (TFC)

The TPC and TFC were determined by following the methods of Ali et al. [12]. Briefly, 25 μL of phenolic extract mixed with 25 μL 25 F-C reagent (25% in H_2_O) and 200 μL of milli-Q water before 5 min incubation at room temperature in the dark. After that, 25 μL of sodium carbonate in water (%) was added and incubated for 1 h at room temperature. Gallic acid (0–200 μg/mL) in analytical grade ethanol was used to generate the standard equation at 765 nm using a spectrophotometer. For TFC, the Sharifi-Rad et al. [14] method was used with minor modifications. The phenolic extract (80 μL) was mixed with 80 μL of 2% aluminum chloride and 120 μL of 0.6 M sodium acetate in water before incubation for 2.5 h at room temperature in the dark. The absorbance was measured at 440 nm, and quercetin (0–50 μg/mL) in analytical-grade methanol was used to generate the standard curve. 

### 2.4. Measurement of Antioxidant Activities

Antioxidant activities including 2,2′-azinobis-(3-ethylbenzothiazoline-6-sulfonic acid (ABTS), hydroxyl-radical scavenging activity (•OH-RSA), ferrous ion chelating assay (FICA) and 2,2′-diphenyl-1-picrylhydrazyl (DPPH) free-radical-scavenging activities were measured in this experiment. The DPPH assay was conducted by following the method of Chou et al. [15] with modifications. To do this, 25 μL of the phenolic extract was mixed with 275 μL 0.1 mM methanolic DPPH solution incubated in the dark at room temperature for 30 min, and absorbance was measured at 517 nm. The method of Zahid et al. [16] with some changes was used for the ABTS assay, while the •OH-RSA and FICA activities were tested by using the methods of Bashmil et al. [17] and Ali et al. [12]. 

### 2.5. LC-MS Analysis

Polyphenolic compounds were identified by using an Agilent 6520 Accurate-Mass quadrupole time of flight (QTOF) machine described by Suleria et al. [18] after modifications. Column, gradient, chromatographic, and other machine conditions were the same as we reported [11,19]. MassHunter Workstation Software (version B.06.00) (Agilent, Santa Clara, CA, USA) was used to extract and identify phytochemicals. The mass spectra of twenty-four external standards were also obtained and standard equations were generated as described by Ali et al. [20]. 

### 2.6. In Vivo Acute Toxicity Tests of Australian Native Fruits

The in vivo acute toxicity tests of Australian native fruit extracts (6 different concentrations at 15, 30, 60, 120, 240, and 480 mg/L with E3 medium (0 mg/L) as a control) were analyzed by following the method of Cáceres-Vélez et al. [2]. Zebrafish (AB wild type) were maintained at the Danio rerio facility at the University of Melbourne, Victoria, Australia. Briefly, animals were set up in group spawning tanks (5 pairs), and eggs were collected into E3 medium (5 mM NaCl; 0.17 mM KCl; 0.33 mM CaCl_2_; 0.33 mM MgSO_4_). All experiments were performed in triplicates (from the different tanks). At 3 to 4 h postfertilization, embryos were pre-exposed to the test solution for 1–2 h. For the toxicological screening, individual zebrafish were exposed in individual wells (500 μL) of 48-well plates (for each concentration: 4 internal controls and 20 treatment wells). Zebrafish toxicity assessments were performed every 24 h until 96 h postfertilization. 

### 2.7. Virtual Toxicological Screening and Molecular Docking of Selected Phytochemicals 

To predict the toxicity of individual phytochemical metabolites, we used the pkCSM platform (biosig.lab.uq.edu.au/pkcsm, accessed on 21 July 2022). Using computational methods to test the potential drug metabolites helps to reduce the number of experimental studies and improve the success rate in pharmacokinetics studies. To investigate toxicological properties; maximum tolerated dose (human), AMES toxicity, hERG I, and II inhibitors, skin sensitization, hepatotoxicity, *Tetrahymena pyriformis* toxicity, oral rat acute toxicity (LD_50_), minnow toxicity and oral rat chronic toxicity of most abundant phytochemical metabolites were virtually predicted. In silico docking was also conducted to predict the hepatoprotective potential of selected phytochemical metabolites in native Australian fruits, as described by Ali et al. [11]. Grid box dimensions were x = −34.87, y = 54.62, y = 12.91 while docking ligands with a length within 20 Å.

### 2.8. Statistical Analysis

One-way ANOVA analysis was performed, and a pairwise comparison analysis was used to compare treatments. The non-linear logarithmic (log10) regression function was used to obtain the LC_50_ value for each extract. All data analysis was performed using Graph pad prism (Dotmatics, Boston, MA, USA) statistical software version 9.4.1.

## 3. Results and Discussion

### 3.1. TPC, TFC, and Antioxidant Activities of Native Australian Fruits

**Figure 1:**
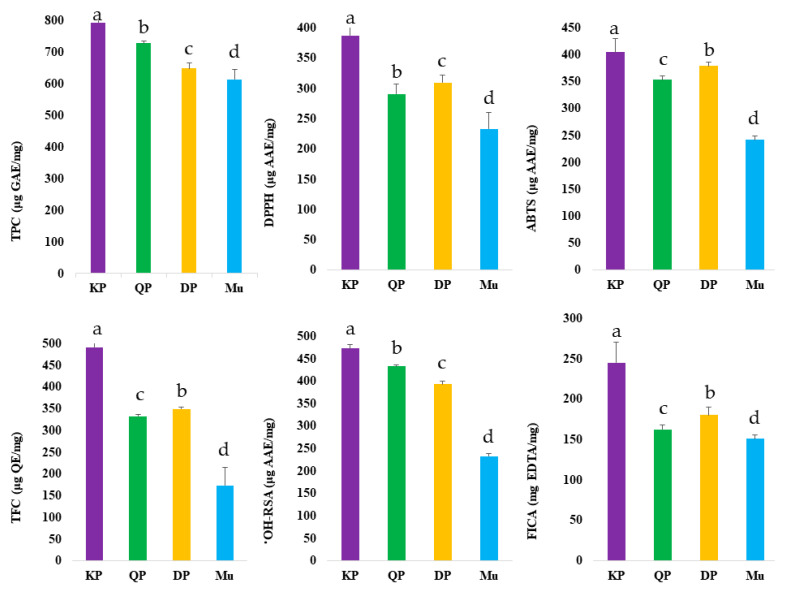
Quantification of phenolic contents and antioxidant activities of native Australian Muntries (Mu), Kakadu plum (KP), Davidson plum (DP), and Quandong peach (QP). Values with the letters (a–d) are significantly different from each other (*p* < 0.05).

Fruits are a rich source of various bioactive compounds, including phenolic and non-phenolic compounds. In this study, we quantified TPC, TFC, and the antioxidant potential of native Australian Muntries, Quandong peach, Kakadu plum, and Davidson plum. The results of TPC, TFC, and antioxidant activities are presented in Figure 1 and Table S1. 

The highest TPC (793.89 ± 22.27 μg GAE/mg) was measured in Kakadu plum while the lowest TPC (614.44 ± 31.80 μg GAE/mg) was quantified in Muntries. The TPC of Davidson plum (649.83 ± 15.75 μg GAE/mg) and Quandong peach (728.53 ± 6.57 μg GAE/mg) was also quantified. Previously, Konczak et al. [21] and Tan et al. [22] also measured the highest TPC value of Kakadu plum, while [23] Sommano et al. [23] measured the highest TPC (893.1 μg GAE/100 g) of Davidson plum compared to other native Australian plants. Previously, Chuen et al. [24] measured the TPC in Davidson plum in the range of 35.17 to 94.13 μg GAE/g depending on the solvent used. Kakadu plum and Davidson plum were measured with the highest TFC (491.21 ± 20.24 μg QE/mg and 349.45 ± 4.12 μg QE/mg), respectively while the Muntries contained the lowest TFC value (173.17 ± 41.42 μg QE/mg). Previously, Sakulnarmrat et al. [25] also reported a higher TFC in Davidson plum compared to quandong peach, while Chuen et al. [24] reported TFC of Davidson plum in the range of 22.33 to 78.33 mg GAE/g in different solvents. Native Australian fruits have higher phenolic contents, especially anthocyanins, compared to Australian-grown blueberries [11,21,26], a fruit generally thought of as having high TPC. Kakadu plum has been identified as a ‘medicinal plum’ due to its high concentration of phenolic contents Sakulnarmrat et al. [26].

Fruits and vegetables are widely used as a source of antioxidants to scavenge the free radicals in the human body. Generally, polyphenols are considered vital bioactive constituents that have many health benefits Ali et al. [13]. They act as hydrogen atom donors, metal chelators, reducing agents, and free radical scavengers in the biological system. The antioxidant potential of native Australian fruits was quantified through in vitro antioxidant assays including ABTS, DPPH, OH-RSA, and FICA (Figure 1, Table S1). The highest ABTS (404.74 ± 24.61 μg AAE/mg), DPPH (387.31 ± 20.49 μg AAE/mg), •OH-RSA (472.47 ± 9.45 μg AAE/mg) and FICA (244.82 ± 25.97 μg EDTA/mg) were found in Kakadu plum while the lowest antioxidant potential was quantified in muntries (Figure 1). Previously, the higher DPPH of Kakadu plum was also reported by Tan et al. [22] and Sommano et al. [23], while Sommano et al. [23] also reported higher ABTS of the Kakadu plum compared to other native fruits selected in this study. 

Native Australian fruits have a higher antioxidant potential compared to blueberries, which are known for having a high antioxidant potential [11]. The antioxidant activity of phenolic compounds may depend upon the method used to extract the bioactive compounds. Previously, a few studies were conducted to measure the antioxidant potential of Australian native fruits. However, in-depth screening is limited due to bioactive compounds’ complex nature and commercial standards’ unavailability. Therefore, the LC-ESI-QTOF-MS is a reliable tool to identify and characterize these fruits’ phenolic and non-phenolic compounds.

### 3.2. LC-MS Identification and Quantification/Semi-Quantification of Target Compounds

LC-ESI-QTOF-MS was employed for the identification and characterization of phytochemicals. A total of 427 phytochemical metabolites were tentatively identified (Table S2, Figure S1) in this experiment, while a total of 50 phenolic metabolites were quantified/semi-quantified by measuring the peak area (%) of the most abundant metabolites (Table S3). 

#### 3.2.1. Phenolic Acids

In this experiment, seventeen hydroxybenzoic acids, thirty-seven cinnamic acids, and ten other phenolic acids were putatively characterized, while fifteen phenolic acids were quantified in these fruits (Tables S2 and S3). The highest concentration of quinic acid (14.31%), cinnamic acid (12.84%), *p*-coumaric acid (10.85%), protocatechuic acid (8.94%), *p*-hydroxybenzoic acid (4.94%) and ellagic acid (2.5%) while sinapic acid and chlorogenic acid were measured in Kakadu plum in considerable amounts. Moreover, 3-sinapoylquinic (1.32%) acid was the only phenolic acid measured in Kakadu plum. The highest concentration of ellagic acid (6.18%) was measured in the Davidson plum, while the lowest concentration of ellagic acid (0.93%) was measured in the Kakadu plum. Muntries contain the highest concentration of protocatechuic acid (10.30%) and *p*-coumaric acid (8.28%), while the lowest concentration of syringic acid (0.90%) and chlorogenic acid (1.19%). Previously, ellagic acid (1.05%) was quantified in the methanolic extract of Davidson plum by Cheesman et al. [27]. Previously, ferulic acid (18.07 ± 3.41 μg/g) was measured in Davidson plum while a 12.17 ± 3.11 ug/g in Kakadu plum. Chlorogenic acid was measured in Muntries (142.18 ± 14.01 μg/g) and Quandong peaches (80.98 ± 6.91 μg/g), respectively, by Ali et al. [11]. Previously, Konczak et al. [28] also identified chlorogenic acid in Quandong peaches. 

#### 3.2.2. Anthocyanins

We tentatively identified a total of 61 anthocyanins, while fifteen anthocyanins were semi-quantified in native Australian Muntries, Kakadu plum, Davidson plum, and Quandong peach (Tables S2 and S3). Davidson plum contained the highest concentration of total anthocyanins (54.0%). Delphinidin 3-*O*-sambubioside (20.50%), peonidin 3-sambubioside (5.68%), cyanidin 3-*O*-galactoside (5.28%), petunidin 3-sambubioside (5.23%), delphinidin 3-*O*-glucoside (4.16%), cyanidin 3-glucoside (3.06%) and cyanidin 3-*O*-rutinoside (2.79%) were measured with the highest concentration in Davidson plum (Table S3). Muntries and quandong peaches were also quantified at 10.10% and 14.02% of total anthocyanins. Previously, delphinidin 3-sambubioside (0.16 ± 0.04 mg/g) and cyanidin 3-sambubioside (0.02 ± 0.001 mg/g) were quantified in Davidson plum. Cyanidin 3-glucoside (0.13 ± 0.005 mg/g) was measured in quandong peach by Konczak et al. [28], while delphinidin 3-glucoside (0.3 ± 0.01 μg/g) and cyanidin 3-glucoside (0.8 ± 0.05 μg/g) were measured in Muntries [22,29]. 

#### 3.2.3. Non-Anthocyanin Flavonoids

In this experiment, 18 non-anthocyanins were measured while 177 compounds were putatively identified as non-anthocyanin flavonoids, reported in Tables S2 and S3. Procyanidin B2 (9.04%), taxifolin (1.50%), and diosmetin (1.50%) were only measured in Muntries, while 3’-*O*-methylviolanone (4.55%), genistein (1.32%), phloretin (1.48%) and quercetin-3-glucoside (1.89%) were measured in Kakadu plum. Moreover, naringenin (3.63%) was only measured in Davidson plum. The highest concentration of epicatechin (10.73%) was measured in Muntries while the lowest concentration of epicatechin (4.62%) was measured in Kakadu plum. Kaempferol was quantified in Muntries (0.66%), quandong peach (0.91%), and Kakadu plum (2.37%), respectively. Previously, rutin (0.53 ± 0.01 mg/g) and kaempferol (0.61 ± 0.01 mg/g) were measured in Quandong peach reported by Konczak et al. [28] while Kakadu plum already identified with quercetin, luteolin, kaempferol glucosides, and hesperetin luteolin reported by Mani et al. [30]. 

#### 3.2.4. Other Polyphenols

Sixty-one other phenolic metabolites, including two alkylphenols, twelve coumarins and derivatives, three phenolic terpenes, nine tyrosols and derivatives, ten xanthones, and twenty-five other polyphenols were putatively identified. Coumarin and pyrogallol were quantified with external standards (Tables S2 and S3). The highest concentration of coumarin was quantified (6.02%) in Muntries, while the lowest concentration of coumarin (3.96%) was quantified in Kakadu plum. Kakadu plums and Muntries contain higher concentrations of pyrogallol than Quandong peaches. A total of 36 stilbenes and lignans were tentatively identified in selected fruits. Most of the compounds of stilbenes and lignans were only identified in the Kakadu plum (Table S2).

#### 3.2.5. Non-Phenolic Compounds

A total of 17 non-phenolic metabolites (terpenoids, sesquiterpenoids, and alkaloids) were tentatively identified in selected fruits (Table S2). Compounds 414 (esculentic acid), 415 (limocitrin), 416 (albiflorin), and 420 (longifolene) were only identified in muntries while isoobacunoic acid 17-β-D-glucoside (compound 412) was only identified in quandong peach. A total of six alkaloids were also identified in muntries. 

#### 3.2.6. Heatmap Hierarchical Clustering 

**Figure 2:**
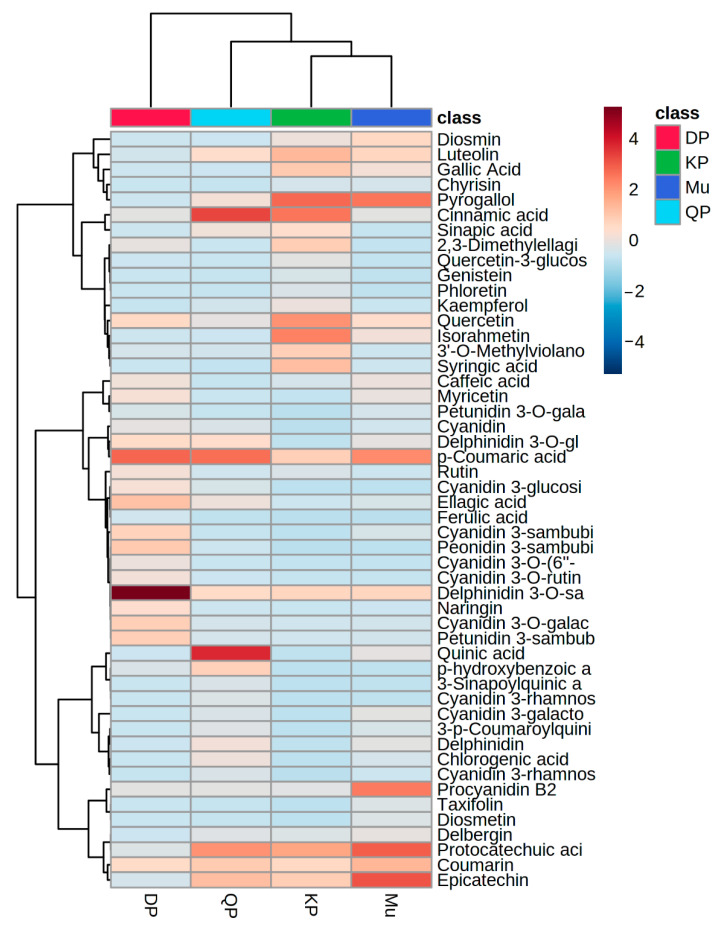
Heatmap hierarchical clustering of quantified phenolic compounds in Davidson plum (DP), Quandong peach (QP), Kakadu plum (KP), and Muntries (Mu).

The quantified data was used to generate heatmap clustering by using MetaboAnalyst (https://www.metaboanalyst.ca, accessed on 2 September 2022), while a total of nine row-wise and two column-wise clusters were generated and highlighted with hierarchical clustering given in Figure 2.

The heatmap shows *p*-coumaric acid, delphinidin 3-*O*-sambubioside, peonidin 3-sambubioside, petunidin 3-sambubioside, ellagic acid, and cyanidin 3-galactoside have a higher concentration in Davidson plum while quinic acid, cinnamic acid, protocatechuic acid, delphinidin 3-*O*-sambubioside were measured higher concentration in quandong peach. Muntries contain a higher concentration of epicatechin, protocatechuic acid, procyanidin B2, *p*-coumaric acid, and pyrogallol, while Kakadu plum and Muntries were made a cluster with each other. Cinnamic acid, pyrogallol, quercetin, isorhamnetin, and epicatechin showed a higher concentration in the Kakadu plum. 

### 3.3. Distribution of Phytochemical Metabolites in Native Australian Fruits

**Figure 3:**
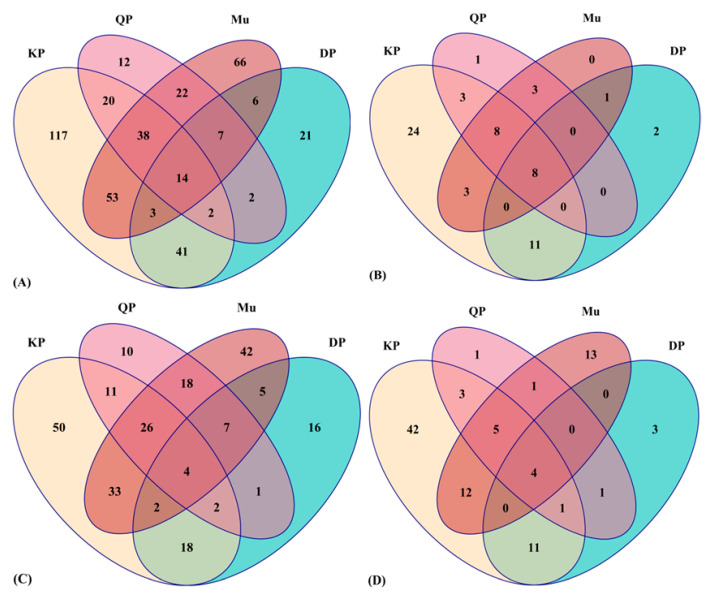
Venn diagram distribution of the total number of phytochemicals (**A**), the total number of phenolic acids (**B**), the total number of flavonoids (**C**), and the total number of other polyphenols (**D**) in Muntries (Mu), Kakadu plum (KP), Davidson plum (DP) and Quandong peach (QP).

A total of 289, 96, 119, and 210 phytochemical metabolites were tentatively identified in Muntries, Kakadu plum, Davidson plum, and Quandong peach, respectively (Table S2). Venn diagram analysis is a powerful way to present many phytochemicals in a single set. The distribution of the total number of phytochemicals (A), the total number of phenolic acids (B), the total number of flavonoids (C), and the total number of other polyphenols (D) in selected Muntries, Kakadu plum, Davidson plum, and Quandong peach, respectively are shown in the Venn diagram in Figure 3.

Figure 3A describes the total number of phytochemical metabolites distributed in the four selected fruits. A total of 12, 21, 66, and 117 unique phytochemical metabolites were putatively identified in the Quandong peach, Davidson plum, Muntries, and Kakadu plum. A total of 14 phytochemical metabolites overlapped in all four fruits, while a total of 20 phytochemicals overlapped in the Quandong peach and Kakadu plum. A total of 41 phytochemicals overlapped in the Kakadu plum and Davidson plum, while a total of 53 phytochemicals overlapped in the Kakadu plum and Muntries (Figure 3A). Figure 3B illustrates that 24 unique phenolic acids were identified in the Kakadu plum, while the Quandong peach and Davidson plum have only one and two unique phenolic acids, respectively. A total of 8 phenolic acids overlapped in all four fruits. Figure 3C depicts the total number of flavonoids in selected Australian native fruits. It indicates that a total of 50 unique flavonoids were identified in the Kakadu plum, while a total of 42 unique flavonoids were identified in Muntries. Likewise, 10 and 16 unique flavonoids were identified in the Quandong peach and Davidson plum.

Furthermore, a total of 33 flavonoids overlapped in Kakadu plum and Muntries, while a total of 18 flavonoids overlapped in Quandong peach and Muntries. Kakadu plum and Davidson plum also shared 18 common flavonoids. Only four flavonoids (rutin, isorhamnetin 4′-*O*-glucuronide, 6-hydroxyluteolin, and 3′,4′,7-trihydroxyisoflavanone) were overlapped in all four fruits. Figure 3D shows that a total of 42 unique other polyphenols were identified in the Kakadu plum, while 1, 3, and 13 unique other polyphenols were identified in the Quandong peach, Davidson plum, and Muntries, respectively. 

### 3.4. In Vivo Acute Toxicity of Native Australian Fruits

**Figure 4:**
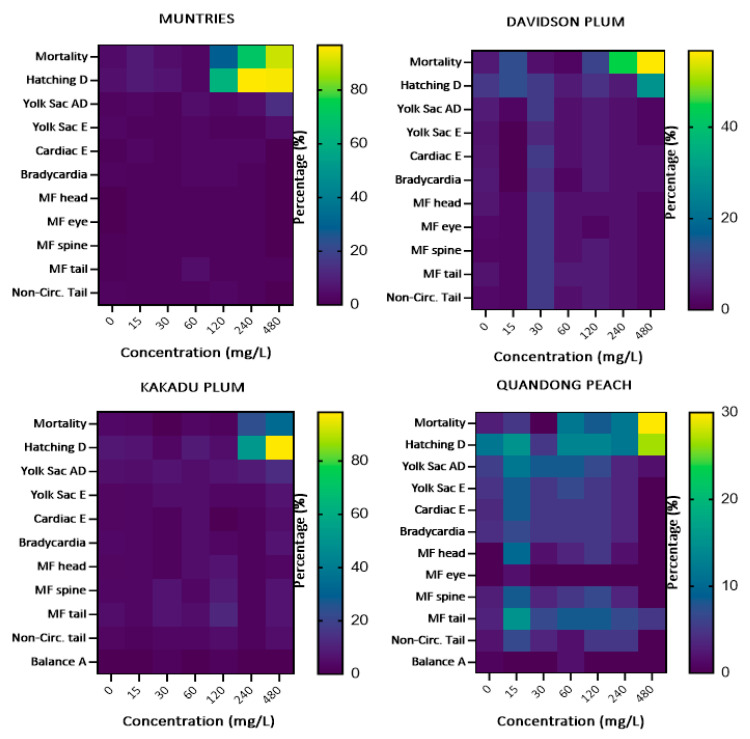
Heatmaps of the changes in zebrafish embryos during the exposure of Australian muntries, Davidson plum, Kakadu plum, and Davidson plum extracts at 96 h.

Developmental alterations (cardiac edema, yolk sac edema, delayed yolk sac absorption, and hatching delay), mortality (egg coagulation, dead embryos, and dead larvae), morbidity (bradycardia, balance alterations, and non-circulation in the tail) were assessed during the toxicological screening of Muntries, Kakadu plum, Davidson plum, and Quandong peach fruit extracts. To estimate the safe concentrations of these fruit extracts through in vivo model, toxicological tests were performed using the zebrafish embryonic–larval model. The total behavioral and adverse phenotypic effects after exposure to the different Australian native fruits for 96 h are shown in Figure 4. 

Significant variations were observed between the fruits. The mortality and hatching delay due to the Muntries extract were observed with a frequency of more than 80%. At the same time, the same frequency of hatching delay was also observed after exposure to the Kakadu plum extract for 96 h. The lowest mortality rate was observed in Quandong peach (30%), and Kakadu plum (33%) extracts during the exposure of 96 h.

#### 3.4.1. Mortality

**Figure 5:**
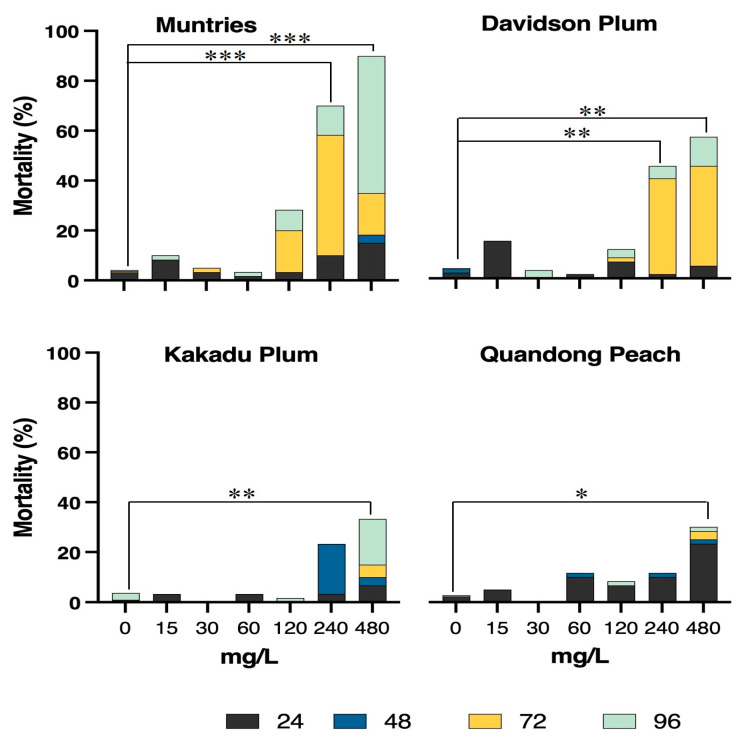
Percentage of mortality observed in zebrafish embryos exposed during 96 h to different concentrations of native Australian fruits. The colors represent the time at which mortality was observed. Significant differences are observed for muntries at 240 and 480 mg/L compared to controls (0), both values represented by *** *p* < 0.001. Davidson plum: ** *p* < 0.01 for 240 and 480 mg/L compared to controls (0). Kakadu plum: ** *p* < 0.01 for 480 mg/L compared to controls (0). Quandong peach: * *p* ≤ 0.05 at 480 mg/L compared to controls (0).

The untreated control groups (0 mg/L) showed low mortality (≤5.0% occurrence) and normal developmental morphology during the 96 h of extract exposure. The LC_50_ of each fruit extract was calculated by quantifying egg coagulation (from 0 to 24 h), dead embryos (24 to 48 h before hatching), and dead larvae after hatching (48 to 96 h), which were collectively referred to as mortality (Figure 5). 

For Kakadu plum and Quandong peach, the LC_50_-96h values were higher than the maximum concentration of each fruit extract tested while the lowest LC_50_ value was measured in muntries (169 mg/L) and Davidson plum (376 mg/L), respectively. Interestingly, the mortality rate was lower (≈10 to 15%) for the first 24 h due to the exposure to Muntries, Kakadu plum, and Davidson plum, while the mortality rate was higher during exposure to Quandong peach extracts. Critically, all fruit extracts at a concentration of 240 mg/L were observed to be safe for the first 48 h, while Muntries showed higher mortality, particularly after 96 h of exposure. Overall, Quandong peach, and Kakadu plum showed the lowest mortality rates of 30% after 96 h of exposure when compared to Davidson plum (less than 60%) and Muntries (≈90%), respectively. Muntries at the concentration of 240 mg/L and 480 mg/L have significantly higher mortality than other fruits (Figure 5). 

#### 3.4.2. Developmental Alterations and Morbidity

**Figure 6:**
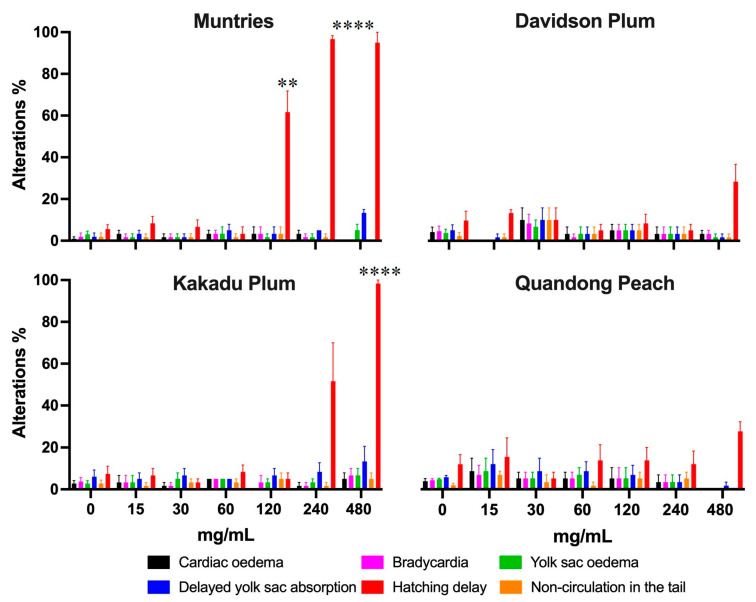
The graph shows the developmental alterations and morbidity in zebrafish embryos exposed for 96 h to different concentrations of native Australian fruits. Bars represent the mean ± SEM compared to the control group (*p* < 0.0001). Statistical significance represented by ** *p* ≤ 0.01, and **** *p* ≤ 0.0001.

Zebrafish embryos exposed to muntries over 120 mg/L significantly changed the hatching delay, while Kakadu plum also showed the same effect when exposed to the concentration of 480 mg/L (Figure 6). 

Davidson plum and quandong peach only showed up to a 30% increase in hatching delay at the highest concentration of 480 mg/L, suggesting that these two fruits are less toxic than either Muntries or Kakadu plum. When exposed to Muntries, zebrafish embryos exhibited a dose-dependent effect on hatching and yolk sac absorption delays. Almost all the tested fruit extracts are safe up to the concentration of 120 mg/L when zebrafish embryos/larvae are exposed for a 96 h period. There was no significant difference in any other alterations and malformations over the 96 h period, which indicates that these fruit extracts are safe over this period (Figure 6). To estimate the morbidity rate, occurrence of bradycardia, balance alteration, and non-circulation in the tail were quantified at 96 h which are reported in Figure 5. Overall, the morbidity rate was less than 10 % over the 96 h. Figure 6 shows that there are significant differences observed for embryos displaying hatching delays (Muntries) at 120, 240, and 480 mg/L compared to controls (0 mg/L) and for Kakadu plum (at 480 mg/L compared to control (0 mg/L) while Davidson plum and Quandong peach have no significant differences. 

#### 3.4.3. Malformations

**Figure 7:**
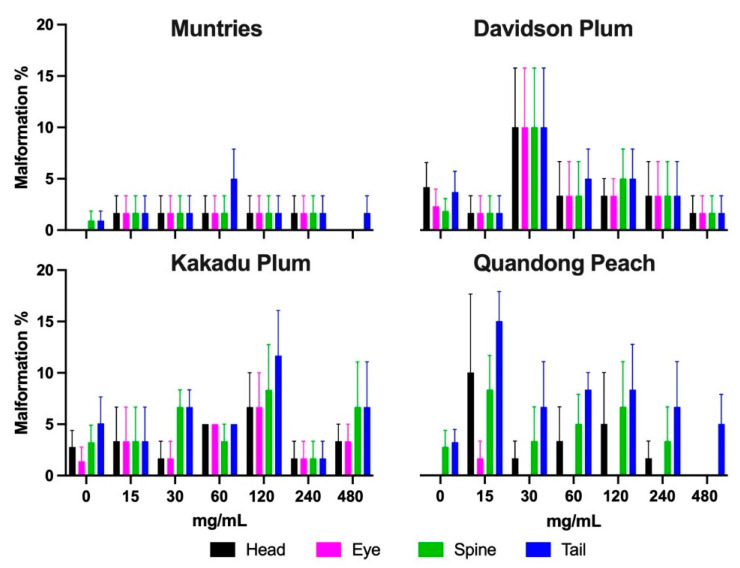
Malformations were observed in zebrafish exposed during 96 h post-fertilization to different concentrations of Kakadu plum, Davidson plum, quandong peach, and muntries. No statistically significant difference was observed, and the data represent the mean ± SEM.

Malformations (malformed head, eye, spine, and tail) were observed in zebrafish exposed for 96 h to different concentrations of Muntries, Kakadu plum, Davidson plum, and Quandong peach were quantified and are given in Figure 7. Overall, no significant changes were observed in malformations when these fruit extracts were exposed over 96 h.

#### 3.4.4. Extended Discussion 

For decades, natural plant bioactive metabolites have been used as therapeutic agents to improve human health. Several epidemiological studies revealed the beneficial effects of natural bioactive compounds, including alkaloids, polyphenols, terpenoids, or other phytochemicals, on cardiovascular, diabetes, obesity, neurological disorders, and cancers [31,32,33,34]. Australia has a wide range of native fruits and medicinal plants which have been used to treat different health disorders [35]. Kakadu plum, Davidson plum, quandong peach, and muntries have been used for several years in traditional medicine. Several studies have been reported on antioxidant and other biological activities. However, to the best of our knowledge, there have yet to be studies conducted to evaluate these fruit extracts’ safety through the in vivo developmental zebrafish model. This is the first study to comprehensively characterize different classes of phytochemicals in these native Australian fruits and directly correlate this to potential unwanted health effects. 

Toxicological screening is a prerequisite for approving natural bioactive compounds to be used as a food additive or in drug discovery in addition to their biological functions [36]. Previously, a few in vivo toxicological studies were conducted on extracts of native Australian fruits [37]. In the current study, muntries were found to have the lowest LC_50_ value (169 mg/L) compared to Davidson plum (376 mg/L), Kakadu plum (>480 mg/L), and quandong peach (>480 mg/L) which indicates that muntries extract was comparatively more toxic than other fruit extracts. The viability of zebrafish embryos was more than 97% at exposure concentrations of 60 mg/L in muntries, Kakadu plum, and Davidson plum at 96 h. The viability of zebrafish embryos declined at 240 mg/L and 480 mg/L of fruit extracts, respectively, consistent with increasing toxicity at higher exposure concentrations. The highest viability was observed in Quandong peach (88%), Kakadu plum (76%), Davidson plum (55%), and muntries (30%) at 240 mg/L while at 480 mg/L of fruit extracts the viability was 70%, 67%, 43% and 10% in Quandong peach, Kakadu plum, Davidson plum, and Muntries, respectively. Previously, Alafiatayo et al. [38] studied the toxicity of turmeric (*Curcuma longa*) through the embryonic zebrafish model. The methanolic extract of *Curcuma longa* at 62.5 μg/mL showed the teratogenic effect, while the higher concentrations (125 μg/mL) exhibited physical alterations and mortality. The LC_50_ 56.67 μg/mL of methanolic extract of turmeric was quantified at 96 h [38]. Previously, Wibowo et al. [39] reported the LC_50_ 196,037 ± 9.2 μg/mL for ethanolic extract of pomegranate at 96 h in zebrafish embryos. Moreover, Zhang et al. [40] quantified the LC_50_ values 2.78 ± 0.86 μg/mL and 6.62 ± 1.24 μg/mL for ethyl acetate extracts of *Euphorbia kansui* before (KS-1) and after fry-baked with vinegar (KS-2), respectively in the embryonic zebrafish model. They also reported cardiotoxicity and growth inhibition in zebrafish embryos after exposure to KS-1 and KS-2. Furthermore, they described that the toxicity decreased with the reduction of terpenoid constituents after the processing of *Euphorbia kansui* in vinegar [40]. The LC_50_ 345.6 mg/L of aqueous extracted safflower was quantified at 96 h which is lower than Davidson plum [41]. They also reported a significant change in developmental alterations due to safflower extract at the exposure of 250 mg/L after 48 and 72 h. The LC_50_ 10 μg/mL of Chinese motherwort (*Leonurus japonicus*) essential oil was quantified at 2, 10, and 24 h while LC_50_ around 60 μg/mL was quantified at 48 h by He et al. [42]. They also reported detrimental developmental alterations due to motherwort essential oil’s toxic effect. Moreover, the decocted extract (FZ-120) of fuzi (the lateral root of *Aconitum carmichaeli*) at the exposure of 700 μg/mL to 1000 μg/mL exhibited higher mortality rate while dose above 288 μg/mL showed abnormalities in the liver, heart, yolk sac absorption delay and swim bladder. Likewise, Ismail et al. [43] quantified the LC_50_ of water extracts of *Andrographis paniculata* (AP), *Curcuma xanthorrhiza* (CX), *Cinnamon zeylanicum* (CZ), *Eugenia polyantha* (EP), and *Orthosiphon stamineus* (OS) as 0.53 mg/mL, 0.70 mg/mL, 0.05 mg/mL, 0.06 mg/mL, 1.69 mg/mL, respectively at 96 h in zebrafish embryos. According to the Organization for Economic Cooperation and Development (OECD) guidelines (highly toxic; LC_50_ < 1 mg/L, toxic; 1 mg/L < LC_50_ > ten mg/L and harmful if LC_50_ is higher than ten mg/L while lower than 100 mg/L) as reported by Wibowo et al. [39] native Australian fruit extracts are included in safe category [39]. 

The high toxic level of muntries could be attributed to the saponins (soyasaponin ag) only being identified in Muntries. Previously, Kurt-Celep et al. [44] reported that saponin-rich extract was correlated with higher toxicity. They studied various parts of the same plant and reported that cytotoxicity level varies with plant parts. Previously, alkaloids had also been reported for higher toxicity [45]. Alkaloids including boldine, caffeoylcholine, glycocitridine, anabasamine, and convolidine were tentatively identified only in Muntries. The adverse effects of muntries could also be attributed to estragole (identified in Muntries only), a compound of known toxicity for *Drosophila melanogaster*, genotoxic and hepatocarcinogen, and forming DNA adducts in mouse liver [46,47]. The higher concentration of flavonoids could inactivate the toxicity of estragole [48,49]. It is important to mention that the toxic effects observed in zebrafish larvae exposed to Muntries, Kakadu plum, Davidson plum and Quandong peach extracts could also be explained by the synergistic interactions of phytochemical metabolites which can alter or intensify the potential of the target bioactive compounds. 

Virtual toxicological screening of abundant phytochemical metabolites is given in Table S4. The predicted results indicate that all selected phytochemical metabolites do not inhibit the hERG I channel while compounds; caffeine, anabasamine, convolidine, corosolic acid, and umbelliferone have hepatoxicity potential. Caffeoylcholine, boldine, glycocitridine, longifolene, and resveratrol predicted toxicity in *Tetrahymena pyriformis* while longifolene, corosolic acid, and hoslundal were predicted toxicity in minnow fish. Quinic acid, longifolene, 3-*p*-coumaroylquinic acid, fraxetin, and estragole were predicted to have the highest oral rate acute toxicity, while estragole, diphyllin, and resveratrol were also predicted toxicity in AEMS toxicity model (Table S4) which predicts the mutagenicity of the compounds. Interestingly, quinic acid, fraxetin, estragole, corosolic acid, 3-ethylphenol, 4-vinylphenol, caffeoylcholine, boldine, glycocitridine, longifolene and 3-*p*-coumaroylquinic acid were identified in Muntries (Table S2). This highest toxicity effect of Muntries could be due to the presence of these phytochemical metabolites in Muntries extract. Furthermore, in silico docking results (Table S5) predicted that rutin and delphinidin 3-sambubioside have higher hepaprotective potential compared to all selected phytochemical metabolites. 

The evaluation of drug toxicity and safety is critical for drug discovery. The toxicological effects of fruit extracts and their isolated phytochemical metabolites could limit their applicability to pharmaceutical and pharmacological applications in drug discovery. Therefore, it is a prerequisite to test the toxicity of plant extracts and isolated bioactive compounds through different preclinical models to achieve the target potential of the bioactive compounds. Zebrafish embryonic model is a well-established animal model for the early assessment of drug toxicity compared to rodents and other large animal models. The results of this study contribute to understanding the toxicity of native Australian fruit extracts which have a predictive value about their safety. 

## 4. Conclusions

In this study, we identified a total of 427 phenolic and non-phenolic metabolites, including 64 phenolic acids, 238 flavonoids, isoflavonoids, ten tannins, ten stilbenes, 27 lignans, 61 other polyphenols, 17 non-phenolic metabolites with the help of LC-ESI-QTOF-MS. The results indicate that muntries showed higher mortality at 96 h than other selected fruits, while Kakadu plum has higher total phenolic content and antioxidant potential, meaning toxicity is not correlated to total phenolic content. This toxicity might be due to other individual phytochemicals, including saponins, alkaloids, or some phenolic metabolites in Muntries. The abundance and diversity of these phytochemicals in native Australian fruits might have distinctive benefits for different pathologies. The comparative profiling of these fruits for phytochemicals and toxicological screening will allow us in the future to distinguish among phytochemicals, which combinations influence the LC_50_ to derive out more toxic compounds. Moreover, these native Australian fruit extracts are categorized as safe as described in OECD guidelines.

## Data Availability

The supporting data are provided in the Supplementary Materials.

## Supplementary Materials

The following supporting information can be downloaded at: https://www.mdpi.com/article/10.3390/foods11244038/s1, Figure S1: Chromatograms and mass spectra of some selected compounds; Table S1: Quantification of phenolic contents and antioxidant activities of native Australian fruits; Table S2: Phytochemical screening of native Australian fruits through LC-ESI-QTOF-MS; Table S3: LC-MS semi-quantification of abundant phenolic metabolites (%); Table S4: Virtual toxicological screening of most abundant phytochemical metabolites in native Australian fruits; Table S5: The estimated docking score (kcal/mol) and glide energy (kcal/mol) of phenolic metabolites in alanine aminotransferase (3IHJ).
