# Supplementary material for: Phytochemicals, Antioxidant Activities, and Toxicological Screening of Native Australian Fruits Using Zebrafish Embryonic Model
Source: Foods. 2022 Dec 14;11(24):4038. doi: 10.3390/foods11244038 (PMC9777714; doi:10.3390/foods11244038)
Supplement: Supplementary file 1 [file foods-11-04038-s001.zip › foods-1995348-supplementary.pdf]

Supplementary Material

Phytochemicals, antioxidant activities, and toxicological screening of native Australian fruits using Zebrafish embryonic model

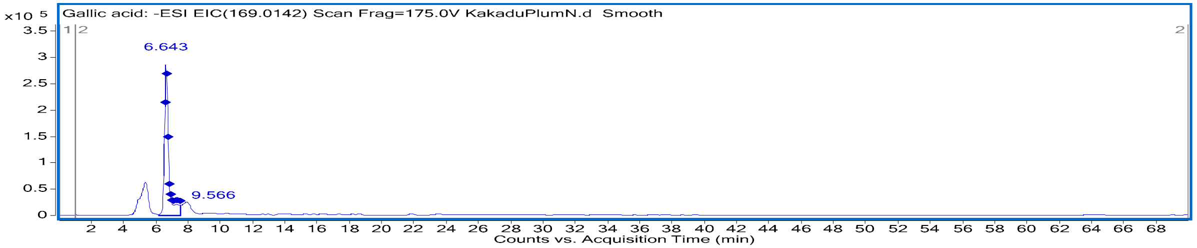

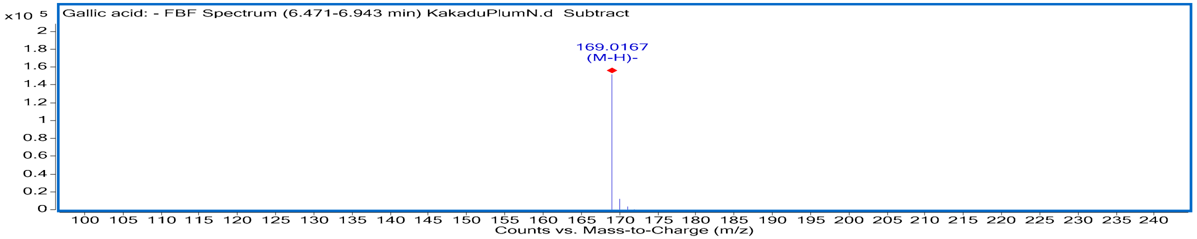

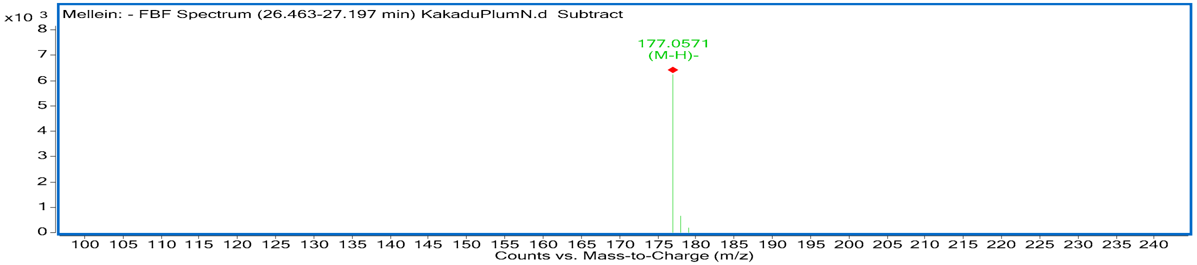

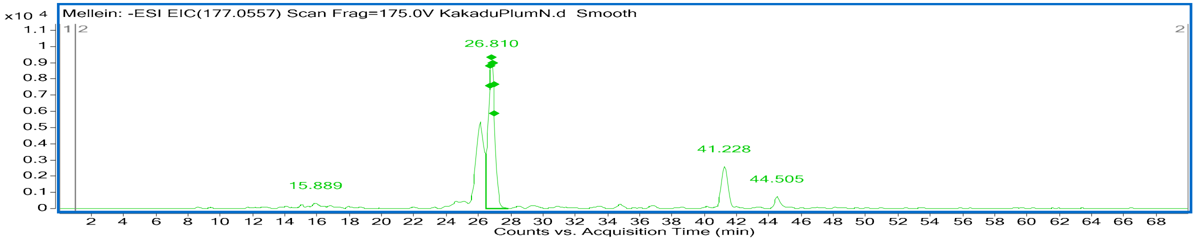

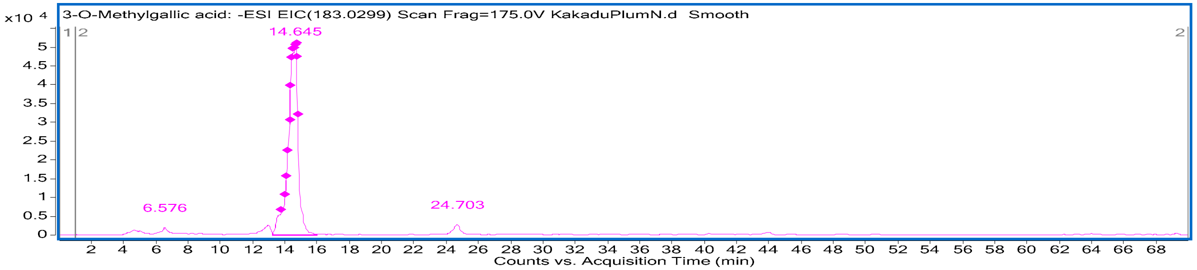
3-O-Methylgallic acid: -ESI EIC(183.0299) Scan Frag=175.0V KakaduPlumN.d Smooth
x10 4
14.645
6.576
24.703
Counts vs. Acquisition Time (min)

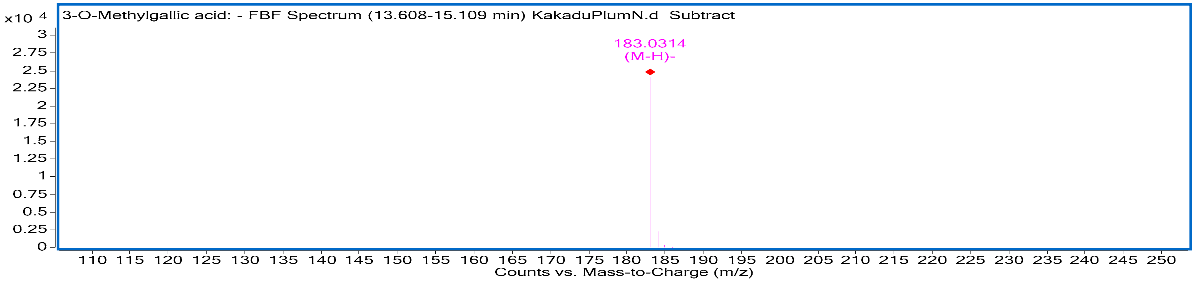
3-O-Methylgallic acid: - FBF Spectrum (13.608-15.109 min) KakaduPlumN.d Subtract
x10 4
183.0314
(M-H)-
Counts vs. Mass-to-Charge (m/z)

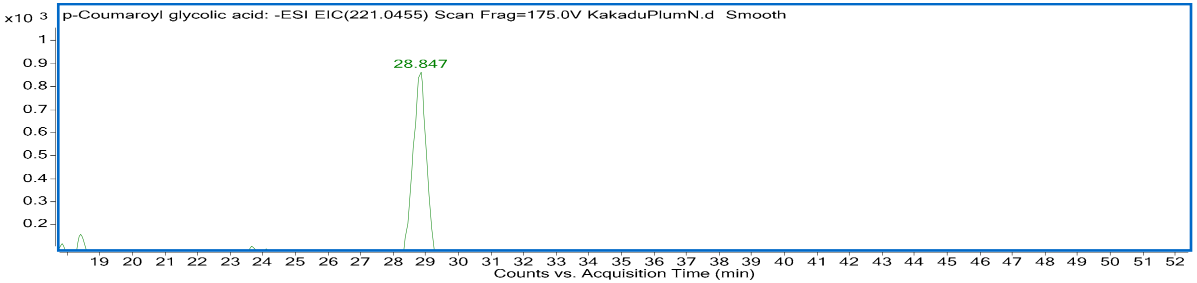
p-Coumaroyl glycolic acid: -ESI EIC(221.0455) Scan Frag=175.0V KakaduPlumN.d Smooth
x10 3
28.847
Counts vs. Acquisition Time (min)

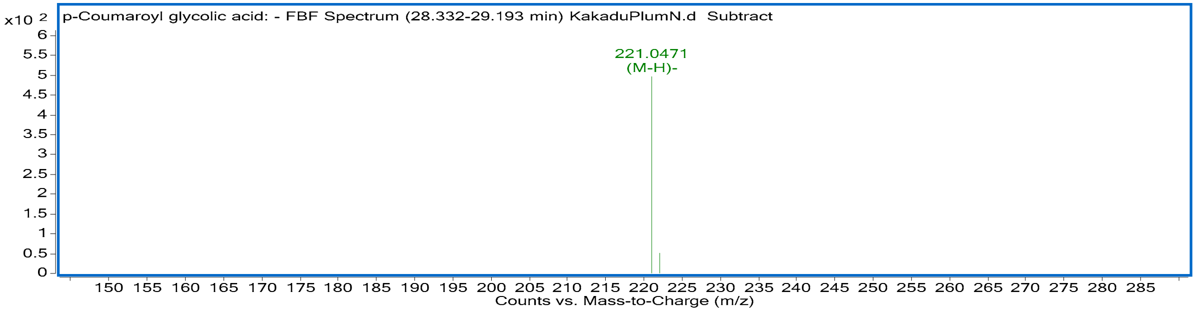
p-Coumaroyl glycolic acid: - FBF Spectrum (28.332-29.193 min) KakaduPlumN.d Subtract
x10 2
221.0471
(M-H)-
Counts vs. Mass-to-Charge (m/z)

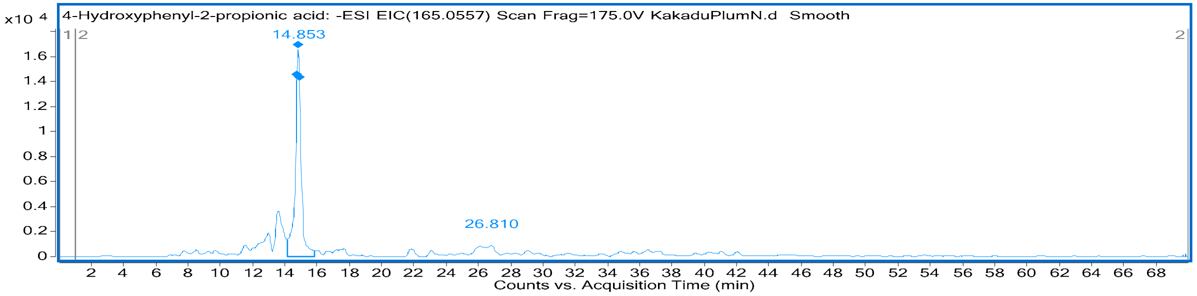
4-Hydroxyphenyl-2-propionic acid: -ESI EIC(165.0557) Scan Frag=175.0V KakaduPlumN.d Smooth
x10 4
14.853
26.810
Counts vs. Acquisition Time (min)

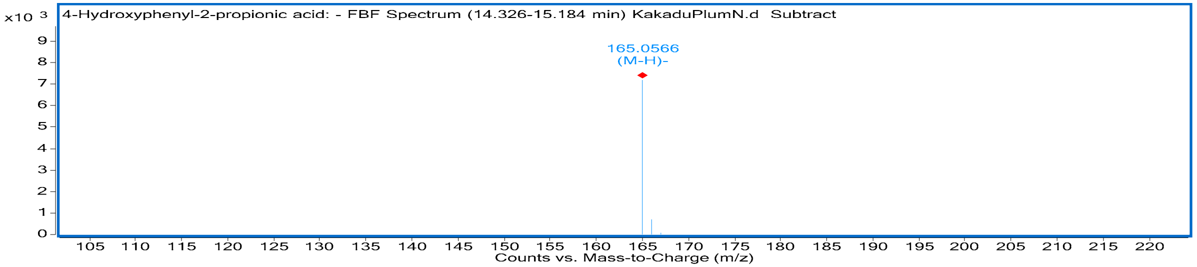
4-Hydroxyphenyl-2-propionic acid: - FBF Spectrum (14.326-15.184 min) KakaduPlumN.d Subtract
x10 3
165.0566
(M-H)-
Counts vs. Mass-to-Charge (m/z)

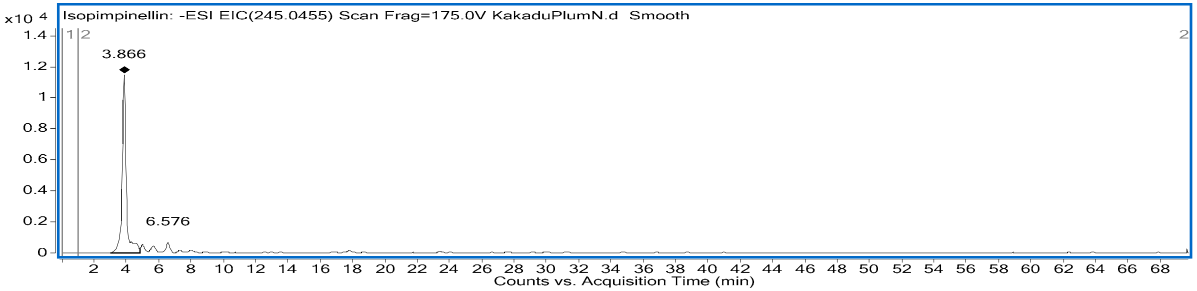
Isopimpinellin: -ESI EIC(245.0455) Scan Frag=175.0V KakaduPlumN.d Smooth
x10 4
3.866
6.576
Counts vs. Acquisition Time (min)

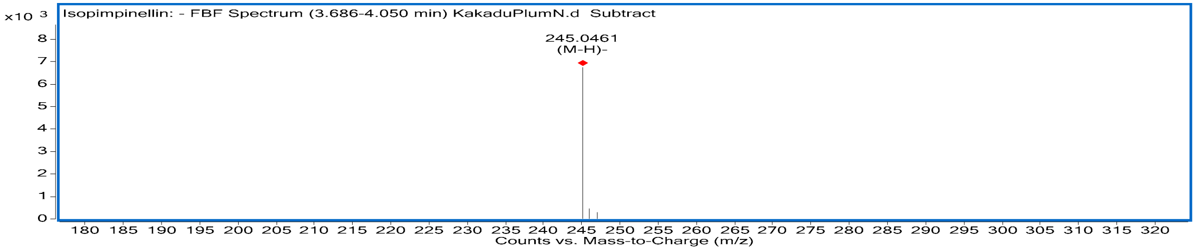
Isopimpinellin: - FBF Spectrum (3.686-4.050 min) KakaduPlumN.d Subtract
x10 3
245.0461
(M-H)-
Counts vs. Mass-to-Charge (m/z)

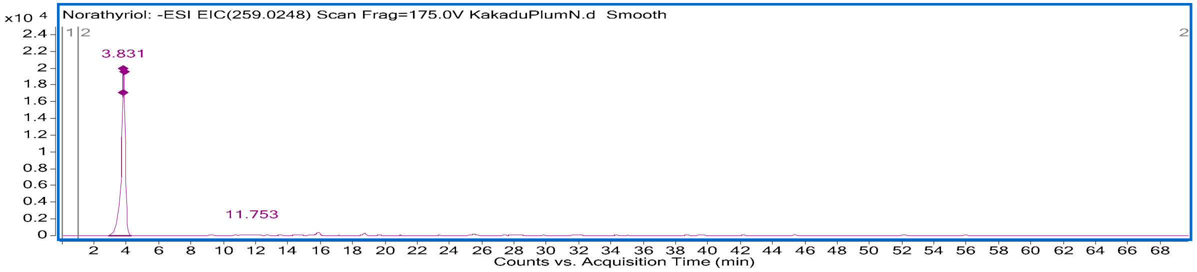
Norathyriol: -ESI EIC(259.0248) Scan Frag=175.0V KakaduPlumN.d Smooth
x10 4
3.831
11.753
Counts vs. Acquisition Time (min)

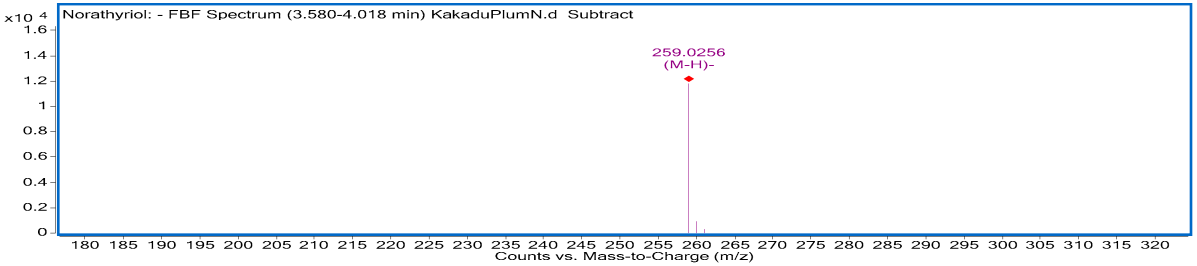
Norathyriol: - FBF Spectrum (3.580-4.018 min) KakaduPlumN.d Subtract
x10 4
259.0256
(M-H)-
Counts vs. Mass-to-Charge (m/z)

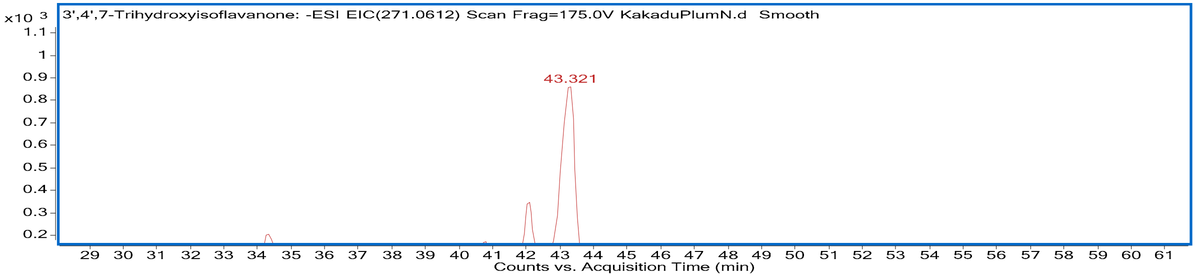
3',4',7-Trihydroxyisoflavanone: -ESI EIC(271.0612) Scan Frag=175.0V KakaduPlumN.d Smooth
x10 3
43.321
Counts vs. Acquisition Time (min)

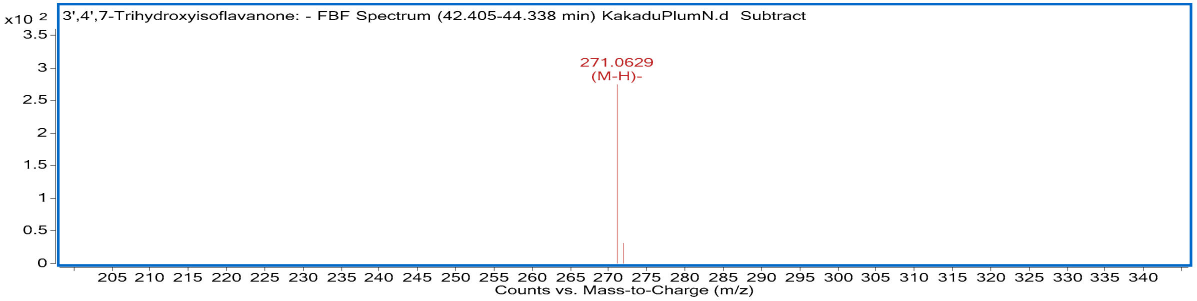
3',4',7-Trihydroxyisoflavanone: - FBF Spectrum (42.405-44.338 min) KakaduPlumN.d Subtract
x10 2
271.0629
(M-H)-
Counts vs. Mass-to-Charge (m/z)

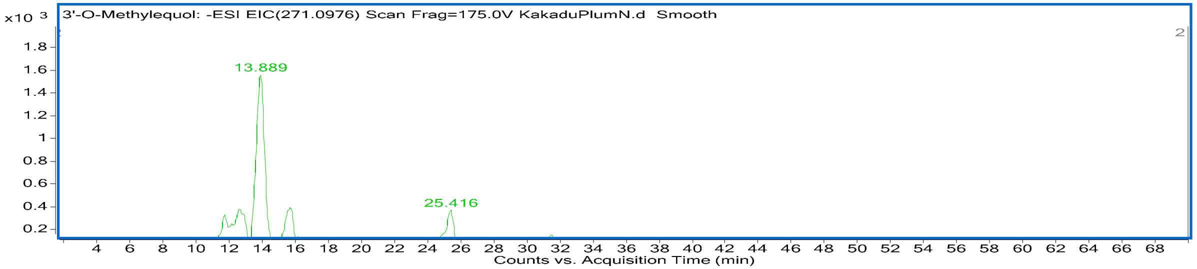
3'-O-Methylequol: -ESI EIC(271.0976) Scan Frag=175.0V KakaduPlumN.d Smooth
x10 3
13.889
25.416
Counts vs. Acquisition Time (min)

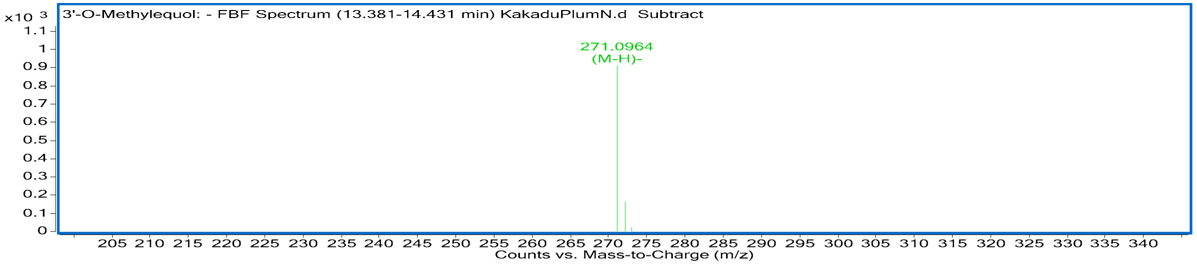
3'-O-Methylequol: - FBF Spectrum (13.381-14.431 min) KakaduPlumN.d Subtract
x10 3
271.0964
(M-H)-
Counts vs. Mass-to-Charge (m/z)

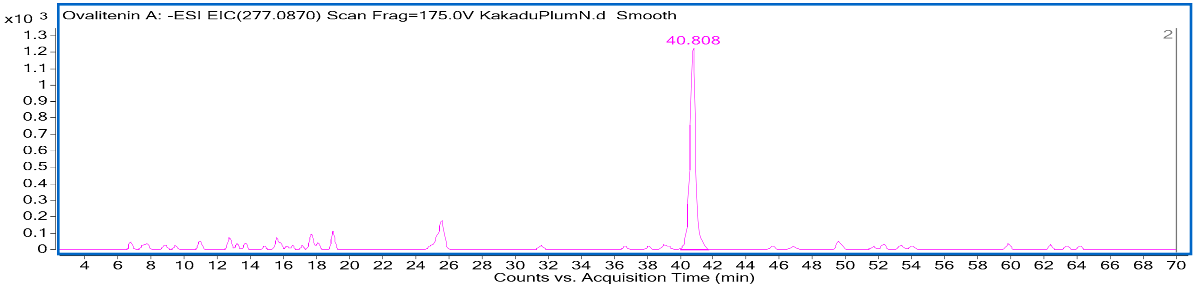
Ovalitenin A: -ESI EIC(277.0870) Scan Frag=175.0V KakaduPlumN.d Smooth
x10 3
40.808
Counts vs. Acquisition Time (min)

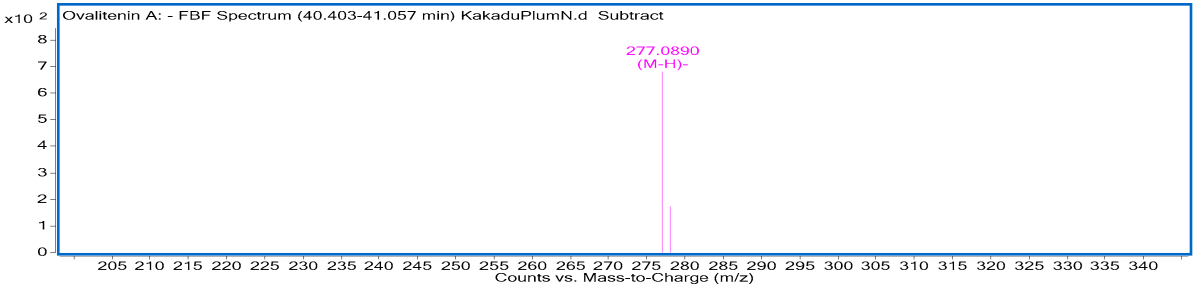
Ovalitenin A: - FBF Spectrum (40.403-41.057 min) KakaduPlumN.d Subtract
x10 2
277.0890
(M-H)-
Counts vs. Mass-to-Charge (m/z)

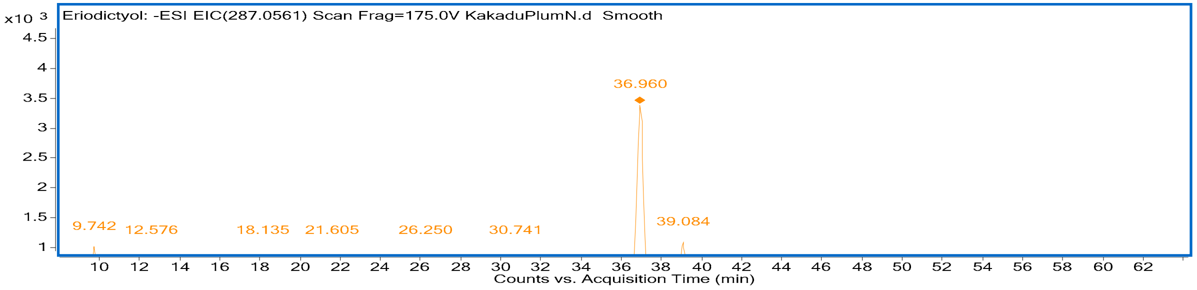
x10 3
Eriodictyol: -ESI EIC(287.0561) Scan Frag=175.0V KakaduPlumN.d Smooth
36.960
9.742
12.576
18.135
21.605
26.250
30.741
39.084
Counts vs. Acquisition Time (min)

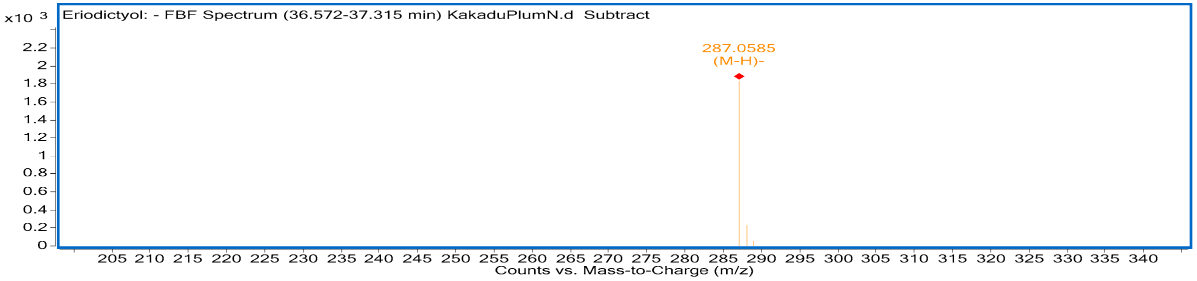
x10 3
Eriodictyol: - FBF Spectrum (36.572-37.315 min) KakaduPlumN.d Subtract
287.0585
(M-H)-
Counts vs. Mass-to-Charge (m/z)

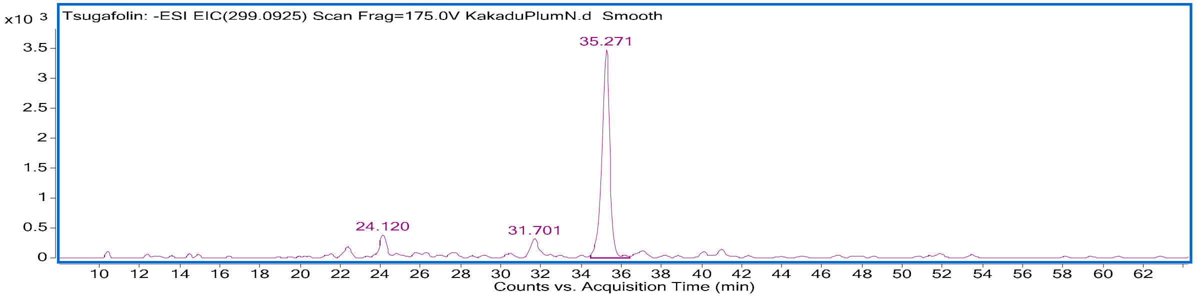
x10 3
Tsugafolin: -ESI EIC(299.0925) Scan Frag=175.0V KakaduPlumN.d Smooth
35.271
24.120
31.701
Counts vs. Acquisition Time (min)

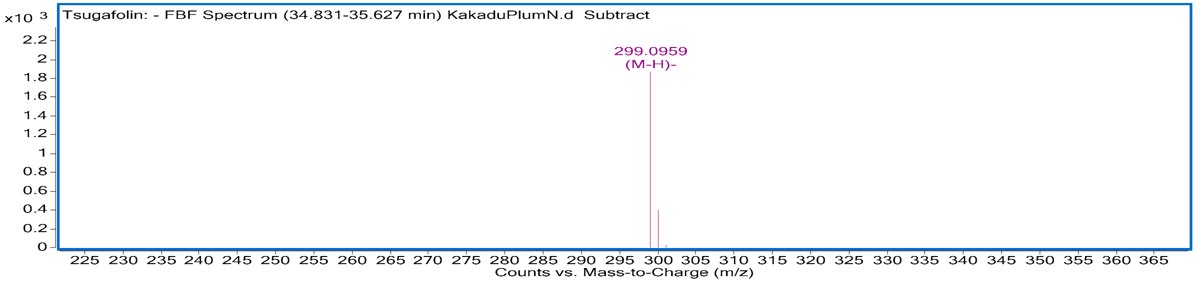
x10 3
Tsugafolin: - FBF Spectrum (34.831-35.627 min) KakaduPlumN.d Subtract
299.0959
(M-H)-
Counts vs. Mass-to-Charge (m/z)

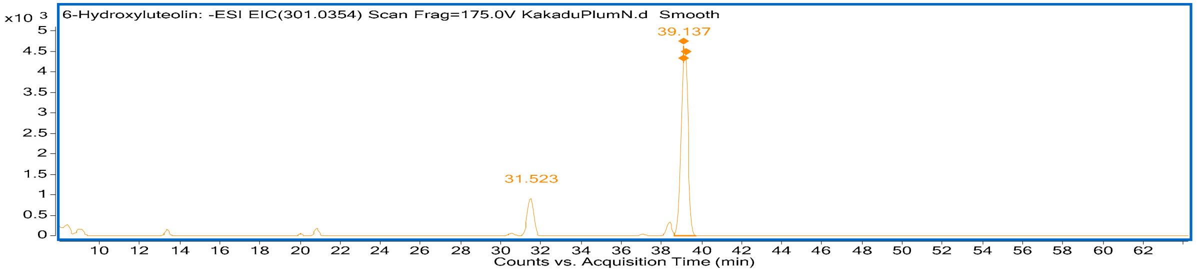
x10 3
6-Hydroxyluteolin: -ESI EIC(301.0354) Scan Frag=175.0V KakaduPlumN.d Smooth
39.137
31.523
Counts vs. Acquisition Time (min)

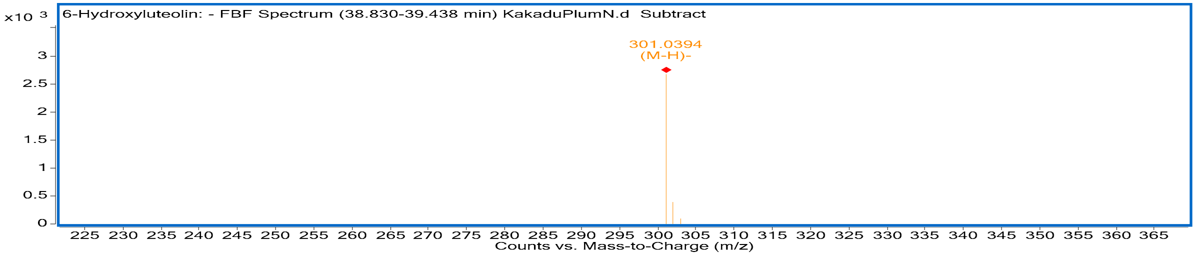
x10 3
6-Hydroxyluteolin: - FBF Spectrum (38.830-39.438 min) KakaduPlumN.d Subtract
301.0394
(M-H)-
Counts vs. Mass-to-Charge (m/z)

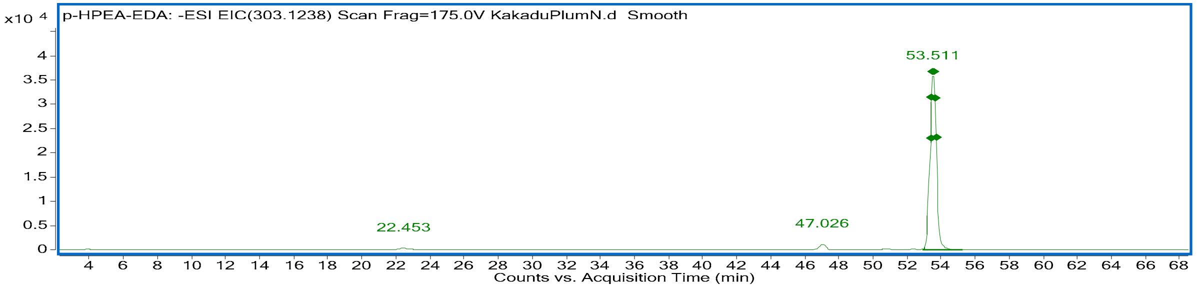
x10 4
p-HPEA-EDA: -ESI EIC(303.1238) Scan Frag=175.0V KakaduPlumN.d Smooth
53.511
22.453
47.026
Counts vs. Acquisition Time (min)

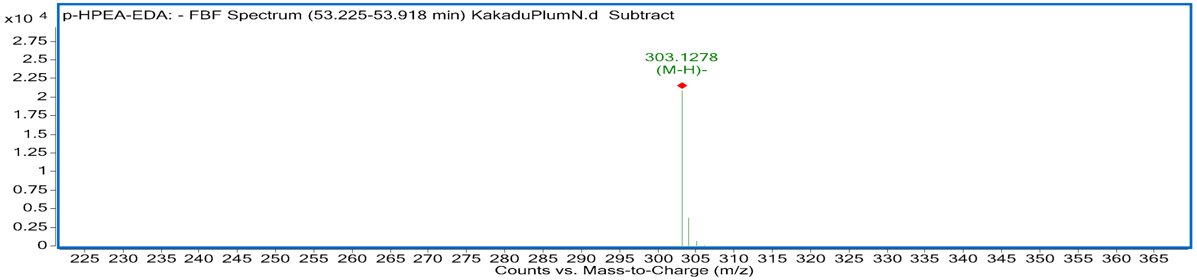
x10 4
p-HPEA-EDA: - FBF Spectrum (53.225-53.918 min) KakaduPlumN.d Subtract
303.1278
(M-H)-
Counts vs. Mass-to-Charge (m/z)

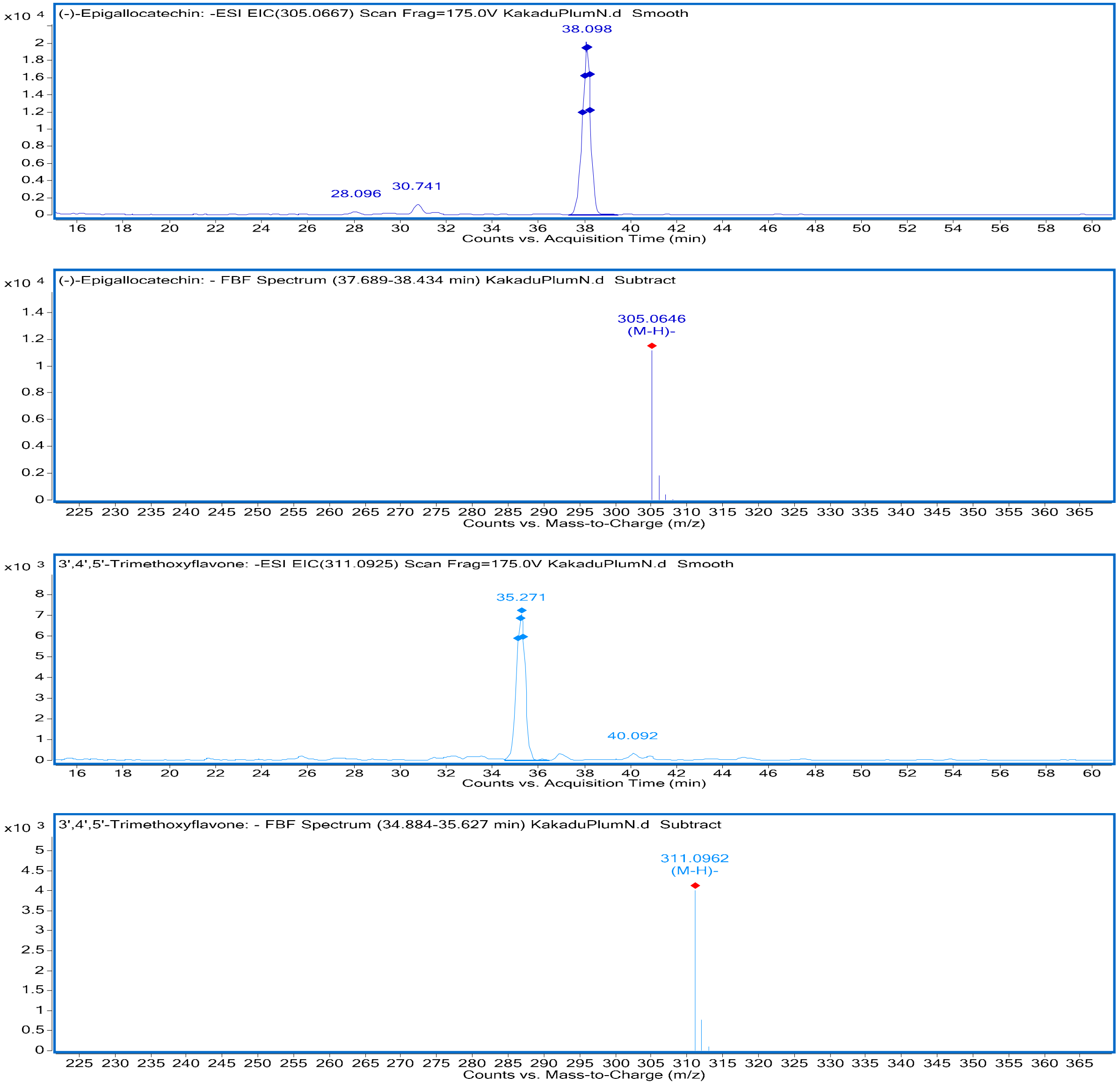
x10 4
(-)-Epigallocatechin: -ESI EIC(305.0667) Scan Frag=175.0V KakaduPlumN.d Smooth
38.098
28.096
30.741
Counts vs. Acquisition Time (min)
x10 4
(-)-Epigallocatechin: - FBF Spectrum (37.689-38.434 min) KakaduPlumN.d Subtract
305.0646
(M-H)-
Counts vs. Mass-to-Charge (m/z)
x10 3
3',4',5'-Trimethoxyflavone: -ESI EIC(311.0925) Scan Frag=175.0V KakaduPlumN.d Smooth
35.271
40.092
Counts vs. Acquisition Time (min)
x10 3
3',4',5'-Trimethoxyflavone: - FBF Spectrum (34.884-35.627 min) KakaduPlumN.d Subtract
311.0962
(M-H)-
Counts vs. Mass-to-Charge (m/z)

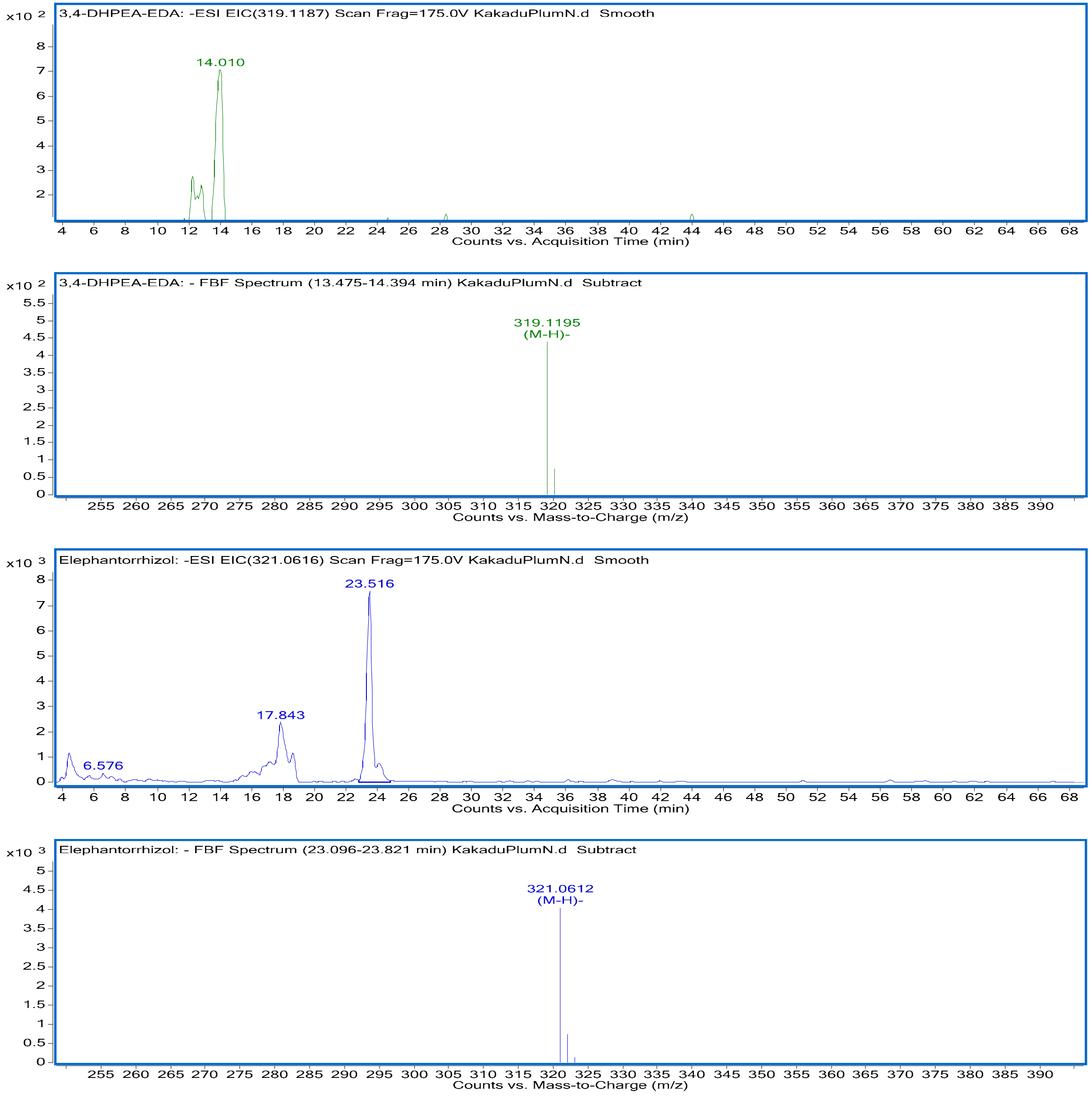

3,4-DHPEA-EDA: -ESI EIC(319.1187) Scan Frag=175.0V KakaduPlumN.d Smooth
x10 2
14.010
Counts vs. Acquisition Time (min)
3,4-DHPEA-EDA: - FBF Spectrum (13.475-14.394 min) KakaduPlumN.d Subtract
x10 2
319.1195
(M-H)-
Counts vs. Mass-to-Charge (m/z)
Elephantorrhizol: -ESI EIC(321.0616) Scan Frag=175.0V KakaduPlumN.d Smooth
x10 3
23.516
17.843
6.576
Counts vs. Acquisition Time (min)
Elephantorrhizol: - FBF Spectrum (23.096-23.821 min) KakaduPlumN.d Subtract
x10 3
321.0612
(M-H)-
Counts vs. Mass-to-Charge (m/z)

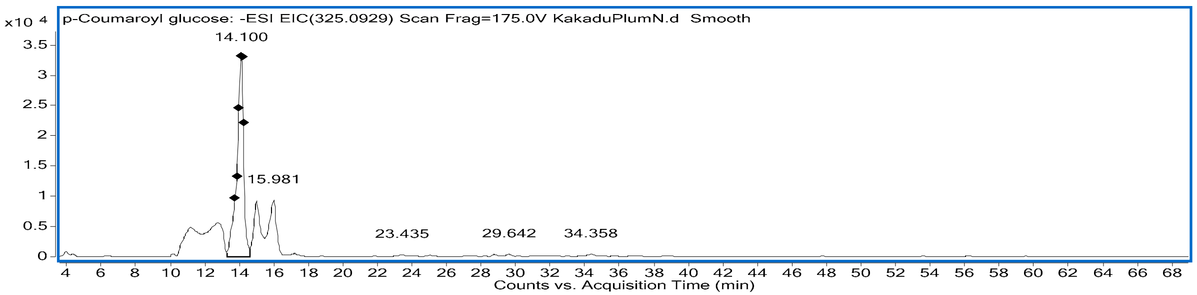
p-Coumaroyl glucose: -ESI EIC(325.0929) Scan Frag=175.0V KakaduPlumN.d Smooth
x10 4
14.100
15.981
23.435
29.642
34.358
Counts vs. Acquisition Time (min)

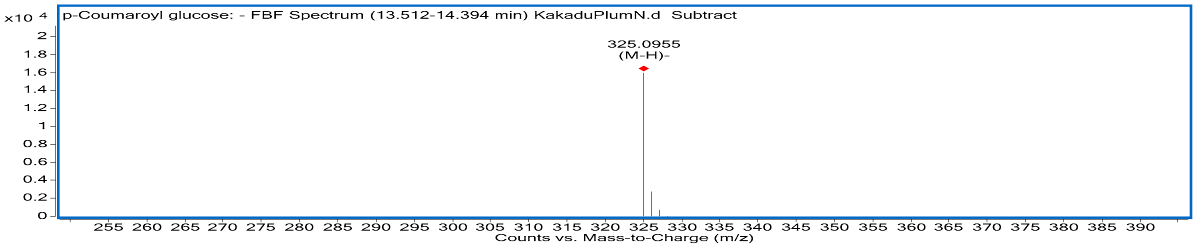
p-Coumaroyl glucose: - FBF Spectrum (13.512-14.394 min) KakaduPlumN.d Subtract
x10 4
325.0955
(M-H)-
Counts vs. Mass-to-Charge (m/z)

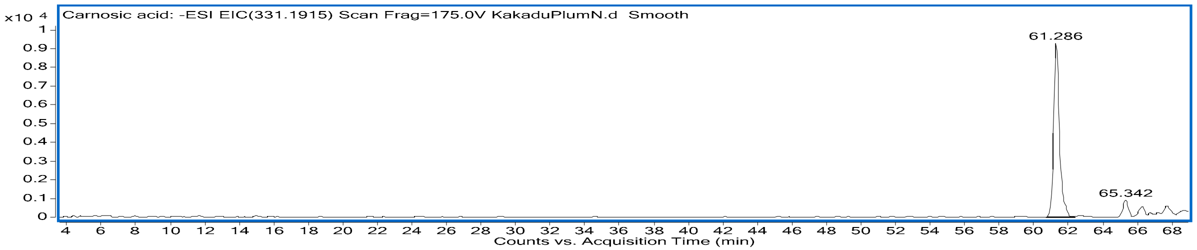
Carnosic acid: -ESI EIC(331.1915) Scan Frag=175.0V KakaduPlumN.d Smooth
x10 4
61.286
65.342
Counts vs. Acquisition Time (min)

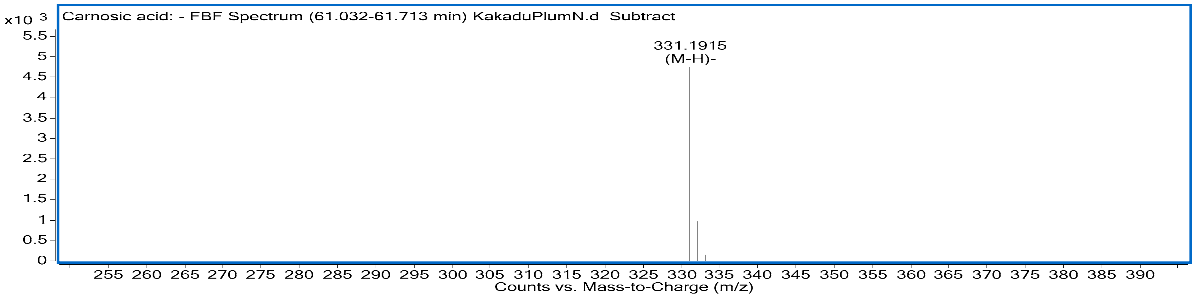
Carnosic acid: - FBF Spectrum (61.032-61.713 min) KakaduPlumN.d Subtract
x10 3
331.1915
(M-H)-
Counts vs. Mass-to-Charge (m/z)

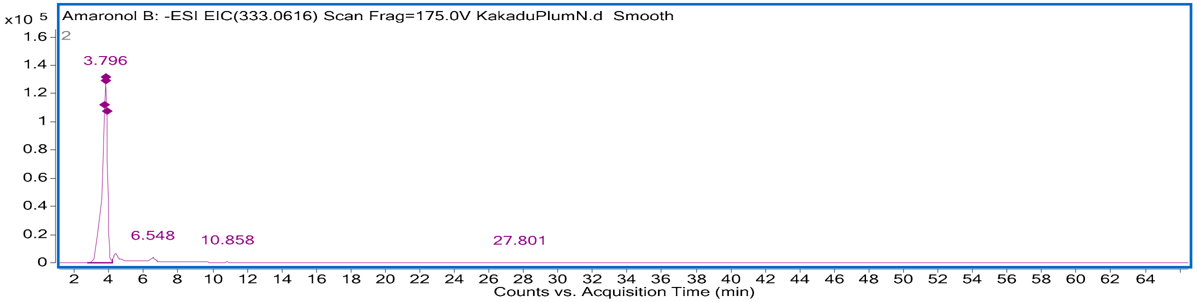
Amaronol B: -ESI EIC(333.0616) Scan Frag=175.0V KakaduPlumN.d Smooth
x10 5
3.796
6.548
10.858
27.801
Counts vs. Acquisition Time (min)

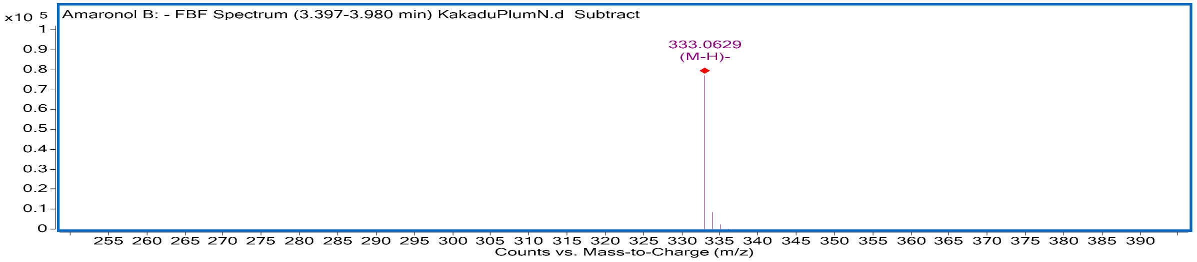
Amaronol B: - FBF Spectrum (3.397-3.980 min) KakaduPlumN.d Subtract
x10 5
333.0629
(M-H)-
Counts vs. Mass-to-Charge (m/z)

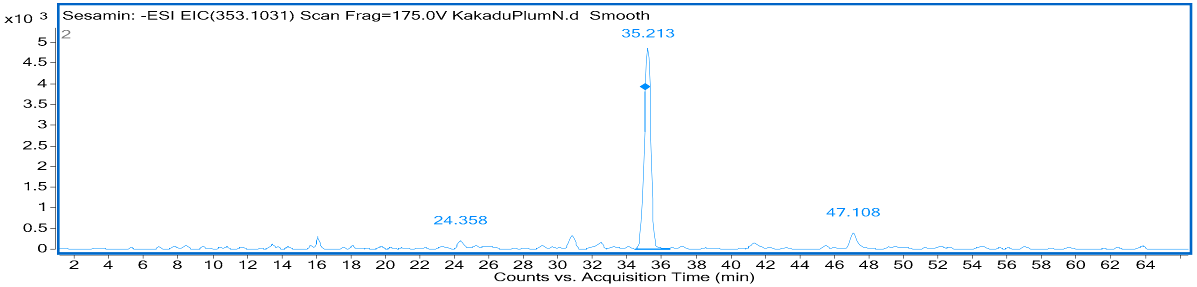
Sesamin: -ESI EIC(353.1031) Scan Frag=175.0V KakaduPlumN.d Smooth
x10 3
35.213
24.358
47.108
Counts vs. Acquisition Time (min)

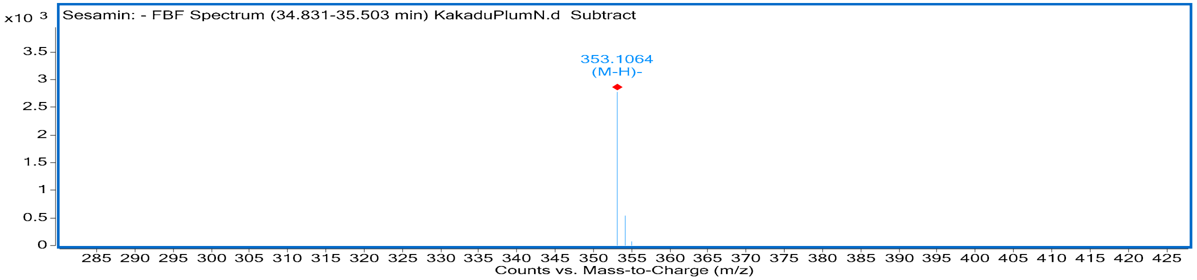
Sesamin: - FBF Spectrum (34.831-35.503 min) KakaduPlumN.d Subtract
x10 3
353.1064
(M-H)-
Counts vs. Mass-to-Charge (m/z)

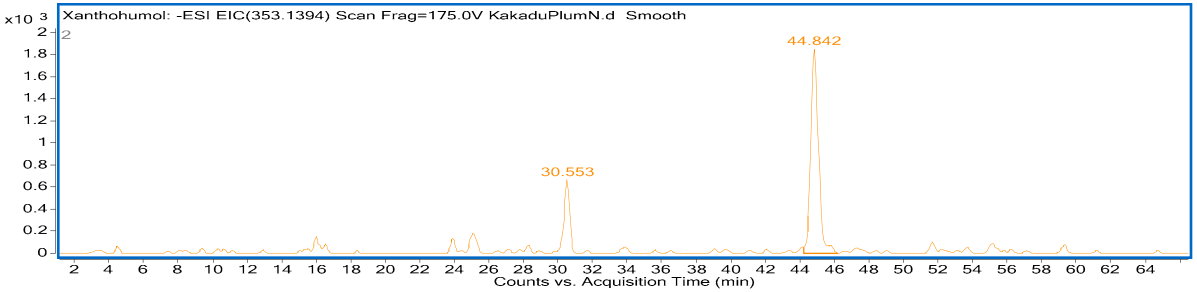
Xanthohumol: -ESI EIC(353.1394) Scan Frag=175.0V KakaduPlumN.d Smooth
x10 3
44.842
30.553
Counts vs. Acquisition Time (min)

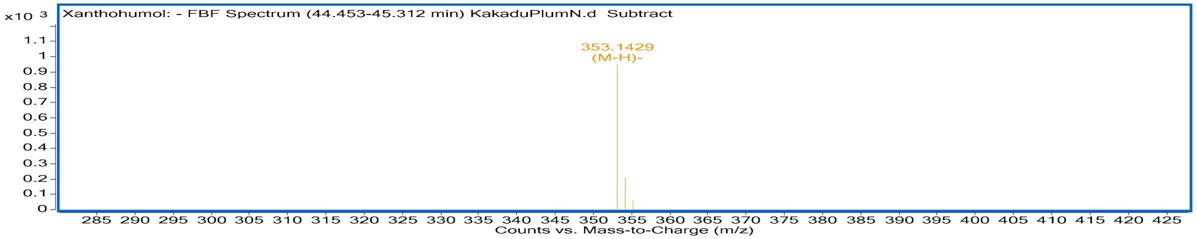
Xanthohumol: - FBF Spectrum (44.453-45.312 min) KakaduPlumN.d Subtract
x10 3
353.1429
(M-H)-
Counts vs. Mass-to-Charge (m/z)

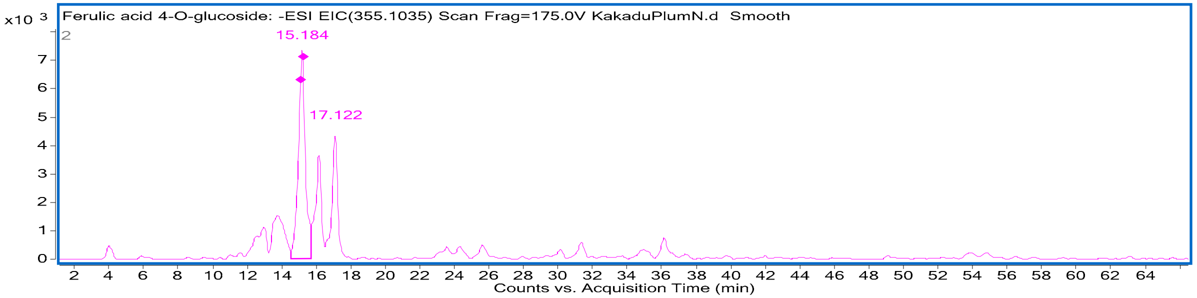
Ferulic acid 4-O-glucoside: -ESI EIC(355.1035) Scan Frag=175.0V KakaduPlumN.d Smooth
x10 3
15.184
17.122
Counts vs. Acquisition Time (min)

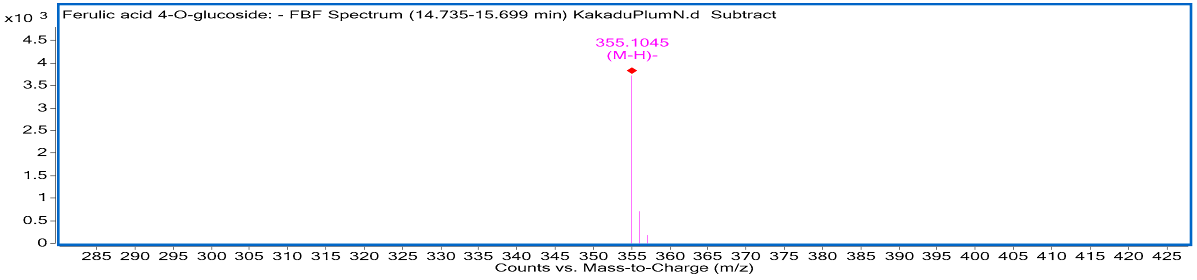
Ferulic acid 4-O-glucoside: - FBF Spectrum (14.735-15.699 min) KakaduPlumN.d Subtract
x10 3
355.1045
(M-H)-
Counts vs. Mass-to-Charge (m/z)

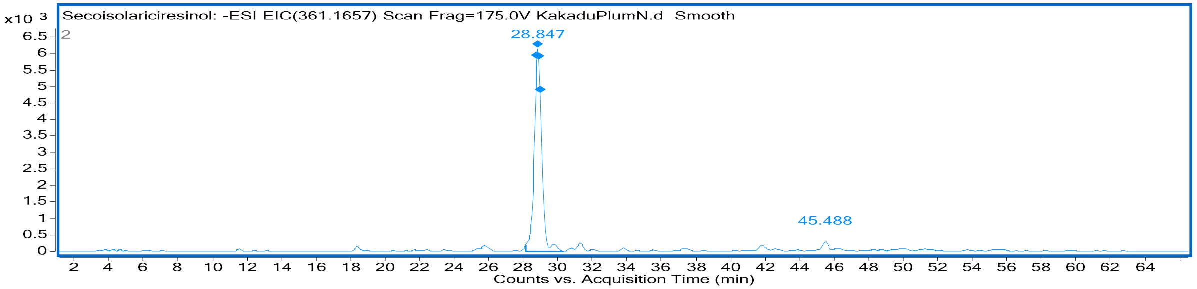
x10 3
Secoisolariciresinol: -ESI EIC(361.1657) Scan Frag=175.0V KakaduPlumN.d Smooth
28.847
45.488
Counts vs. Acquisition Time (min)

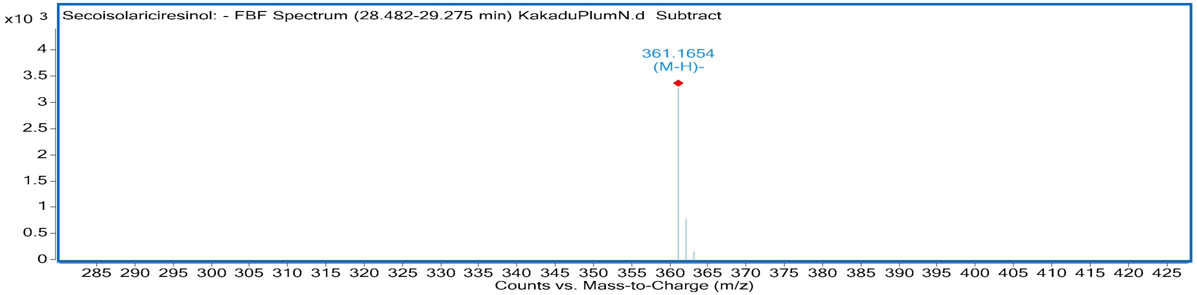
x10 3
Secoisolariciresinol: - FBF Spectrum (28.482-29.275 min) KakaduPlumN.d Subtract
361.1654
(M-H)-
Counts vs. Mass-to-Charge (m/z)

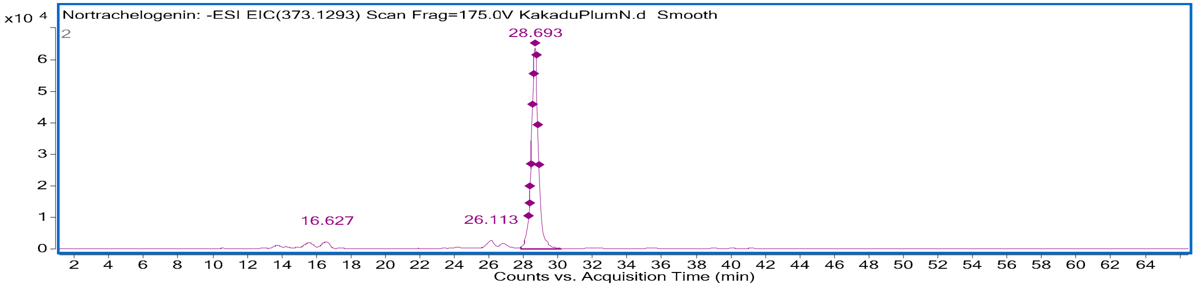
x10 4
Nortrachelogenin: -ESI EIC(373.1293) Scan Frag=175.0V KakaduPlumN.d Smooth
28.693
16.627
26.113
Counts vs. Acquisition Time (min)

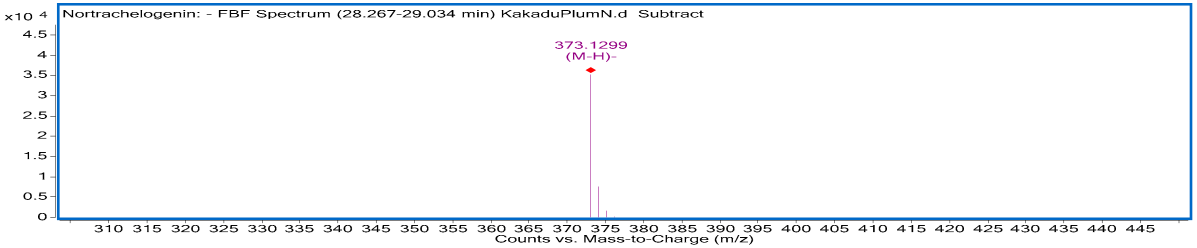
x10 4
Nortrachelogenin: - FBF Spectrum (28.267-29.034 min) KakaduPlumN.d Subtract
373.1299
(M-H)-
Counts vs. Mass-to-Charge (m/z)

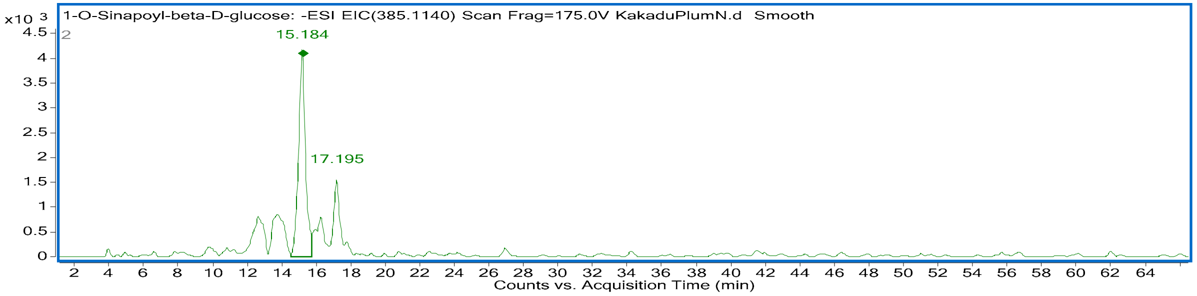
x10 3
1-O-Sinapoyl-beta-D-glucose: -ESI EIC(385.1140) Scan Frag=175.0V KakaduPlumN.d Smooth
15.184
17.195
Counts vs. Acquisition Time (min)

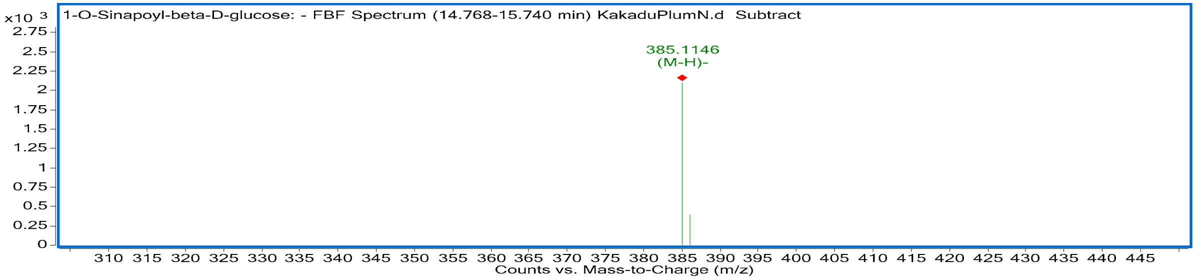
x10 3
1-O-Sinapoyl-beta-D-glucose: - FBF Spectrum (14.768-15.740 min) KakaduPlumN.d Subtract
385.1146
(M-H)-
Counts vs. Mass-to-Charge (m/z)

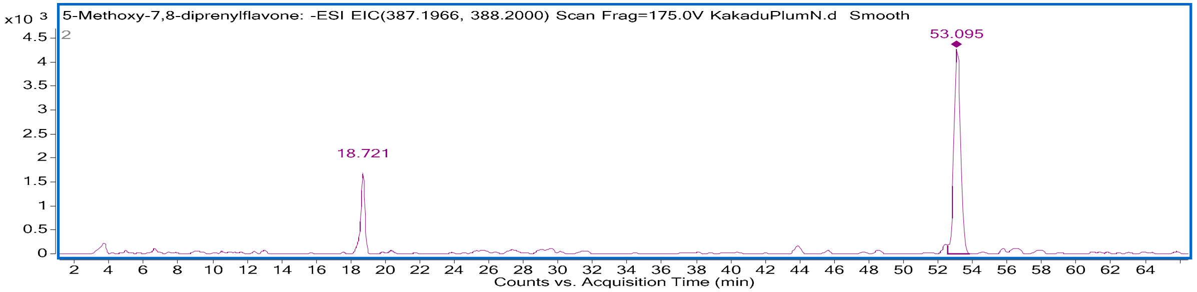
x10 3
5-Methoxy-7,8-diprenylflavone: -ESI EIC(387.1966, 388.2000) Scan Frag=175.0V KakaduPlumN.d Smooth
53.095
18.721
Counts vs. Acquisition Time (min)

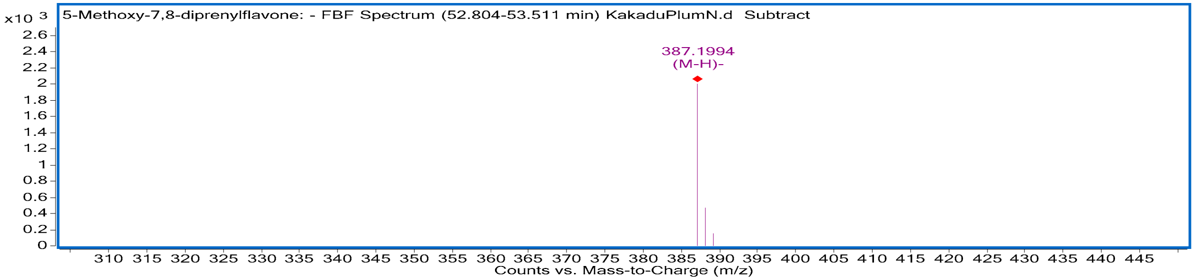
x10 3
5-Methoxy-7,8-diprenylflavone: - FBF Spectrum (52.804-53.511 min) KakaduPlumN.d Subtract
387.1994
(M-H)-
Counts vs. Mass-to-Charge (m/z)

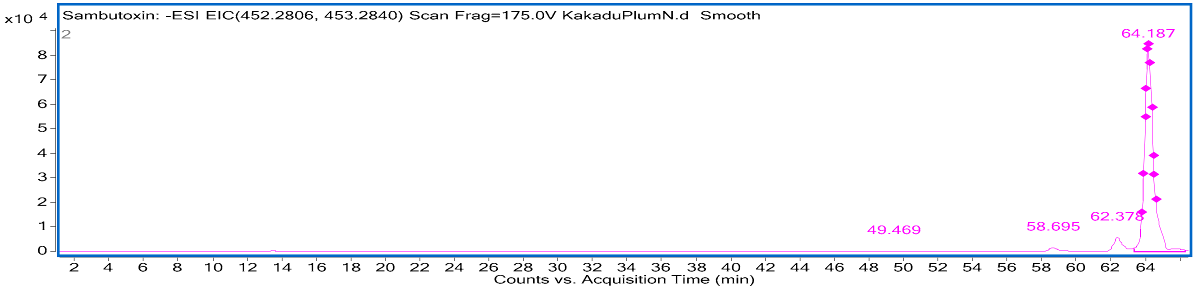
x10 4
Sambutoxin: -ESI EIC(452.2806, 453.2840) Scan Frag=175.0V KakaduPlumN.d Smooth
64.187
62.378
58.695
49.469
Counts vs. Acquisition Time (min)

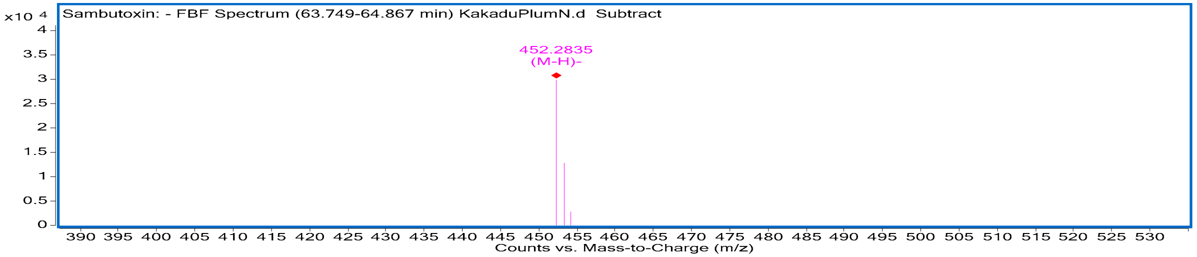
x10 4
Sambutoxin: - FBF Spectrum (63.749-64.867 min) KakaduPlumN.d Subtract
452.2835
(M-H)-
Counts vs. Mass-to-Charge (m/z)

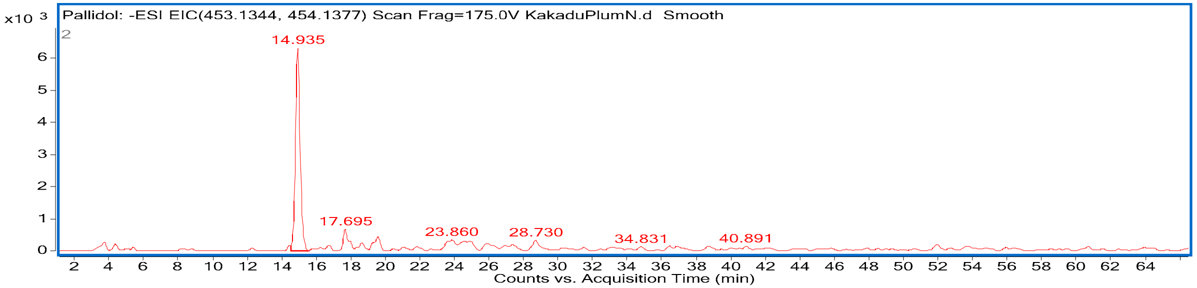
x10 3
Pallidol: -ESI EIC(453.1344, 454.1377) Scan Frag=175.0V KakaduPlumN.d Smooth
14.935
17.695
23.860
28.730
34.831
40.891
Counts vs. Acquisition Time (min)

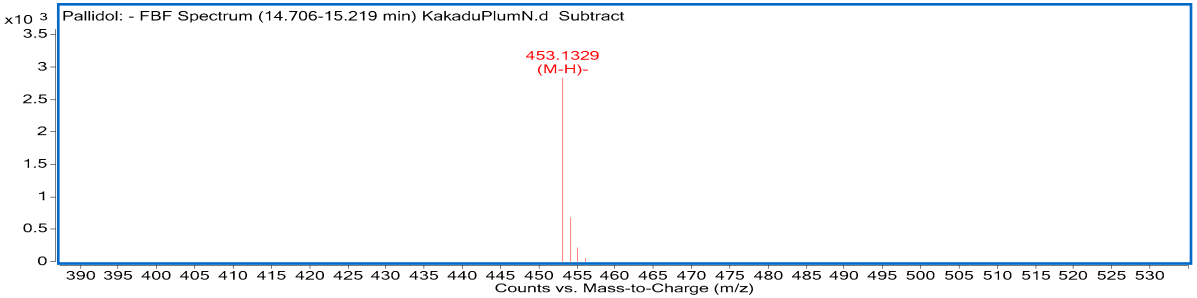
x10 3
Pallidol: - FBF Spectrum (14.706-15.219 min) KakaduPlumN.d Subtract
453.1329
(M-H)-
Counts vs. Mass-to-Charge (m/z)

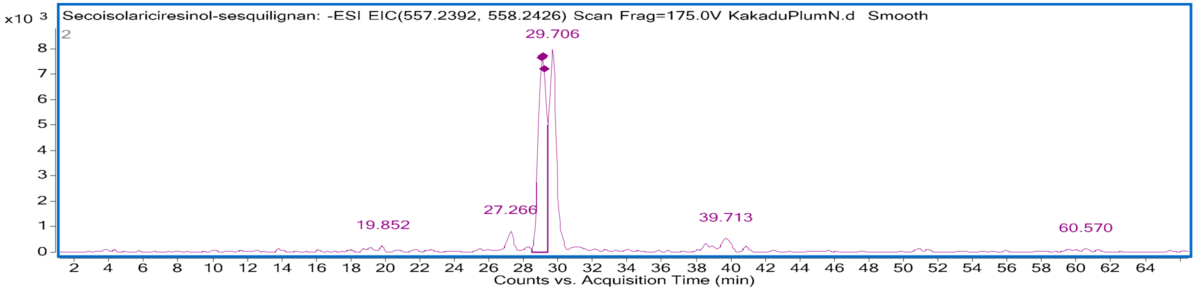
x10 3
Secoisolariciresinol-sesquilignan: -ESI EIC(557.2392, 558.2426) Scan Frag=175.0V KakaduPlumN.d Smooth
29.706
27.266
19.852
39.713
60.570
Counts vs. Acquisition Time (min)

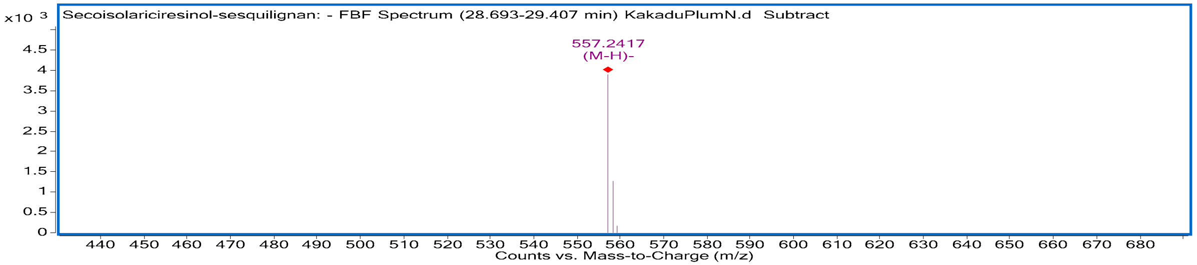
x10 3
Secoisolariciresinol-sesquilignan: - FBF Spectrum (28.693-29.407 min) KakaduPlumN.d Subtract
557.2417
(M-H)-
Counts vs. Mass-to-Charge (m/z)

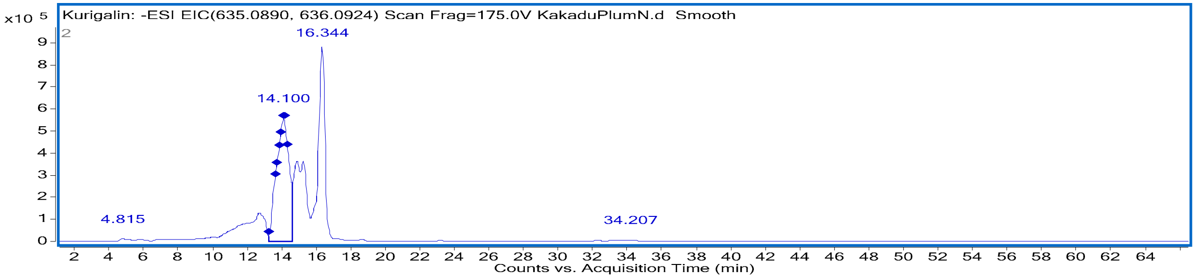
x10 5
Kurigalin: -ESI EIC(635.0890, 636.0924) Scan Frag=175.0V KakaduPlumN.d Smooth
16.344
14.100
4.815
34.207
Counts vs. Acquisition Time (min)

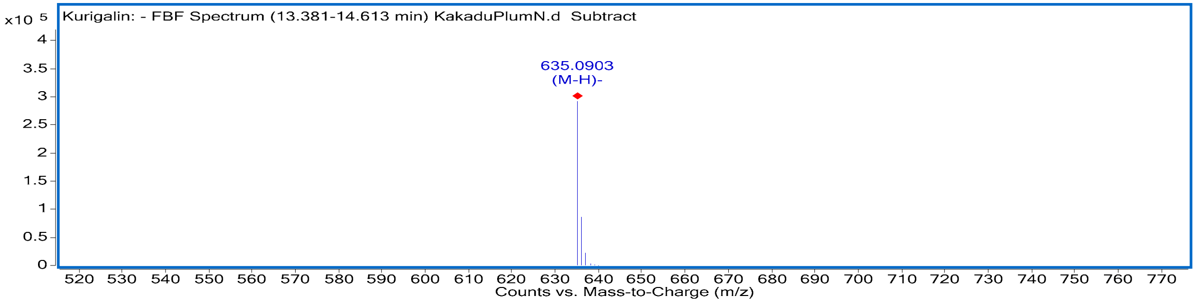
x10 5
Kurigalin: - FBF Spectrum (13.381-14.613 min) KakaduPlumN.d Subtract
635.0903
(M-H)-
Counts vs. Mass-to-Charge (m/z)

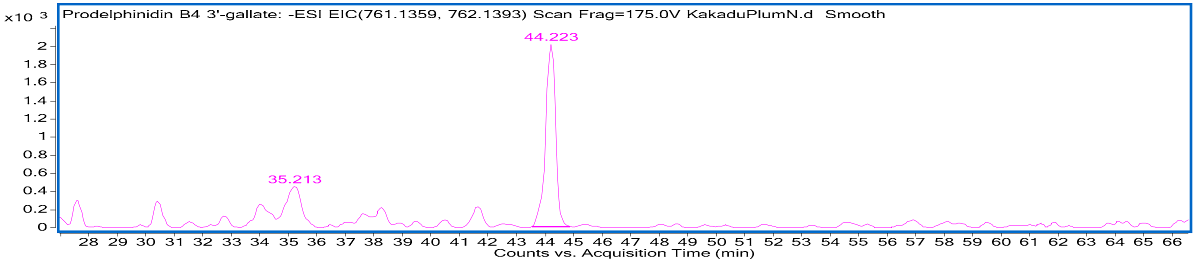
x10 3
Prodelphinidin B4 3'-gallate: -ESI EIC(761.1359, 762.1393) Scan Frag=175.0V KakaduPlumN.d Smooth
44.223
35.213
Counts vs. Acquisition Time (min)

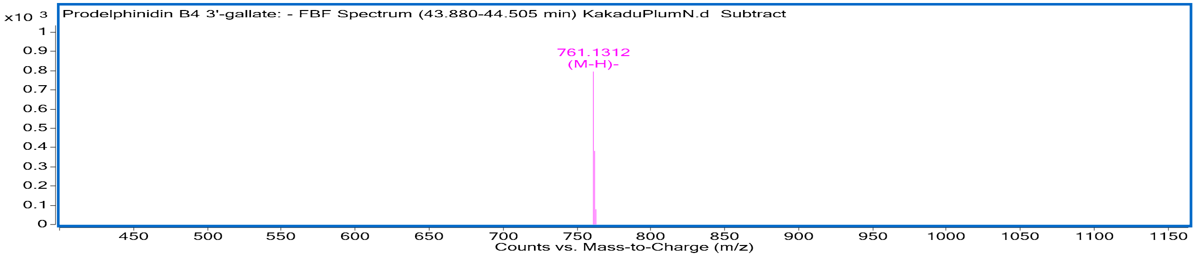
x10 3
Prodelphinidin B4 3'-gallate: - FBF Spectrum (43.880-44.505 min) KakaduPlumN.d Subtract
761.1312
(M-H)-
Counts vs. Mass-to-Charge (m/z)

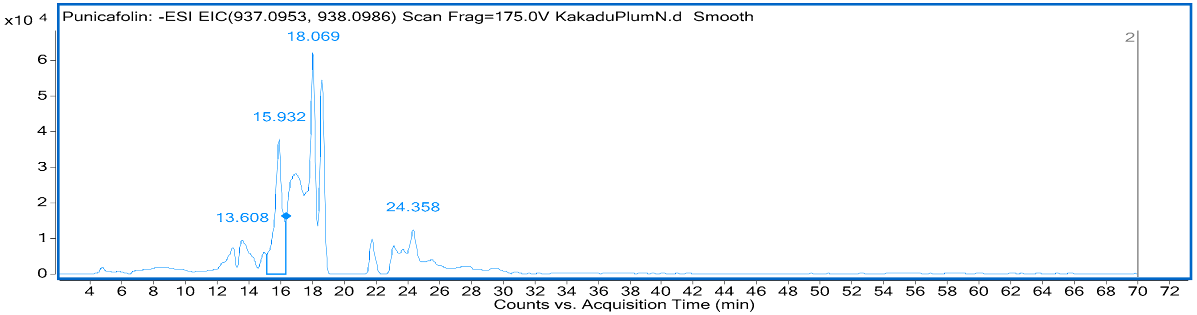
x10 4
Punicafolin: -ESI EIC(937.0953, 938.0986) Scan Frag=175.0V KakaduPlumN.d Smooth
18.069
15.932
13.608
24.358
2
Counts vs. Acquisition Time (min)

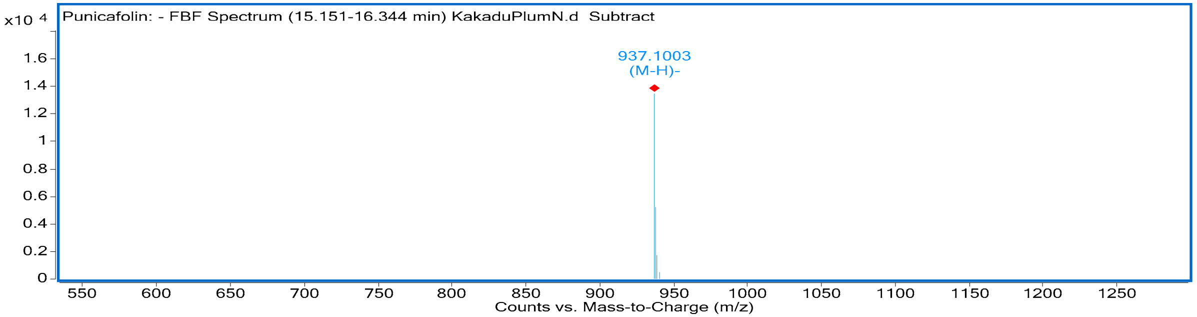
x10 4
Punicafolin: - FBF Spectrum (15.151-16.344 min) KakaduPlumN.d Subtract
937.1003
(M-H)-
Counts vs. Mass-to-Charge (m/z)

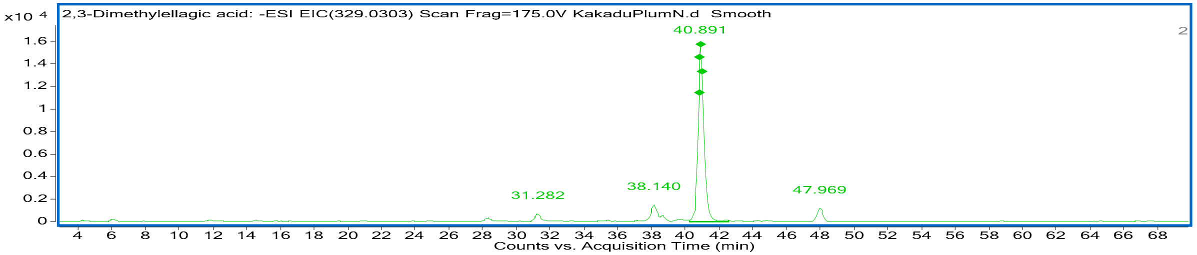
2,3-Dimethylellagic acid: -ESI EIC(329.0303) Scan Frag=175.0V KakaduPlumN.d Smooth
x10 4
40.891
31.282
38.140
47.969
Counts vs. Acquisition Time (min)

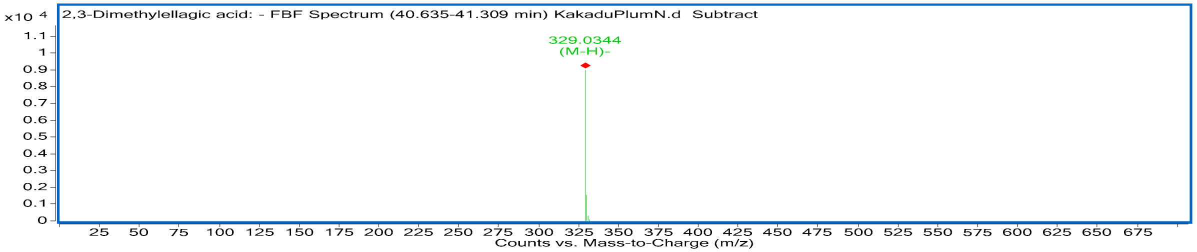
2,3-Dimethylellagic acid: - FBF Spectrum (40.635-41.309 min) KakaduPlumN.d Subtract
x10 4
329.0344
(M-H)-
Counts vs. Mass-to-Charge (m/z)

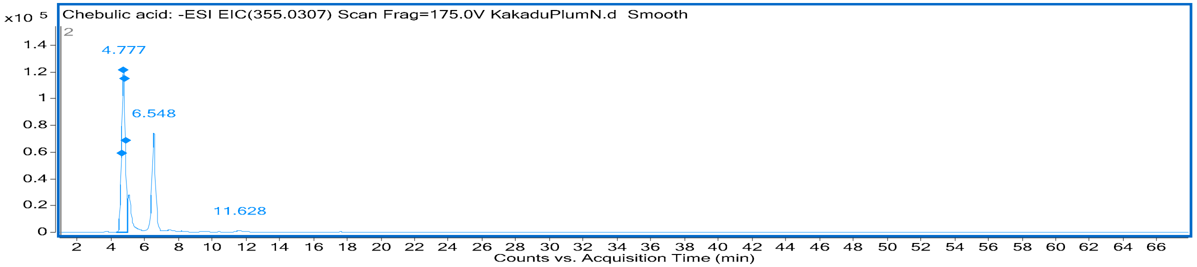
Chebulic acid: -ESI EIC(355.0307) Scan Frag=175.0V KakaduPlumN.d Smooth
x10 5
4.777
6.548
11.628
Counts vs. Acquisition Time (min)

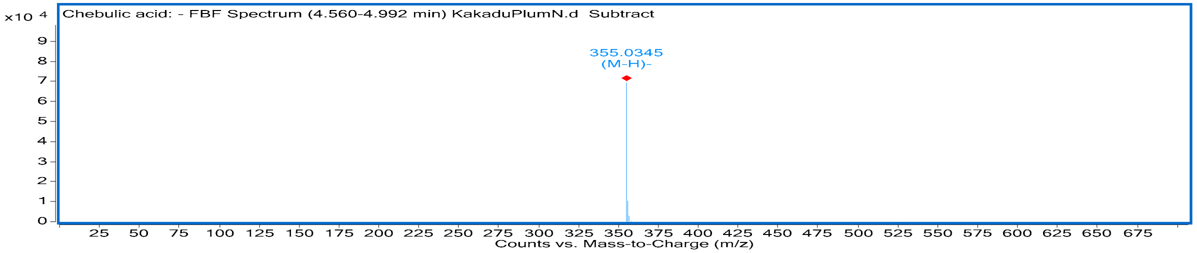
Chebulic acid: - FBF Spectrum (4.560-4.992 min) KakaduPlumN.d Subtract
x10 4
355.0345
(M-H)-
Counts vs. Mass-to-Charge (m/z)

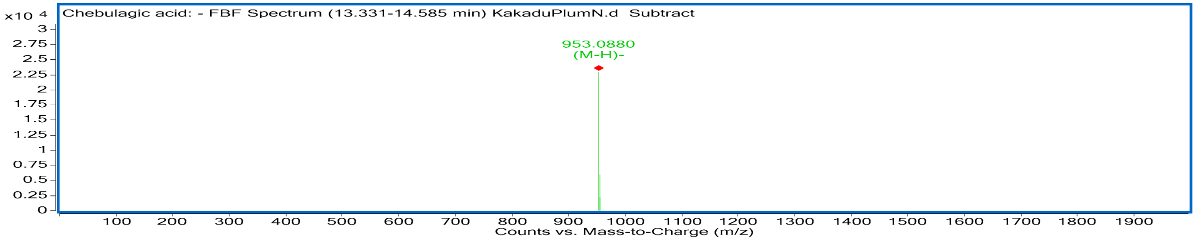
Chebulagic acid: - FBF Spectrum (13.331-14.585 min) KakaduPlumN.d Subtract
x10 4
953.0880
(M-H)-
Counts vs. Mass-to-Charge (m/z)

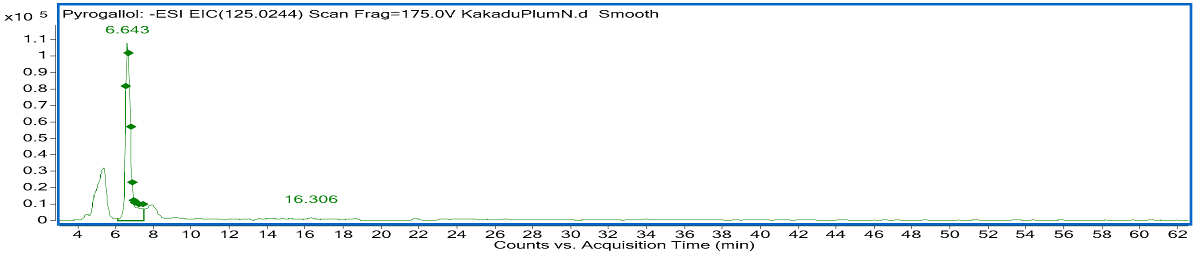
Pyrogallol: -ESI EIC(125.0244) Scan Frag=175.0V KakaduPlumN.d Smooth
x10 5
6.643
16.306
Counts vs. Acquisition Time (min)

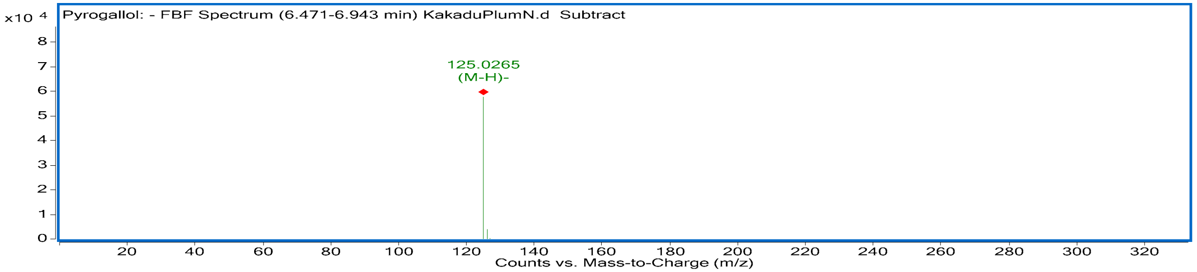
Pyrogallol: - FBF Spectrum (6.471-6.943 min) KakaduPlumN.d Subtract
x10 4
125.0265
(M-H)-
Counts vs. Mass-to-Charge (m/z)

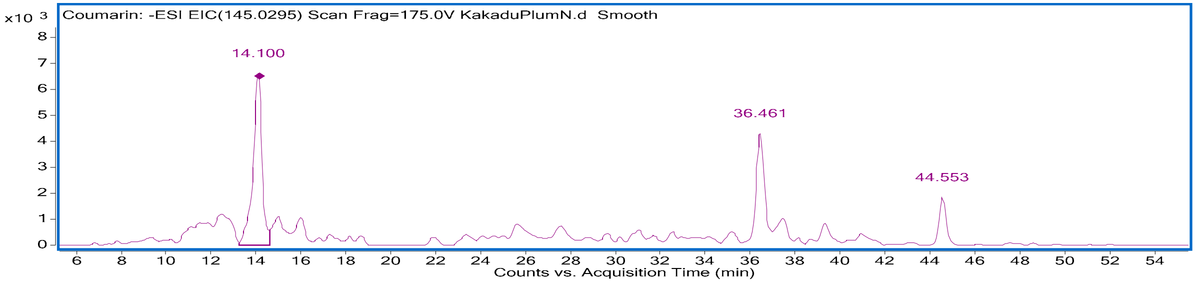
Coumarin: -ESI EIC(145.0295) Scan Frag=175.0V KakaduPlumN.d Smooth
x10 3
14.100
36.461
44.553
Counts vs. Acquisition Time (min)

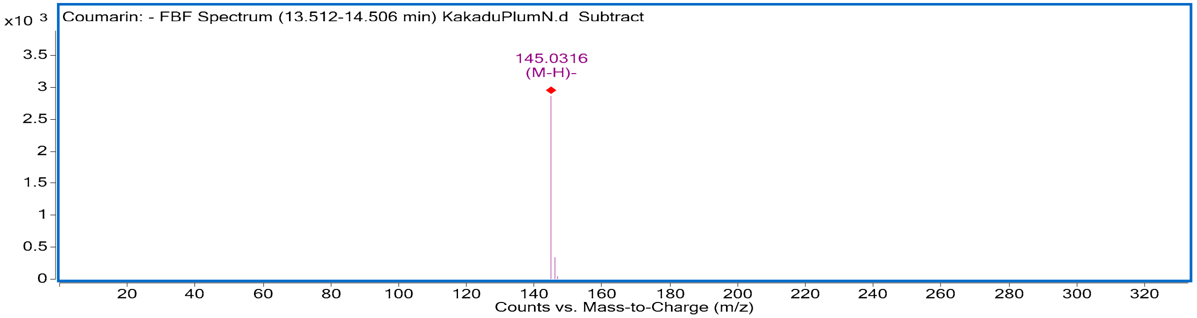
Coumarin: - FBF Spectrum (13.512-14.506 min) KakaduPlumN.d Subtract
x10 3
145.0316
(M-H)-
Counts vs. Mass-to-Charge (m/z)

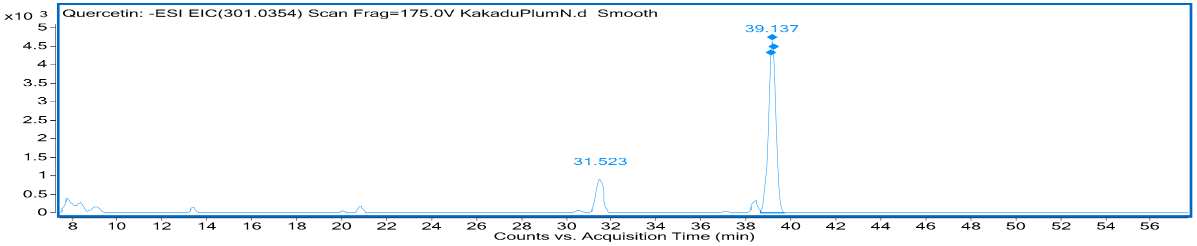
Quercetin: -ESI EIC(301.0354) Scan Frag=175.0V KakaduPlumN.d Smooth
x10 3
39.137
31.523
Counts vs. Acquisition Time (min)

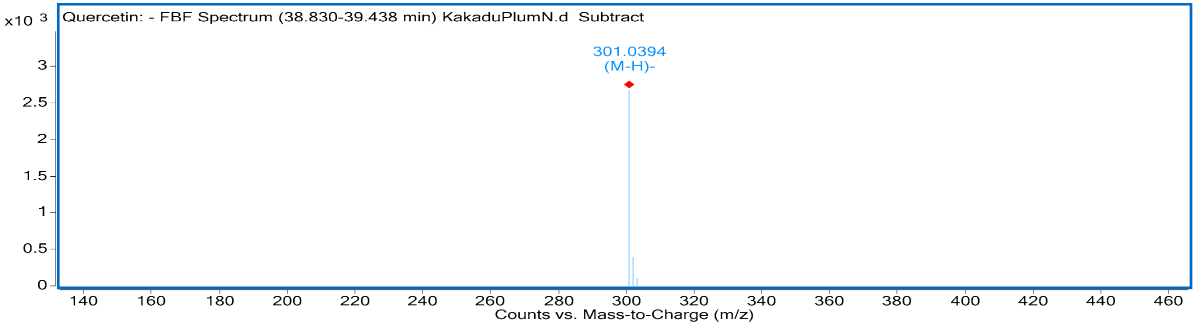
Quercetin: - FBF Spectrum (38.830-39.438 min) KakaduPlumN.d Subtract
x10 3
301.0394
(M-H)-
Counts vs. Mass-to-Charge (m/z)

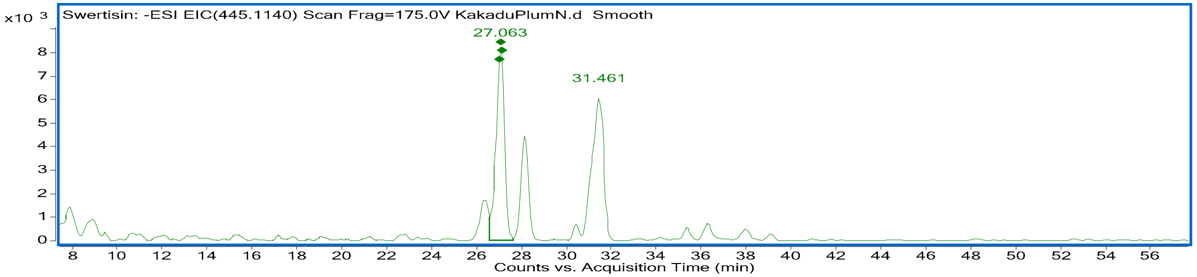
x10 3
Swertisin: -ESI EIC(445.1140) Scan Frag=175.0V KakaduPlumN.d Smooth
27.063
31.461
Counts vs. Acquisition Time (min)

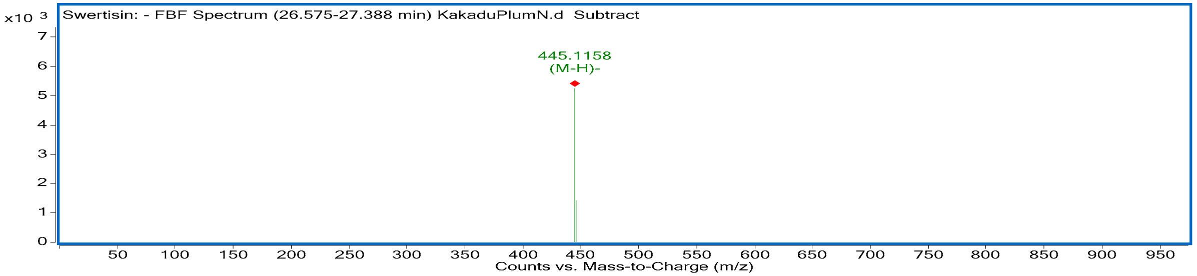
x10 3
Swertisin: - FBF Spectrum (26.575-27.388 min) KakaduPlumN.d Subtract
445.1158
(M-H)-
Counts vs. Mass-to-Charge (m/z)

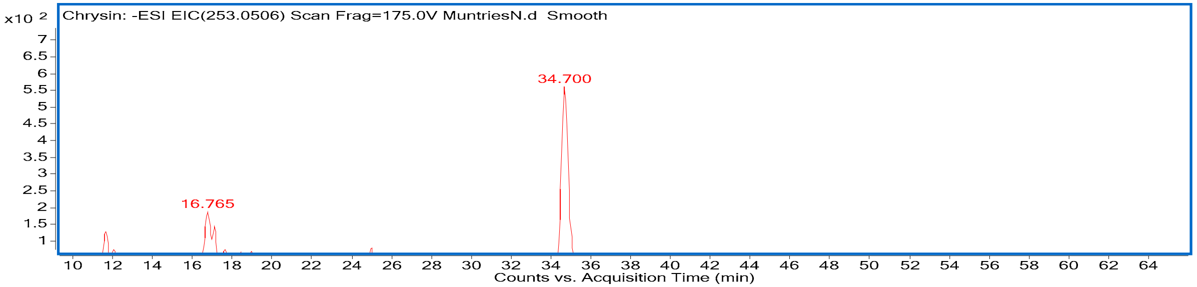
x10 2
Chrysin: -ESI EIC(253.0506) Scan Frag=175.0V MuntriesN.d Smooth
34.700
16.765
Counts vs. Acquisition Time (min)

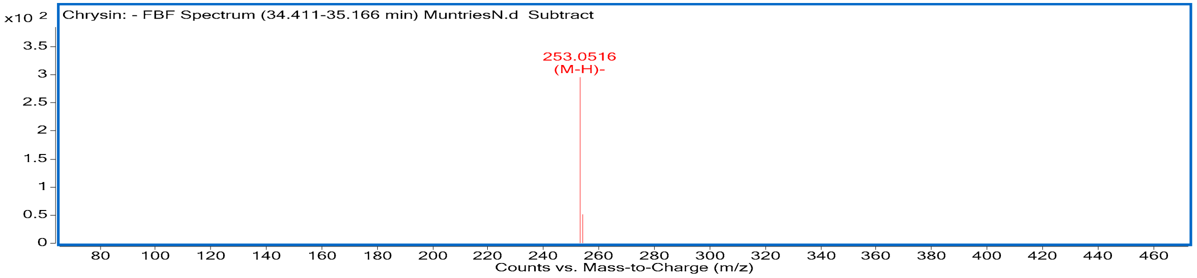
x10 2
Chrysin: - FBF Spectrum (34.411-35.166 min) MuntriesN.d Subtract
253.0516
(M-H)-
Counts vs. Mass-to-Charge (m/z)

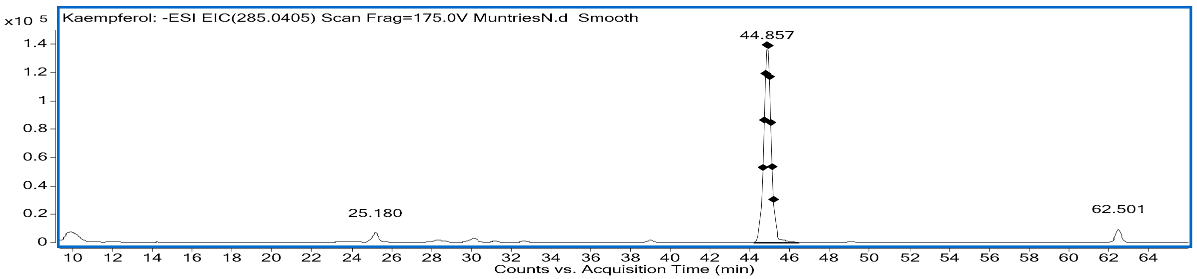
Kaempferol: -ESI EIC(285.0405) Scan Frag=175.0V MuntriesN.d Smooth
x10 5
44.857
25.180
62.501
Counts vs. Acquisition Time (min)

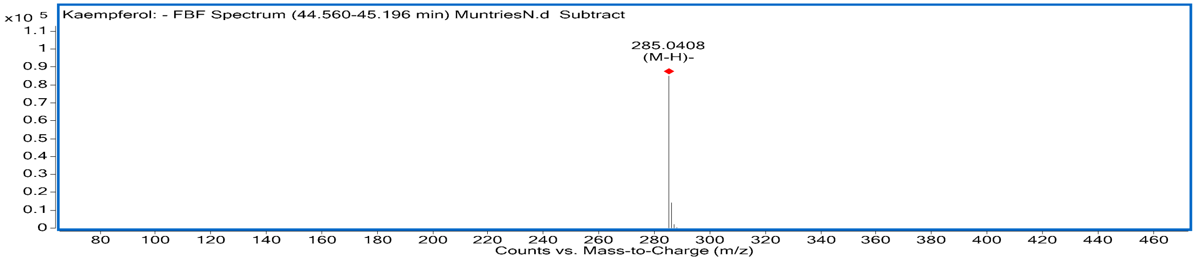
Kaempferol: - FBF Spectrum (44.560-45.196 min) MuntriesN.d Subtract
x10 5
285.0408
(M-H)-
Counts vs. Mass-to-Charge (m/z)

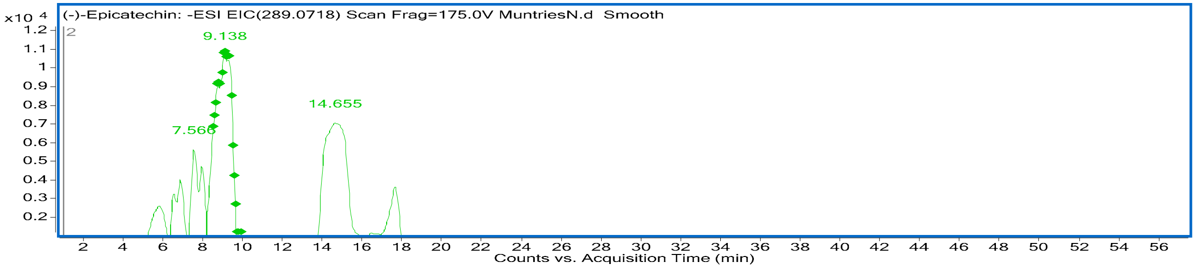
(-)-Epicatechin: -ESI EIC(289.0718) Scan Frag=175.0V MuntriesN.d Smooth
x10 4
9.138
14.655
7.566
Counts vs. Acquisition Time (min)

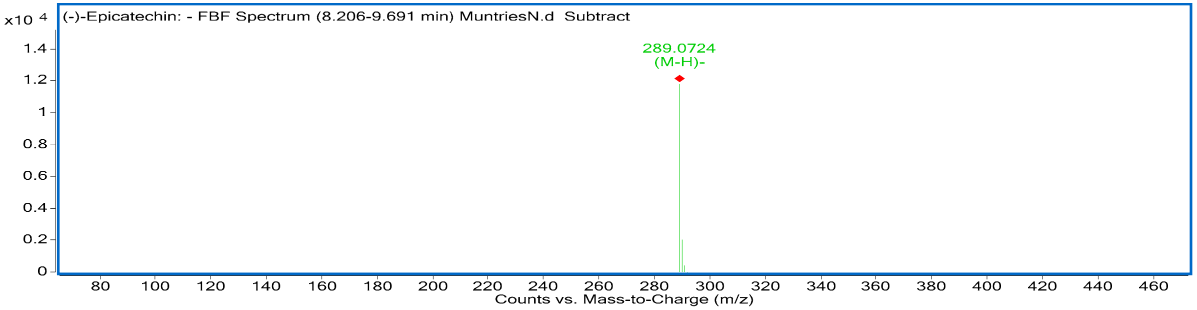
(-)-Epicatechin: - FBF Spectrum (8.206-9.691 min) MuntriesN.d Subtract
x10 4
289.0724
(M-H)-
Counts vs. Mass-to-Charge (m/z)

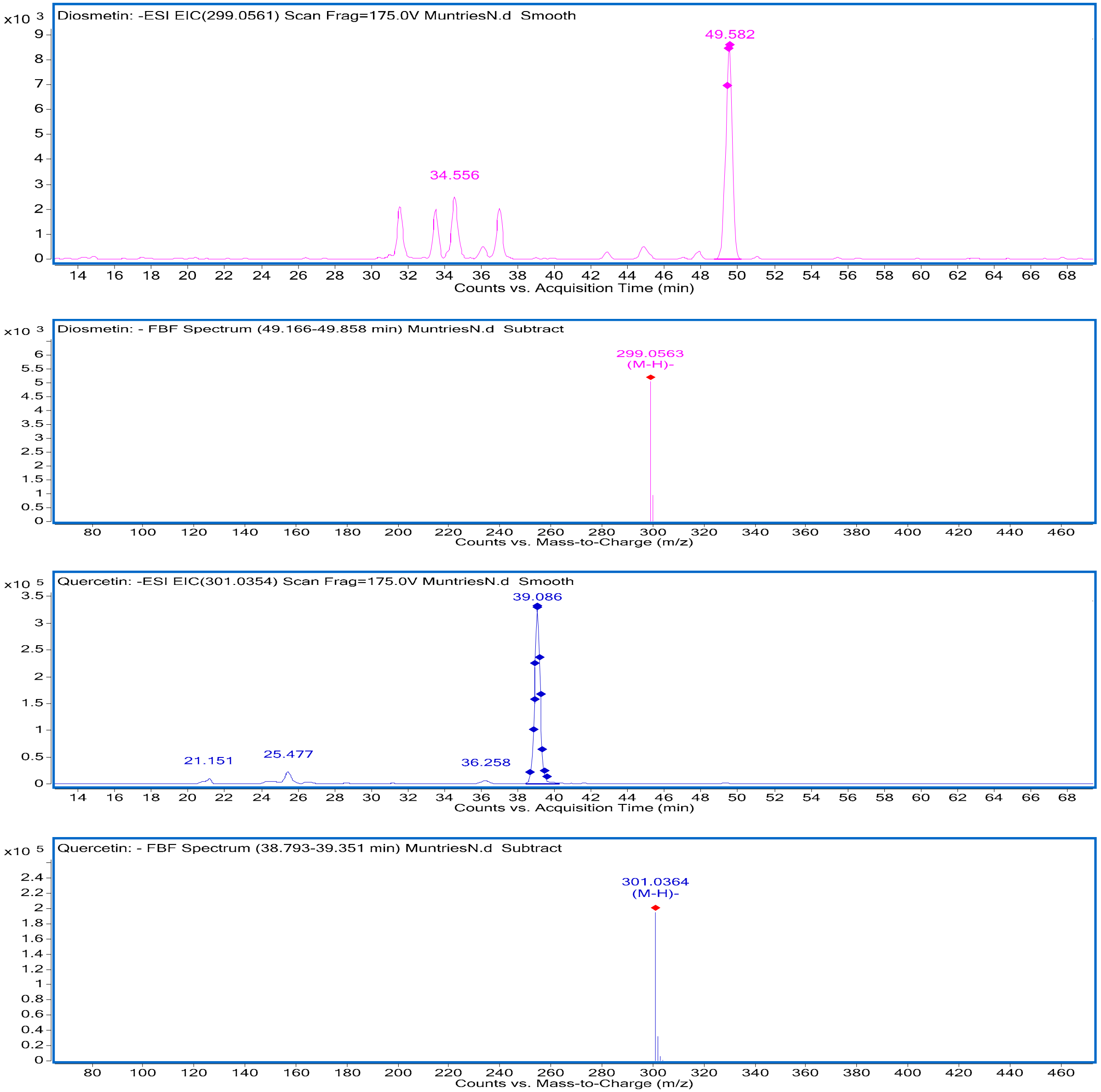

Diosmetin: -ESI EIC(299.0561) Scan Frag=175.0V MuntriesN.d Smooth
x10 3
49.582
34.556
Counts vs. Acquisition Time (min)
Diosmetin: - FBF Spectrum (49.166-49.858 min) MuntriesN.d Subtract
x10 3
299.0563
(M-H)-
Counts vs. Mass-to-Charge (m/z)
Quercetin: -ESI EIC(301.0354) Scan Frag=175.0V MuntriesN.d Smooth
x10 5
39.086
21.151
25.477
36.258
Counts vs. Acquisition Time (min)
Quercetin: - FBF Spectrum (38.793-39.351 min) MuntriesN.d Subtract
x10 5
301.0364
(M-H)-
Counts vs. Mass-to-Charge (m/z)

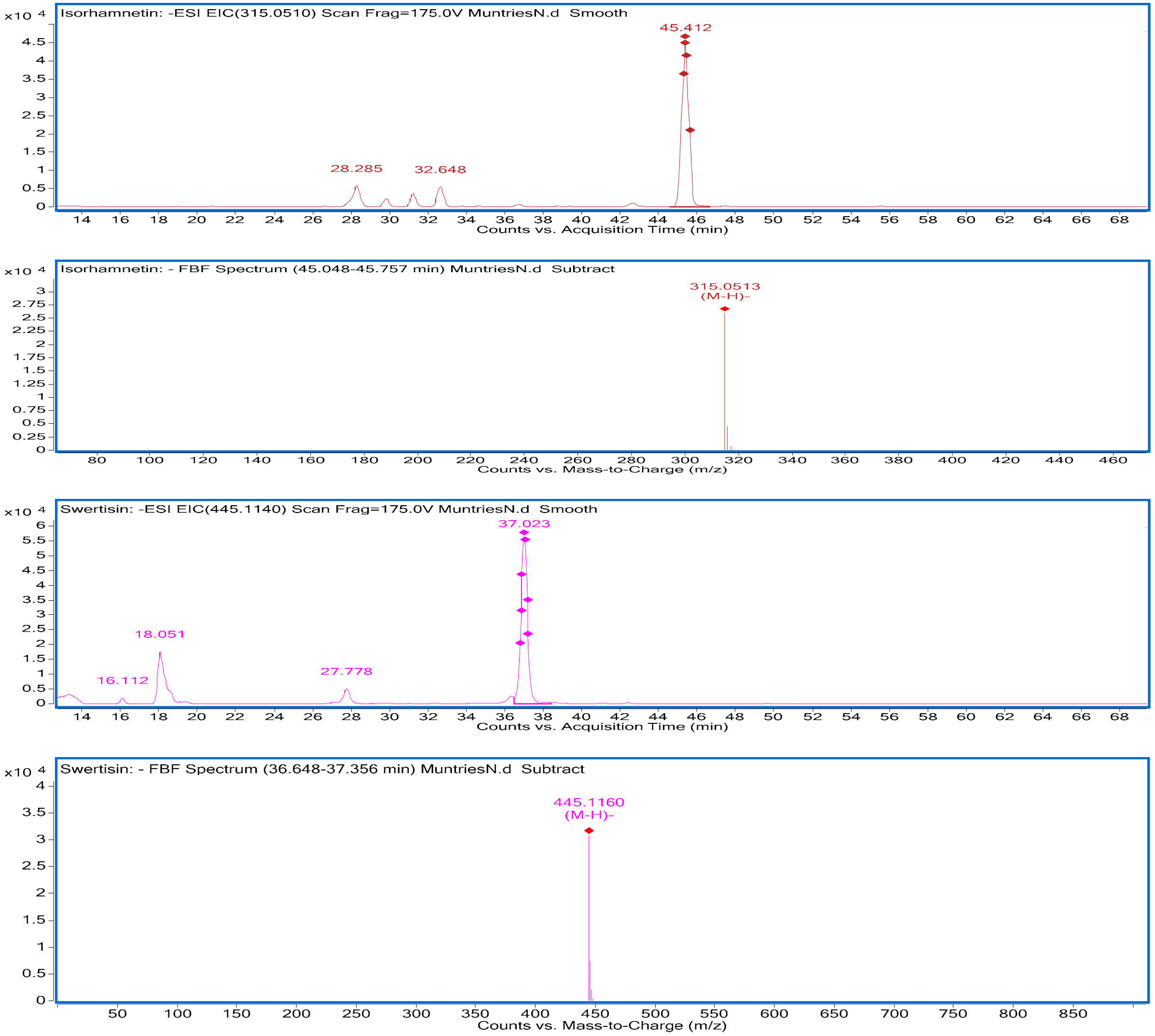

Figure S1. Chromatograms and mass spectra of some selected compounds

Table S1. Quantification of phenolic contents and antioxidant activities of native Australian fruits

Variables	TPC (μg GAE/mg)	TFC (μg QE/mg)	ABTS (μg AAE/mg)	DPPH (μg AAE/mg)	•OH-RSA (μg AAE/mg)	FICA (μg EDTA/mg)
KP	793.89 ± 22.27 [a]	491.21 ± 20.24 [a]	404.74 ± 24.61 [a]	387.31 ± 20.49 [a]	472.47 ± 9.45 [a]	244.82 ± 25.97 [a]
QP	728.53 ± 6.57 [b]	332.32 ± 5.32 [c]	353.79 ± 7.35 [c]	290.89 ± 16.44 [c]	432.47 ± 4.16 [b]	162.79 ± 5.02 [c]
DP	649.83 ± 15.75 [c]	349.45 ± 4.12 [b]	378.91 ± 7.28 [b]	309.76 ± 11.95 [b]	393.13 ± 6.11 [c]	179.91 ± 10.39 [b]
Mu	614.44 ± 31.80 [d]	173.17 ± 41.42 [d]	241.59 ± 7.38 [d]	232.95 ± 27.51 [d]	233.23 ± 5.51 [d]	150.78 ± 4.53 [d]

TPC; total phenolic content, TFC; total flavonoid content, GAE; gallic acid equivalent, QE; quercetin equivalent, Values are mean ± standard deviation in triplicate (n = 3) per milligram of freeze-dried extract. KP; Kakadu plum, QP; Quandong peach, DP; Davidson plum and Mu; Muntries. Values with the letters (a-d) are significantly different from each other ($p < 0.05$).

Table S2. Phytochemical screening of native Australian fruits through LC-ESI-QTOF-MS

**No.**	**Proposed compounds**	**Molecular Formula**	**RT (min)**	**Mode of ionization**	**Theoretical (*m/z*)**	**Observed (*m/z*)**	**Mass Error (ppm)**	**Samples**
	Phenolic acids							
	Hydroxybenzoic acid							
1	*p*-Aminobenzoic acid	$C_7H_7NO_2$	4.451	$[M+H]^+$	138.0550	138.0546	−2.9	Mu, QP
2	Gallic acid	$C_7H_6O_5$	6.329	$[M-H]^-$	169.0142	169.0153	6.5	Mu, KP
3	Protocatechuic acid	$C_7H_6O_4$	6.906	** $[M-H]^-$	153.0193	153.0195	1.3	Mu, QP, KP
4	Syringic acid	$C_9H_{10}O_5$	7.982	$[M-H]^-$	197.0455	197.0456	0.5	KP, Mu
5	Uralenneoside	$C_{12}H_{14}O_8$	9.288	** $[M-H]^-$	285.0616	285.0617	0.4	Mu, QP, KP
6	Gallic acid 4-*O*-glucoside	$C_{13}H_{16}O_{10}$	11.242	$[M-H]^-$	331.0671	331.0670	−0.3	Mu, DP
7	Protocatechuic acid 4-*O*-glucoside	$C_{13}H_{16}O_9$	12.577	** $[M-H]^-$	315.0721	315.0720	−0.3	Mu, QP, KP, DP
8	3-*O*-Methylgallic acid	$C_8H_8O_5$	13.989	[M−H]−	183.0299	183.0301	1.1	Mu, KP
9	Punicalin	$C_{34}H_{22}O_{22}$	14.517	** $[M+H]^+$	783.0676	783.0707	4.0	KP, DP

10	Ellagic acid	$C_{14}H_6O_8$	14.613	** $[M-H]^-$	300.9990	301.0002	4.0	KP, QP, Mu, DP
11	Valoneic acid dilactone	$C_{21}H_{10}O_{13}$	21.146	** $[M-H]^-$	469.0048	469.0050	0.4	KP
12	Punicalagin	$C_{48}H_{28}O_{30}$	21.148	$[M-H]^-$	1083.0592	1083.0399	−17.8	DP
13	Ellagic acid glucoside	$C_{20}H_{16}O_{13}$	24.356	** $[M-H]^-$	463.0518	463.0500	−3.9	DP, KP
14	*p*-Hydroxybenzoic acid	$C_7H_6O_3$	25.033	$[M+H]^+$	139.0390	139.0390	0.0	QP, Mu, DP, KP
15	Ellagic acid acetyl-arabinoside	$C_{21}H_{16}O_{13}$	38.185	** $[M-H]^-$	475.0518	475.0522	0.8	KP
16	Benzoic acid	$C_7H_6O_2$	39.049	$[M-H]^-$	121.0295	121.0294	−0.8	Mu, KP
17	Paeoniflorin	$C_{23}H_{28}O_{11}$	39.892	$[M-H]^-$	479.1559	479.1563	0.8	KP, Mu
	Hydroxycinnamic acids							
18	Hydroxycaffeic acid	$C_9H_8O_5$	4.217	$[M-H]^-$	195.0299	195.0309	5.1	KP
19	Caffeoyl tartaric acid	$C_{13}H_{12}O_9$	4.326	** $[M-H]^-$	311.0408	311.0413	1.6	DP, KP
20	Feruloyl tartaric acid	$C_{14}H_{14}O_9$	5.537	[M−H]−	325.0565	325.0555	−3.1	Mu, DP, KP
21	Rosmarinic acid	$C_{18}H_{16}O_8$	13.594	[M−H]−	359.0772	359.0763	−2.5	KP, QP
22	*p*-Coumaroyl glucose	$C_{15}H_{18}O_8$	15.017	** $[M-H]^-$	325.0929	325.0936	2.2	KP
23	Ferulic acid 4-*O*-glucoside	$C_{16}H_{20}O_9$	15.184	[M−H]−	355.1034	355.1045	3.1	KP
24	1-*O*-Sinapoyl-β-D-glucose	$C_{17}H_{22}O_{10}$	15.184	[M−H]−	385.1140	385.1137	−0.8	KP, DP
25	1,5-Diferuloylquinic acid	$C_{27}H_{28}O_{12}$	15.716	** [M−H]−	543.1508	543.1508	0.0	KP
26	3,5-Dicaffeoylquinic acid	$C_{25}H_{24}O_{12}$	15.734	** [M−H]−	515.1195	515.1201	1.2	DP, KP
27	Dihydroferulic acid	$C_{10}H_{12}O_4$	15.919	** [M+H]+	197.0809	197.0809	0.0	KP
28	Caffeic acid	$C_9H_8O_4$	15.956	[M−H]−	179.0350	179.0351	0.6	DP, KP
29	1,2,2'-Triferuloylgentiobiose	$C_{42}H_{46}O_{20}$	16.088	[M+H]+	871.2655	871.2636	−2.2	QP, Mu
30	Caffeic acid 3-*O*-glucuronide	$C_{15}H_{16}O_{10}$	17.213	** [M−H]−	355.0671	355.0664	−2.0	DP, KP
31	p-Coumaroyl tartaric acid	$C_{13}H_{12}O_8$	17.788	[M+H]+	297.0605	297.0616	3.7	Mu, KP, QP, DP
32	*p*-Coumaroyl glycolic acid	$C_{11}H_{10}O_5$	19.473	** [M−H]−	221.0455	221.0460	2.3	DP, KP
33	Chicoric acid	$C_{22}H_{18}O_{12}$	19.926	[M+H]+	475.0871	475.0865	−1.3	QP, Mu, KP
34	3-Sinapoylquinic acid	$C_{18}H_{22}O_{10}$	22.267	** [M−H]−	397.1140	397.1139	−0.3	KP
35	Avenanthramide K	$C_{16}H_{13}NO_6$	22.862	[M+H]+	316.0816	316.0807	−2.8	KP
36	Ferulic acid	$C_{10}H_{10}O_4$	24.003	[M−H]−	193.0506	193.0509	1.6	KP, DP
37	5-5'-Dehydrodiferulic acid	$C_{20}H_{18}O_8$	24.318	** [M−H]−	385.0929	385.0918	−2.9	QP
38	3-Caffeoylquinic acid	$C_{16}H_{18}O_9$	24.472	[M−H]−	353.0878	353.0897	5.4	QP, Mu, KP
39	*p*-Coumaric acid	$C_9H_8O_3$	24.824	** [M−H]−	163.0400	163.0411	6.7	QP, KP, Mu, DP
40	Caffeoyl glucose	$C_{15}H_{18}O_9$	26.770	** [M+H]+	343.1024	343.1023	−0.3	KP
41	1-Feruloyl-5-caffeoylquinic acid	$C_{26}H_{26}O_{12}$	30.495	[M+H]+	531.1497	531.1482	−2.8	KP, QP
42	Verbascoside A	$C_{31}H_{40}O_{16}$	35.315	[M+H]+	669.2389	669.2440	7.6	KP
43	3-Feruloyquinic acid lactone	$C_{17}H_{18}O_8$	37.144	** [M−H]−	349.0929	349.0922	−2.0	KP
44	Cinnamic acid	$C_9H_8O_2$	38.098	** [M−H]−	147.0451	147.0441	−6.8	KP, Mu
45	3,4-Dimethoxycinnamic acid	$C_{11}H_{12}O_4$	38.603	** [M+H]+	209.0809	209.0809	0.0	KP
46	Avenanthramide 2f	$C_{17}H_{15}NO_6$	38.837	** [M+H]+	330.0972	330.0980	2.4	KP
47	1-Caffeoyl-5-feruloylquinic acid	$C_{26}H_{26}O_{12}$	38.873	** [M−H]−	529.1351	529.1353	0.4	KP
48	*p*-Coumaroyl tyrosine	$C_{18}H_{17}NO_5$	39.463	[M+H]+	328.1180	328.1191	3.4	KP, DP

49	1,2,2'-Trisinapoylgentiobiose	$C_{45}H_{52}O_{23}$	44.490	** [M+H]+	961.2972	961.2984	1.2	KP
50	Ferulic acid 4-*O*-glucuronide	$C_{16}H_{18}O_{10}$	47.520	** [M+H]+	371.0973	371.0965	−2.2	KP
51	Avenanthramide 2p	$C_{16}H_{13}NO_5$	49.013	[M+H]+	300.0867	300.0856	−3.7	KP
52	Sinapine	$C_{16}H_{24}NO_5$	56.743	** [M−H]−	309.1581	309.1597	5.2	DP
53	3-Feruloylquinic acid	$C_{17}H_{20}O_9$	66.694	** [M+H]+	369.1180	369.1173	−1.9	DP, KP
54	3-*p*-Coumaroylquinic acid	$C_{16}H_{18}O_8$	69.686	** [M−H]−	337.0929	337.0924	−1.5	QP, Mu, KP, DP
	Other phenolic acids							
55	Dihydroferulic acid 4-sulfate	$C_{10}H_{12}O_7S$	15.061	[M+H]+	277.0377	277.0411	12.3	KP
56	Dihydrocaffeic acid 3-*O*-glucuronide	$C_{15}H_{18}O_{10}$	15.211	[M−H]−	357.0827	357.0851	6.7	KP
57	5-(3',5'-Dihydroxyphenyl)-γ-valerolactone 3-*O*-glucuronide	$C_{17}H_{20}O_{10}$	15.601	** [M−H]−	383.0984	383.0980	−1.0	KP
58	Scopolamine	$C_{17}H_{21}NO_4$	16.993	** [M+H]+	304.1544	304.1544	0.0	QP, Mu
59	Homovanillic acid	$C_9H_{10}O_4$	22.536	[M+H]+	183.0652	183.0648	−2.2	KP, QP
60	4-Hydroxyphenyl-2-propionic acid	$C_9H_{10}O_3$	30.714	[M+H]+	167.0703	167.0709	3.6	KP
61	3-Phenylpropionic acid	$C_9H_{10}O_2$	31.088	[M+H]+	151.0754	151.0747	−4.6	KP
62	5-(3',4'-dihydroxyphenyl)-valeric acid	$C_{11}H_{14}O_4$	34.116	[M+H]+	211.0965	211.0967	0.9	Mu, QP, KP, DP
63	2-Hydroxy-2-phenylacetic acid	$C_8H_8O_3$	34.235	[M+H]+	153.0546	153.0541	−3.3	KP
64	3-Hydroxyphenylvaleric acid	$C_{11}H_{14}O_3$	49.582	** [M−H]−	193.0870	193.0866	−2.1	Mu, QP, KP
	Flavonoids							
	Anthocyanins							
65	Cyanidin 3-*O*-(6''-malonyl-3''-glucosyl-glucoside)	$C_{30}H_{33}O_{19}$	4.053	$[M+H]^+$	697.1616	697.1620	0.6	DP, Mu
66	Pelargonidin 3-*O*-(6''-succinyl-glucoside)	$C_{25}H_{25}O_{13}$	4.518	$[M+H]^+$	533.1295	533.1273	−4.1	DP
67	Pelargonidin 3-rhamnoside	$C_{21}H_{21}O_9$	4.591	$[M+H]^+$	417.1186	417.1178	−1.9	Mu
68	Pelargonidin 3-arabinoside	$C_{20}H_{19}O_9$	4.613	$[M+H]^+$	403.1029	403.1023	−1.5	QP
69	Cyanidin-aldodipentoside	$C_{25}H_{27}O_{14}$	4.697	$[M+H]^+$	551.1401	551.1378	−4.2	QP
70	Cyanidin 3-galactoside *p*-coumaric acid ester	$C_{30}H_{27}O_{13}$	4.732	$[M+H]^+$	595.1452	595.1467	2.5	Mu
71	Pelargonidin 3-*O*-β-D-caffeoylglucoside	$C_{30}H_{27}O_{13}$	4.780	$[M+H]^+$	595.1452	595.1475	4.0	Mu
72	Pelargonidin 3-*p*-coumarylglucoside	$C_{30}H_{27}O_{12}$	6.523	$[M+H]^+$	579.1502	579.1500	−0.3	Mu
73	Capensinidin	$C_{18}H_{17}O_7$	6.847	$[M+H]^+$	345.0974	345.0976	0.6	Mu
74	Cyanidin 3-*O*-glucoside	$C_{21}H_{21}O_{11}$	8.571	$[M+H]^+$	449.1084	449.1083	−0.2	DP, Mu, QP
75	Cyanidin 3-rutinoside	$C_{27}H_{31}O_{15}$	12.772	$[M+H]^+$	595.1663	595.1653	−1.7	QP, Mu
76	Delphinidin 3-*O*-sambubioside	$C_{26}H_{29}O_{16}$	13.584	$[M+H]^+$	597.1456	597.1456	0.0	DP, Mu
77	Cyanidin 3-rhamnoside	$C_{21}H_{21}O_{10}$	15.444	$[M+H]^+$	433.1135	433.1129	−1.4	QP, Mu, DP
78	Cyanidin 3-*O*-(6''-p-coumaroyl-glucoside)	$C_{30}H_{27}O_{13}$	15.593	$[M+H]^+$	595.1452	595.1460	1.3	DP
79	Delphinidin 3,5-diglucoside	$C_{27}H_{31}O_{17}$	15.616	$[M+H]^+$	627.1561	627.1546	−2.4	Mu
80	Cyanidin 3-(2G-glucosylrutinoside)	$C_{33}H_{41}O_{20}$	15.743	$[M+H]^+$	757.2191	757.2175	−2.1	QP
81	Malvidin 3-rutinoside	$C_{29}H_{35}O_{16}$	16.729	$[M+H]^+$	639.1925	639.1937	1.9	Mu
82	Pelargonidin 3-(2glu glucosylrutinoside)	$C_{33}H_{41}O_{19}$	17.683	$[M+H]^+$	741.2242	741.2234	−1.1	QP, Mu

83	Cyanidin 3-(3'',6''-dimalonylglucoside)	$C_{27}H_{25}O_{17}$	18.990	$[M+H]^+$	621.1092	621.1089	−0.5	Mu
84	Pelargonidin 3-*O*-rutinoside	$C_{27}H_{31}O_{14}$	19.104	$[M+H]^+$	579.1714	579.1672	−7.3	DP
85	Cyanidin 3-gentiobioside	$C_{27}H_{31}O_{16}$	19.179	$[M+H]^+$	611.1612	611.1614	0.3	QP, Mu
86	Cyanidin 3-*O*-xyloside	$C_{20}H_{19}O_{10}$	19.369	$[M+H]^+$	419.0978	419.0982	1.0	DP
87	Delphinidin	$C_{15}H_{11}O_7$	19.390	$[M+H]^+$	303.0505	303.0505	0.0	QP, Mu, DP
88	Delphinidin 3-rutinoside	$C_{27}H_{31}O_{16}$	20.500	$[M+H]^+$	611.1612	611.1614	0.3	QP, Mu
89	Petunidin 3-(6''-*p*-coumaryl-glucoside) 5-glucoside	$C_{37}H_{39}O_{19}$	20.996	$[M+H]^+$	787.2086	787.2094	1.0	Mu
90	Delphinidin 3-glucoside	$C_{21}H_{21}O_{12}$	21.185	$[M+H]^+$	465.1033	465.1031	−0.4	DP, QP, Mu
91	Cyanidin	$C_{15}H_{11}O_6$	22.486	$[M+H]^+$	287.0555	287.0554	−0.3	QP, DP
92	Peonidin 3,5-diglucoside	$C_{28}H_{33}O_{16}$	22.562	$[M+H]^+$	625.1769	625.1783	2.2	Mu
93	Apigenidin	$C_{15}H_{11}O_4$	22.800	$[M+H]^+$	255.0657	255.0654	−1.2	QP, Mu
94	Pelargonidin	$C_{15}H_{11}O_5$	23.040	$[M+H]^+$	271.0606	271.0607	0.4	DP, Mu, QP
95	Delphinidin 3-(6''-malonyl-glucoside)	$C_{24}H_{23}O_{15}$	23.304	$[M+H]^+$	551.1037	551.1039	0.4	Mu
96	Cyanidin 7-(3-glucosyl-6-malonylglucoside) 4'-glucoside	$C_{38}H_{47}O_{22}$	23.411	$[M+H]^+$	855.2559	855.2570	1.3	QP
97	Cyanidin 3-(2G-xylosylrutinoside)	$C_{32}H_{39}O_{19}$	23.571	$[M+H]^+$	728.2164	728.2160	−0.5	Mu
98	Delphinidin 3-xyloside	$C_{20}H_{19}O_{11}$	24.070	$[M+H]^+$	435.0927	435.0929	0.5	Mu, QP
99	Malvidin 3-*O*-arabinoside	$C_{22}H_{23}O_{11}$	25.043	$[M+H]^+$	463.1240	463.1252	2.6	DP
100	Cyanidin 3-(6''-p-coumarylsophoroside) 5-glucoside	$C_{42}H_{47}O_{23}$	26.393	$[M+H]^+$	919.2508	919.2490	−2.0	QP
101	Petunidin 3-glucoside	$C_{22}H_{23}O_{12}$	26.516	$[M+H]^+$	479.1190	479.1173	−3.5	Mu
102	Delphinidin 3-*O*-arabinoside	$C_{20}H_{19}O_{11}$	26.907	$[M+H]^+$	435.0927	435.0927	0.0	DP
103	Cyanidin 3-arabinoside	$C_{20}H_{19}O_{10}$	27.260	$[M+H]^+$	419.0978	419.0986	1.9	Mu
104	Delphinidin 3-*O*-glucosyl-glucoside	$C_{27}H_{31}O_{17}$	27.966	$[M+H]^+$	627.1561	627.1590	4.6	DP
105	Malvidin-3-galactoside	$C_{23}H_{25}O_{12}$	28.152	$[M+H]^+$	493.1346	493.1349	0.6	Mu
106	Pelargonidin 3-*O*-glucoside	$C_{21}H_{21}O_{10}$	28.271	$[M+H]^+$	433.1135	433.1118	−3.9	DP, QP, Mu
107	Petunidin 3-*O*-(6''-p-coumaroyl-glucoside)	$C_{31}H_{29}O_{14}$	28.375	$[M+H]^+$	625.1557	625.1549	−1.3	DP
108	Cyanidin 3-*O*-(6''-malonyl-glucoside)	$C_{24}H_{23}O_{14}$	28.495	$[M+H]^+$	535.1088	535.1091	0.6	DP
109	Pelargonidin 3-*O*-[b-D-Xylopyranosyl-(1->2)-a-D-glucopyranoside]	$C_{26}H_{28}O_{14}$	28.580	$[M+H]^+$	565.1557	565.1540	−3.0	Mu
110	Peonidin 3-rhamnoside 5-glucoside	$C_{28}H_{33}O_{15}$	29.709	$[M+H]^+$	609.1819	609.1796	−3.8	Mu
111	Delphinidin 3-(6''-acetylgalactoside)	$C_{23}H_{23}O_{13}$	30.743	$[M+H]^+$	507.1139	507.1121	−3.5	Mu
112	Cyanidin 3-*O*-arabinoside	$C_{20}H_{19}O_{10}$	30.969	$[M+H]^+$	419.0978	419.0972	−1.4	DP
113	Malvidin 3-arabinoside	$C_{22}H_{23}O_{11}$	31.385	$[M+H]^+$	463.1240	463.1223	−3.7	Mu
114	Malvidin 3-(6''-acetyl-galactoside)	$C_{25}H_{27}O_{13}$	31.434	$[M+H]^+$	535.1452	535.1441	−2.1	Mu
115	Diosmetinidin	$C_{16}H_{13}O_5$	31.660	$[M+H]^+$	285.0763	285.0753	−3.5	QP, Mu
116	Malvidin	$C_{17}H_{15}O_7$	31.832	$[M+H]^+$	331.0818	331.0808	−3.0	Mu
117	Malvidin-3-*O*-glucoside	$C_{23}H_{25}O_{12}$	31.852	$[M+H]^+$	493.1346	493.1341	−1.0	Mu, QP

118	Petunidin	$C_{16}H_{13}O_7$	32.507	$[M+H]^+$	317.0661	317.0653	−2.5	Mu, QP
119	Petunidin 3-rhamnoside	$C_{22}H_{23}O_{10}$	33.414	$[M+H]^+$	447.1291	447.1274	−3.8	Mu
120	Pelargonidin 3-rutinoside	$C_{27}H_{30}O_{14}$	34.439	$[M+H]^+$	579.1714	579.1695	−3.3	Mu
121	Peonidin	$C_{16}H_{13}O_6$	34.452	$[M+H]^+$	301.0712	301.0706	−2.0	Mu, DP
122	Cyanidin 3-sambubioside	$C_{26}H_{29}O_{15}$	34.816	$[M+H]^+$	581.1506	581.1521	2.6	DP, Mu
123	Rosinidin	$C_{17}H_{15}O_6$	36.287	$[M+H]^+$	315.0868	315.0868	0.0	QP, Mu
124	Delphinidin 3-*O*-(6''-*p*-coumaroyl-glucoside)	$C_{30}H_{27}O_{14}$	45.856	$[M+H]^+$	611.1401	611.1410	1.5	DP
125	Malvidin 3-*O*-(6''-caffeoyl-glucoside)	$C_{32}H_{31}O_{15}$	49.013	$[M+H]^+$	655.1663	655.1655	−1.2	DP
	Flavanols							
127	(-)-Epigallocatechin 3-*O*-gallate	$C_{22}H_{18}O_{11}$	5.047	[M−H]−	457.0776	457.0769	−1.5	DP, KP
128	(-)-Epicatechin	$C_{15}H_{14}O_6$	9.138	** [M−H]−	289.0717	289.0719	0.7	Mu, KP
129	Theaflavin 3,3'-*O*-digallate	$C_{43}H_{32}O_{20}$	13.218	[M−H]−	867.1414	867.1396	−2.1	DP
130	(-)-Epigallocatechin 7-*O*-glucuronide	$C_{21}H_{22}O_{13}$	14.240	** [M−H]−	481.0987	481.0982	−1.0	KP
131	(+)-Gallocatechin	$C_{15}H_{14}O_7$	14.849	** [M−H]−	305.0667	305.0653	−4.6	KP, Mu
132	Prodelphinidin dimer B3	$C_{30}H_{26}O_{14}$	18.827	** [M−H]−	609.1250	609.1212	−6.2	QP, KP, DP
133	4''-*O*-Methylepigallocatechin 3-*O*-gallate	$C_{23}H_{20}O_{11}$	22.766	** [M−H]−	471.0933	471.0924	−1.9	KP
134	Elephantorrhizol	$C_{15}H_{14}O_8$	23.516	** [M−H]−	321.0616	321.0612	−1.2	KP, QP
135	4'-*O*-Methylepigallocatechin	$C_{16}H_{16}O_7$	23.730	[M+H]+	321.0969	321.0965	−1.2	QP, Mu, KP
136	4'-*O*-Methyl-(-)-epigallocatechin 7-*O*-glucuronide	$C_{22}H_{24}O_{13}$	23.851	[M−H]−	495.1144	495.1147	0.6	KP
137	Dryopteric acid	$C_{17}H_{16}O_8$	25.633	[M−H]−	347.0772	347.0766	−1.7	KP, QP, Mu
138	4',4''-Dimethylepigallocatechin gallate	$C_{24}H_{22}O_{11}$	40.399	[M−H]−	485.1089	485.1066	−4.7	Mu
	Flavonols							
139	Kaempferide	$C_{16}H_{11}O_6$	4.242	[M−H]−	298.0483	298.0461	−7.4	DP
140	5,4'-Dihydroxy-3,3'-dimethoxy-6:7-methylenedioxyflavone 4'-*O*-glucuronide	$C_{24}H_{22}O_{14}$	4.296	** [M−H]−	533.0937	533.0931	−1.1	KP
141	Kaempferol 3-*O*-glucoside	$C_{21}H_{20}O_{11}$	6.224	[M−H]−	447.0933	447.0933	0.0	Mu, QP, KP
142	Taxifolin 4',7-diglucoside	$C_{27}H_{32}O_{17}$	9.484	[M−H]−	627.1567	627.1571	0.6	Mu, KP
143	3-Methoxynobiletin	$C_{22}H_{24}O_9$	12.919	[M−H]−	431.1347	431.1357	2.3	QP, KP
144	Quercetin 3-*O*-xylosyl-rutinoside	$C_{32}H_{38}O_{20}$	15.480	[M−H]−	741.1883	741.1875	−1.1	KP
145	Myricetin 3-*O*-rutinoside	$C_{27}H_{30}O_{17}$	15.616	** [M+H]+	627.1556	627.1546	−1.6	Mu, QP
146	Kaempferol 3-*O*-glucosyl-rhamnosyl-galactoside	$C_{33}H_{40}O_{20}$	15.743	[M+H]+	757.2186	757.2175	−1.5	QP, Mu
147	Kaempferol 3-*O*-(2''-rhamnosyl-galactoside) 7-*O*-rhamnoside	$C_{33}H_{40}O_{19}$	17.683	** [M+H]+	741.2237	741.2234	−0.4	QP, Mu, KP
148	Quercetin 3-rutinoside (Rutin)	$C_{27}H_{30}O_{16}$	19.684	[M−H]−	609.1461	609.1467	1.0	KP, QP, Mu, DP
149	Kaempferol 3-*O*-xylosyl-glucoside	$C_{26}H_{28}O_{15}$	20.245	** [M−H]−	579.1355	579.1364	1.6	DP, KP
150	Myricetin	$C_{15}H_{10}O_8$	20.505	** [M−H]−	317.0303	317.0310	2.2	Mu, DP, KP
151	Myricetin 3-*O*-galactoside	$C_{21}H_{20}O_{13}$	20.690	[M−H]−	479.0831	479.0844	2.7	QP, DP, Mu

152	Dihydromyricetin	$C_{15}H_{12}O_8$	21.124	[M−H]−	319.0459	319.0457	−0.6	QP
153	Luteolin 7-glucoside	$C_{21}H_{20}O_{11}$	21.134	[M−H]−	447.0933	447.0933	0.0	Mu
154	Quercetin	$C_{15}H_{10}O_7$	21.138	[M+H]+	303.0499	303.0497	−0.7	Mu, QP, KP
155	3'-*O*-Methylmyricetin (Annulatin)	$C_{16}H_{12}O_8$	21.489	[M+H]+	333.0605	333.0602	−0.9	Mu, KP
156	Quercetin 3-O-xylosyl-glucuronide	$C_{26}H_{26}O_{17}$	22.214	** [M+H]+	611.1243	611.1236	−1.1	DP, KP, QP
157	3,5,7-Trihydroxyflavone	$C_{15}H_{10}O_5$	23.040	[M+H]+	271.0601	271.0607	2.2	KP, QP
158	Isorhamnetin 3-O-rutinoside	$C_{28}H_{32}O_{16}$	23.101	[M−H]−	623.1617	623.1613	−0.6	Mu, QP, KP
159	Quercetin 3-*O*-xyloside	$C_{20}H_{18}O_{11}$	24.070	[M+H]+	435.0922	435.0929	1.6	Mu, KP
160	Quercetin 3'-*O*-glucuronide	$C_{21}H_{18}O_{13}$	25.853	** [M−H]−	477.0674	477.0676	0.4	DP, KP
161	Myricetin 3-*O*-rhamnoside	$C_{21}H_{20}O_{12}$	26.301	** [M+H]+	465.1028	465.1032	0.9	Mu, KP, QP
162	Kaempferol 3,7,4'-*O*-triglucoside	$C_{33}H_{40}O_{21}$	26.326	[M−H]−	771.1989	771.1968	−2.7	KP, Mu, QP
163	Dihydroquercetin 3-*O*-rhamnoside	$C_{21}H_{22}O_{11}$	26.506	** [M−H]−	449.1089	449.1112	5.1	QP, KP, Mu
164	Myricetin 3-*O*-arabinoside	$C_{20}H_{18}O_{12}$	27.267	** [M−H]−	449.0725	449.0726	0.2	KP, DP
165	Jaceidin 4'-*O*-glucuronide	$C_{24}H_{24}O_{14}$	27.937	** [M−H]−	535.1093	535.1098	0.9	KP
166	Quercetin 3-*O*-(6''-malonyl)-glucoside	$C_{24}H_{22}O_{15}$	28.371	** [M−H]−	549.0886	549.0875	−2.0	KP
167	3,7-Dimethylquercetin	$C_{17}H_{14}O_7$	31.852	[M+H]+	331.0813	331.0805	−2.4	Mu
168	Isorhamnetin 4'-*O*-glucuronide	$C_{22}H_{20}O_{13}$	35.414	** [M−H]−	491.0831	491.0833	0.4	KP, DP, Mu, QP
169	Quercetin 3-*O*-glucosyl-xyloside	$C_{26}H_{28}O_{16}$	35.957	** [M+H]+	597.1450	597.1454	0.7	KP, DP
170	Europetin 3-galactoside	$C_{22}H_{22}O_{13}$	37.923	** [M−H]−	493.0987	493.1003	3.2	Mu
171	Isorhamnetin	$C_{16}H_{12}O_7$	45.412	[M−H]−	315.0510	315.0513	1.0	Mu, KP
172	Quercetin 3'-sulfate	$C_{15}H_{10}O_{10}S$	47.609	[M+H]+	383.0068	383.0080	3.1	KP
	Dihydroflavonols							
173	Dihydromyricetin	$C_{15}H_{12}O_8$	21.124	[M−H]−	319.0459	319.0457	−0.6	QP
174	Dihydroquercetin 3-*O*-rhamnoside	$C_{21}H_{22}O_{11}$	26.506	** [M−H]−	449.1089	449.1112	5.1	QP, KP, Mu
	Flavones and Isoflavones							
175	3',4',5,7-Tetrahydroxy-3,8-dimethoxyflavone	$C_{17}H_{14}O_8$	4.000	** [M+H]+	347.0762	347.0764	0.6	DP, KP
176	7-Hydroxy-5,4'-dimethoxyflavone 8-C-rhamnoside	$C_{23}H_{24}O_9$	6.214	** [M−H]−	443.1347	443.1363	3.6	QP, KP
177	Cirsilineol	$C_{18}H_{16}O_7$	6.422	[M+H]+	345.0969	345.0965	−1.2	KP, DP
178	Racemoflavone	$C_{21}H_{18}O_6$	7.377	[M−H]−	365.1030	365.1035	1.4	KP
179	6-Hydroxyluteolin 7-*O*-rhamnoside	$C_{21}H_{20}O_{11}$	8.571	** [M+H]+	449.1079	449.1083	0.9	KP
180	Apigenin	$C_{15}H_{10}O_5$	12.720	** [M+H]+	271.0601	271.0599	−0.7	QP, Mu
181	(-)-8-(2-Carboxy-1-phenylethyl)-3,5,7-trihydroxyflavone delta-lactone	$C_{24}H_{16}O_6$	13.608	[M−H]−	399.0874	399.0868	−1.5	KP
182	3,4',7-Tetrahydroxyflavone	$C_{15}H_{10}O_6$	13.644	** [M+H]+	287.0550	287.0548	−0.7	DP, KP
183	Luteolin 7-*O*-diglucuronide	$C_{27}H_{26}O_{18}$	14.412	[M−H]−	637.1046	637.1043	−0.5	Mu, KP
184	Apigenin 6-C-glucoside	$C_{21}H_{20}O_{10}$	15.444	** [M+H]+	433.1129	433.1129	0.0	QP, KP, Mu
185	Dalspinin	$C_{17}H_{12}O_7$	16.775	[M+H]+	329.0656	329.0651	−1.5	KP, QP

186	5,3',5'-Trihydroxy-3,6,7,8,4'-pentamethoxyflavone	$C_{20}H_{20}O_{10}$	17.098	[M+H]+	421.1129	421.1122	−1.7	Mu
187	Apigenin 6,8-C-arabinoside-C-glucoside	$C_{26}H_{28}O_{14}$	17.455	[M−H]−	563.1406	563.1401	−0.9	KP
188	Myricetin 3-glucuronide	$C_{21}H_{18}O_{14}$	18.929	[M−H]−	493.0624	493.0634	2.0	KP
189	5,7,3',4',5'-Pentahydroxy-3,6,8-trimethoxyflavone	$C_{18}H_{16}O_{10}$	19.617	[M−H]−	391.0671	391.0690	4.9	QP
190	Dalpalatin	$C_{18}H_{14}O_{8}$	20.050	** [M−H]−	357.0616	357.0624	2.2	Mu, KP
191	Pinnatifinoside A	$C_{21}H_{18}O_{9}$	21.192	[M+H]+	415.1024	415.1011	−3.1	KP
192	Cuneatin	$C_{17}H_{12}O_{6}$	21.192	[M+H]+	313.0707	313.0704	−1.0	KP, Mu
193	Apigenin 6,8-di-C-glucoside	$C_{27}H_{30}O_{15}$	22.465	** [M+H]+	595.1658	595.1653	−0.8	QP, Mu, KP
194	5,7-Dihydroxyflavone (chrysin)	$C_{15}H_{10}O_{4}$	22.800	[M+H]+	255.0652	255.0654	0.8	QP, Mu, KP
195	Pongamoside A	$C_{23}H_{20}O_{9}$	23.101	** [M−H]−	439.1034	439.1037	0.7	Mu
196	Tricin 7-*O*-glucuronide	$C_{23}H_{22}O_{13}$	23.411	** [M−H]−	505.0987	505.0980	−1.4	KP
197	Syringetin-3-*O*-glucoside	$C_{23}H_{24}O_{13}$	24.141	[M−H]−	507.1144	507.1133	−2.2	KP, Mu
198	Kaempferitrin	$C_{27}H_{30}O_{14}$	24.432	[M+H]+	579.1714	579.1729	2.7	Mu
199	Luteolin	$C_{15}H_{10}O_{6}$	25.012	** [M+H]+	287.0550	287.0551	0.3	Mu, KP, QP
200	6-Hydroxyluteolin	$C_{15}H_{10}O_{7}$	25.322	[M+H]+	303.0499	303.0498	−0.3	Mu, KP, QP, DP
201	Exoticin	$C_{23}H_{26}O_{10}$	25.371	** [M−H]−	461.1453	461.1455	0.4	QP, KP
202	5,6-Dihydroxy-7,8,3',4'-tetramethoxyflavone	$C_{19}H_{18}O_{8}$	26.449	** [M−H]−	373.0929	373.0930	0.3	KP
203	Apigenin 7-*O*-glucuronide	$C_{21}H_{18}O_{11}$	27.418	[M−H]−	445.0776	445.0781	1.1	KP
204	8-Methoxyluteolin	$C_{16}H_{12}O_{7}$	28.182	** [M+H]+	317.0656	317.0656	0.0	Mu, KP
205	Apigenin 6,8-C-arabinoside-C-glucoside	$C_{26}H_{28}O_{14}$	28.567	[M+H]+	565.1552	565.1540	−2.1	Mu
206	Neoraunone	$C_{19}H_{16}O_{5}$	28.873	** [M+H]+	325.1071	325.1072	0.3	KP, QP, Mu
207	Diosmin	$C_{28}H_{32}O_{15}$	29.709	[M+H]+	609.1814	609.1796	−3.0	Mu, KP
208	Apigenin 7-*O*-(6''-malonyl-apiosyl-glucoside)	$C_{29}H_{30}O_{17}$	30.019	** [M−H]−	649.1410	649.1416	0.9	KP
209	Luteolin 7-*O*-glucuronide	$C_{21}H_{18}O_{12}$	31.180	[M−H]−	461.0725	461.0717	−1.7	KP, DP
210	Chrysoeriol 7-*O*-glucoside	$C_{22}H_{22}O_{11}$	31.434	** [M+H]+	463.1235	463.1224	−2.4	Mu, KP, QP
211	Tricin	$C_{17}H_{14}O_{7}$	31.852	[M+H]+	331.0813	331.0805	−2.4	Mu
212	3,5-Dimethylquercetin glucoside	$C_{23}H_{24}O_{12}$	31.852	[M+H]+	493.1341	493.1341	0.0	Mu, KP
213	Tangeretin	$C_{20}H_{20}O_{7}$	32.900	** $[M+H]^+$	373.1282	373.1285	0.8	KP
214	Isoswertin 2''-rhamnoside	$C_{28}H_{32}O_{14}$	33.414	** $[M+H]^+$	593.1865	593.1854	−1.9	Mu
215	Tetramethylscutellarein	$C_{19}H_{18}O_{6}$	34.235	$[M+H]^+$	343.1176	343.1189	3.8	KP, DP
216	Diosmetin	$C_{16}H_{12}O_{6}$	34.319	[M+H]+	301.0712	301.0723	3.7	Mu
217	Syzalterin	$C_{17}H_{14}O_{5}$	34.345	[M+H]+	299.0914	299.0918	1.3	KP
218	Eucalyptin	$C_{19}H_{18}O_{5}$	36.380	** $[M-H]^-$	325.1081	325.1083	0.6	KP, Mu
219	Swertisin	$C_{22}H_{22}O_{10}$	37.023	** $[M-H]^-$	445.1140	445.1160	4.5	Mu, QP, KP
220	Tricin 7-neohesperidoside	$C_{29}H_{34}O_{16}$	37.217	[M−H]−	637.1774	637.1747	−4.2	KP
221	Velutin	$C_{17}H_{14}O_{6}$	37.332	[M+H]+	315.0863	315.0857	−1.9	Mu, QP, KP

222	2''-*O*-Acetylrutin	$C_{29}H_{32}O_{17}$	39.318	** [M−H]$^-$	651.1567	651.1523	−6.8	KP, DP
223	Irilone	$C_{16}H_{10}O_6$	39.487	[M+H]$^+$	299.0550	299.0548	−0.7	Mu, DP
224	Spinacetin 3-rutinoside	$C_{29}H_{34}O_{17}$	39.815	[M−H]−	653.1723	653.1728	0.8	KP
225	3'-Hydroxy-3,5,6,7,8,4',5'-heptamethoxyflavone	$C_{22}H_{24}O_{10}$	40.399	** [M−H]−	447.1297	447.1302	1.1	Mu, KP
226	3',4',7-Tri-*O*-methylisoorientin	$C_{24}H_{26}O_{11}$	44.326	[M−H]−	489.1402	489.1400	−0.4	Mu, KP
227	Chrysoeriol 7-*O*-(6''-malonyl-glucoside)	$C_{25}H_{24}O_{14}$	44.667	** [M−H]−	547.1093	547.1112	3.5	DP, KP
228	5-Methoxy-7,8-diprenylflavone	$C_{26}H_{28}O_3$	53.244	** [M+H]+	389.2111	389.2103	−2.1	KP, Mu
229	4'-Hydroxy-3,5,3'-trimethoxy-7-prenyloxyflavone	$C_{23}H_{24}O_7$	54.024	[M+H]+	413.1595	413.1589	−1.5	Mu
230	Artocarpetin B	$C_{22}H_{22}O_6$	54.105	[M+H]+	383.1489	383.1477	−3.1	Mu
231	Melinervin	$C_{18}H_{14}O_9$	54.997	** [M+H]+	375.0711	375.0699	−3.2	Mu, QP
232	Lonicerjaponin B	$C_{34}H_{44}O_{17}$	64.417	** [M−H]−	723.2505	723.2513	1.1	KP
	Flavanones							
233	8-Prenylnaringenin	$C_{20}H_{20}O_5$	4.354	** [M+H]+	341.1384	341.1394	2.9	DP, KP
234	Hesperetin 3'-sulfate	$C_{16}H_{14}O_9S$	11.178	** [M+H]+	383.0431	383.0450	5.0	KP, DP
235	Muscomin	$C_{18}H_{18}O_7$	16.653	[M+H]+	347.1125	347.1125	0.0	QP, KP, Mu
236	Neoeriocitrin	$C_{27}H_{32}O_{15}$	17.535	[M−H]−	595.1668	595.1685	2.9	KP
237	Kaempferol 7-(6''-galloylglucoside)	$C_{28}H_{24}O_{15}$	21.479	[M−H]−	599.1042	599.1047	0.8	Mu, KP
238	Narirutin	$C_{27}H_{32}O_{14}$	22.717	[M−H]−	579.1719	579.1791	12.4	KP
239	Naringin	$C_{27}H_{32}O_{14}$	23.083	[M−H]−	579.1719	579.1665	−9.3	DP
240	Narirutin 4'-*O*-glucoside	$C_{33}H_{42}O_{19}$	23.707	[M+H]+	743.2393	743.2357	−4.8	Mu, KP
241	Pinocembrin (dihydrochrysin)	$C_{15}H_{12}O_4$	27.905	[M+H]+	257.0809	257.0809	0.0	QP
242	Hesperetin 3'-*O*-glucuronide	$C_{22}H_{22}O_{12}$	28.182	** [M+H]+	479.1184	479.1173	−2.3	Mu, KP
243	Hesperetin 3',7-*O*-diglucuronide	$C_{28}H_{30}O_{18}$	28.431	** [M−H]−	653.1359	653.1385	4.0	KP
244	Hesperidin	$C_{28}H_{34}O_{15}$	29.831	[M−H]−	609.1825	609.1810	−2.5	Mu, KP
245	Naringenin 7-*O*-glucoside	$C_{21}H_{22}O_{10}$	29.892	** [M−H]−	433.1140	433.1157	3.9	QP, Mu, KP
246	Brosimacutin C	$C_{20}H_{22}O_5$	31.651	[M−H]−	341.1394	341.1406	3.5	KP
247	Hesperetin 5-glucoside	$C_{22}H_{24}O_{11}$	35.691	** [M−H]−	463.1246	463.1253	1.5	Mu, QP
248	6''-Acetylliquiritin	$C_{23}H_{24}O_{10}$	40.111	** [M−H]−	459.1297	459.1301	0.9	Mu, KP, QP
249	Tsugafolin	$C_{17}H_{16}O_5$	41.914	** [M+H]+	301.1071	301.1072	0.3	KP, QP, Mu
250	Didymin	$C_{28}H_{34}O_{14}$	42.072	** [M+H]+	595.2022	595.2030	1.3	KP
251	Naringin 6'-malonate	$C_{30}H_{34}O_{17}$	42.392	** [M−H]−	665.1723	665.1707	−2.4	KP
252	6-Geranylnaringenin	$C_{25}H_{28}O_5$	51.603	** [M−H]−	407.1864	407.1903	9.6	KP
	Chalcones and Dihydrochalcones							
253	Phloretin	$C_{15}H_{14}O_5$	14.614	** [M+H]+	275.0914	275.0913	−0.4	QP, KP
254	Phloretin 2'-*O*-xylosyl-glucoside	$C_{26}H_{32}O_{14}$	17.343	[M−H]−	567.1719	567.1739	3.5	QP, KP
255	2'-Hydroxyfurano[2'',3'':4',3']chalcone	$C_{17}H_{12}O_3$	26.162	[M+H]+	265.0859	265.0849	−3.8	KP
256	Phloretin 2'-*O*-glucuronide	$C_{21}H_{22}O_{11}$	26.334	** [M+H]+	451.1235	451.1235	0.0	KP
257	Dihydropedicin	$C_{18}H_{20}O_6$	27.747	[M+H]+	333.1333	333.1332	−0.3	KP

258	Myrigalone G	$C_{17}H_{18}O_4$	31.621	[M+H]+	287.1278	287.1267	−3.8	KP
259	3-Hydroxyphloretin 2'-*O*-xylosyl-glucoside	$C_{26}H_{32}O_{15}$	34.825	** [M−H]−	583.1668	583.1689	3.6	KP
260	Isoliquiritigenin	$C_{15}H_{12}O_4$	40.349	[M+H]+	257.0809	257.0819	3.9	QP
261	Ovalitenin A	$C_{18}H_{14}O_3$	44.848	[M+H]+	279.1016	279.1027	3.9	KP
262	Phloridzin	$C_{21}H_{24}O_{10}$	52.602	** [M+H]+	437.1443	437.1460	3.9	KP, Mu
263	2'-Hydroxy-4',6'-dimethoxychalcone	$C_{17}H_{16}O_4$	58.668	** [M+H]+	285.1122	285.1122	0.0	Mu, KP
264	2'-Hydroxy-6'-methoxy-4'-prenyloxydihydrochalcone	$C_{21}H_{24}O_4$	63.947	[M+H]+	341.1748	341.1750	0.6	QP, KP, Mu
	Isoflavonoids							
265	6''-*O*-Malonylgenistin	$C_{24}H_{22}O_{13}$	4.236	** [M−H]−	517.0987	517.0988	0.2	DP, KP
266	Tectorigenin 7-sulfate	$C_{16}H_{12}O_9S$	4.807	[M−H]−	379.0129	379.0140	2.9	DP
267	4'-*O*-Methylequol	$C_{16}H_{16}O_3$	5.322	** [M+H]+	257.1172	257.1176	1.6	KP, DP, KP
268	Puerarin	$C_{21}H_{20}O_9$	11.401	[M−H]−	415.1034	415.1019	−3.6	KP
269	2',7-Dihydroxy-4',5'-dimethoxyisoflavone	$C_{17}H_{14}O_6$	15.025	[M+H]+	315.0863	315.0850	−4.1	KP
270	Violanone	$C_{17}H_{16}O_6$	17.045	** [M+H]+	317.1020	317.1021	0.3	KP
271	12a-Hydroxypachyrrhizone	$C_{20}H_{14}O_8$	17.873	[M+H]+	383.0762	383.0749	−3.4	KP, QP, Mu
272	Dolineone	$C_{19}H_{12}O_6$	18.020	** [M−H]−	335.0561	335.0558	−0.9	QP, KP
273	Irisolidone 7-*O*-glucuronide	$C_{23}H_{22}O_{12}$	20.022	** [M+H]+	491.1184	491.1221	7.5	KP
274	3',4',5,7-Tetrahydroxyisoflavanone	$C_{15}H_{12}O_6$	21.372	[M+H]+	289.0707	289.0717	3.5	KP
275	Glycitin	$C_{22}H_{22}O_{10}$	27.063	** [M−H]−	445.1140	445.1155	3.4	KP, Mu, QP
276	3'-Hydroxy-*O*-desmethylangolensin	$C_{15}H_{14}O_5$	27.403	[M+H]+	275.0914	275.0906	−2.9	Mu, KP
277	6''-*O*-Acetylgenistin	$C_{23}H_{22}O_{11}$	27.506	[M−H]−	473.1089	473.1093	0.8	QP, KP
278	Puerarin 4'-*O*-glucoside	$C_{27}H_{30}O_{14}$	28.296	** [M+H]+	579.1709	579.1703	−1.0	Mu
279	Glycitein 7-*O*-glucuronide	$C_{22}H_{20}O_{11}$	28.470	** [M−H]−	459.0933	459.0933	0.0	KP
280	3',4',7-Trihydroxyisoflavanone	$C_{15}H_{12}O_5$	29.811	** [M+H]+	273.0758	273.0758	0.0	Mu, QP, KP, DP
281	Daidzein 7-*O*-glucuronide	$C_{21}H_{18}O_{10}$	30.066	** [M−H]−	429.0827	429.0828	0.2	KP, Mu
282	2'-Hydroxyformononetin	$C_{16}H_{12}O_5$	31.660	[M+H]+	285.0758	285.0753	−1.8	QP
283	3'-*O*-Methylviolanone	$C_{18}H_{18}O_6$	35.231	[M+H]+	331.1176	331.1170	−1.8	KP
284	Daidzin 4'-*O*-glucuronide	$C_{27}H_{28}O_{15}$	36.499	** [M−H]−	591.1355	591.1346	−1.5	KP, DP
285	6'-Hydroxyangolensin	$C_{16}H_{16}O_5$	37.579	[M−H]−	287.0925	287.0918	−2.4	Mu, KP
286	6''-*O*-Malonyldaidzin	$C_{24}H_{22}O_{12}$	37.633	[M−H]−	501.1038	501.1020	−3.6	KP
287	6''-*O*-Acetyldaidzin	$C_{23}H_{22}O_{10}$	44.531	[M−H]−	457.1140	457.1090	−10.9	KP
288	Equol 7-*O*-glucuronide	$C_{21}H_{22}O_9$	46.597	** [M−H]−	417.1191	417.1187	−1.0	KP
289	Dihydrobiochanin A	$C_{16}H_{14}O_5$	50.970	** [M+H]+	287.0914	287.0914	0.0	Mu
290	Dihydroformononetin	$C_{16}H_{14}O_4$	55.516	[M+H]+	271.0965	271.0962	−1.1	Mu, KP, DP
291	3'-Hydroxymelanettin	$C_{16}H_{12}O_6$	55.758	[M−H]−	299.0561	299.0590	9.7	MU, QP, KP
292	6''-*O*-Malonylglycitin	$C_{25}H_{24}O_{13}$	62.337	** [M+H]+	533.1290	533.1278	−2.3	DP, KP
	Flavans							
293	5,7-Dimethoxy-8-prenylflavan	$C_{22}H_{26}O_3$	4.777	[M−H]−	337.1809	337.1811	0.6	KP

294	Kanzonol L	$C_{30}H_{32}O_6$	6.260	[M+H]+	489.2272	489.2276	0.8	Mu, QP
295	Broussinol	$C_{20}H_{22}O_4$	51.632	[M+H]+	327.1591	327.1578	−4.0	KP, Mu
296	7-Hydroxyflavan	$C_{15}H_{14}O_2$	62.240	** [M+H]+	227.1067	227.1064	−1.3	KP, QP, Mu
	Other flavonoids							
297	Hoslundal	$C_{18}H_{14}O_5$	13.342	[M−H]−	309.0768	309.0762	−1.9	Mu
298	8-*p*-Hydroxybenzylquercetin	$C_{22}H_{16}O_8$	18.409	[M+H]+	409.0918	409.0937	4.6	Mu
299	Kaempferol 3-*O*-sophoroside 7-*O*-glucuronide	$C_{33}H_{38}O_{22}$	21.334	[M−H]−	785.1782	785.1811	3.7	Mu, KP
300	Dihydroisorhamnetin	$C_{16}H_{14}O_7$	39.049	[M−H]−	317.0667	317.0670	0.9	Mu, QP
301	Glabratephrin	$C_{24}H_{20}O_7$	43.347	** [M+H]+	421.1282	421.1280	−0.5	QP, KP
302	Ohobanin	$C_{18}H_{18}O_3$	47.789	** [M−H]−	281.1183	281.1179	−1.4	KP
303	Cyclocommunin	$C_{25}H_{24}O_6$	48.055	[M+H]+	421.1646	421.1648	0.5	KP
304	7,8,4'-Trimethylisoscutellarein	$C_{18}H_{16}O_6$	54.934	[M+H]+	329.1020	329.1020	0.0	Mu
	Tannins							
305	Granatin B	$C_{41}H_{28}O_{27}$	5.734	** [M−H]−	951.0745	951.0746	0.1	Mu
306	Potentillin	$C_{41}H_{28}O_{26}$	13.043	[M−H]−	935.0796	935.0797	0.1	KP, Mu
307	Procyanidin dimer B2	$C_{30}H_{26}O_{12}$	16.647	** [M−H]−	577.1351	577.1353	0.3	KP, Mu, QP
308	Pedunculagin	$C_{34}H_{24}O_{22}$	18.254	[M+H]+	785.0832	785.0809	−2.9	KP
309	Punicafolin	$C_{41}H_{30}O_{26}$	18.643	[M−H]−	937.0952	937.0947	−0.5	KP, Mu
310	Valolaginic acid	$C_{41}H_{30}O_{28}$	22.954	[M−H]−	969.0851	969.0874	2.4	KP
311	Prodelphinidin B4 3'-gallate	$C_{37}H_{30}O_{18}$	31.723	[M+H]+	763.1505	763.1486	−2.5	Mu
312	Procyanidin	$C_{30}H_{26}O_{13}$	37.038	[M+H]+	595.1446	595.1423	−3.9	KP, Mu
313	2,3-Dimethylellagic acid	$C_{16}H_{10}O_8$	40.916	** [M+H]+	331.0449	331.0450	0.3	KP, Mu
	Lignans							
314	Sesamin	$C_{20}H_{18}O_6$	4.054	** [M−H]−	353.1030	353.1042	3.4	KP
315	2-Hydroxyenterodiol	$C_{18}H_{22}O_5$	5.723	[M+H]+	319.1540	319.1503	−11.6	KP
316	Schisantherin A	$C_{30}H_{32}O_9$	12.444	** [M−H]−	535.1973	535.1958	−2.8	DP, KP
317	7-Hydroxymatairesinol	$C_{20}H_{22}O_7$	15.211	** [M−H]−	373.1293	373.1292	−0.3	KP
318	Trachelogenin	$C_{21}H_{24}O_7$	15.708	** [M+H]+	389.1595	389.1604	2.3	KP
319	Schisandrin C	$C_{22}H_{24}O_6$	18.721	[M−H]−	383.1500	383.1495	−1.3	KP, Mu
320	Enterodiol	$C_{18}H_{22}O_4$	23.410	[M−H]−	301.1445	301.1462	5.6	KP, DP
321	Conidendrin	$C_{20}H_{20}O_6$	27.437	** [M−H]−	355.1187	355.1193	1.7	KP, Mu
322	2-Hydroxyenterolactone	$C_{18}H_{18}O_5$	28.693	[M−H]−	313.1081	313.1088	2.2	KP
323	Secoisolariciresinol	$C_{20}H_{26}O_6$	28.847	[M−H]−	361.1656	361.1654	−0.6	KP
324	Todolactol A	$C_{20}H_{24}O_7$	29.005	** [M+H]+	377.1595	377.1570	−6.6	KP
325	Secoisolariciresinol-sesquilignan	$C_{30}H_{38}O_{10}$	29.112	[M−H]−	557.2392	557.2417	4.5	KP
326	Lariciresinol	$C_{20}H_{24}O_6$	30.648	[M+H]+	361.1646	361.1619	−7.5	KP
327	Enterolactone	$C_{18}H_{18}O_4$	31.174	** [M+H]+	299.1278	299.1259	−6.4	KP
328	Sesaminol	$C_{20}H_{18}O_7$	31.366	** [M−H]−	369.0979	369.1000	5.7	KP
329	Pinoresinol	$C_{20}H_{22}O_6$	31.579	** [M+H]+	359.1489	359.1473	−4.5	KP

330	Matairesinol	$C_{20}H_{22}O_6$	34.541	** [M+H]+	359.1489	359.1477	−3.3	KP, QP, Mu
331	Lariciresinol-sesquilignan	$C_{30}H_{36}O_{10}$	39.801	** [M+H]+	557.2381	557.2368	−2.3	KP
332	Justicidin B	$C_{21}H_{16}O_6$	40.423	[M+H]+	365.1025	365.1037	3.3	Mu
333	Schisandrin B	$C_{23}H_{28}O_6$	45.616	[M+H]+	401.1959	401.1962	0.7	KP
334	1-Acetoxypinoresinol	$C_{22}H_{24}O_8$	47.572	[M+H]+	417.1544	417.1555	2.6	KP
335	Schisandrin	$C_{24}H_{32}O_7$	58.148	[M+H]+	433.2221	433.2215	−1.4	DP, KP
336	Arctigenin	$C_{21}H_{24}O_6$	63.842	** [M−H]−	371.1500	371.1514	3.8	KP
337	Arctigenin-4-glucoside	$C_{27}H_{34}O_{11}$	64.532	[M+H]+	535.2179119	535.2186	1.3	Mu
338	Deoxyschisandrin	$C_{24}H_{32}O_6$	65.102	** [M−H]−	415.2126	415.2130	1.0	DP, KP
339	Schisanhenol	$C_{23}H_{30}O_6$	67.519	[M+H]+	403.2115	403.2106	−2.2	DP, KP
340	Diphyllin	$C_{21}H_{16}O_7$	68.433	[M+H]+	381.097402	381.0979	1.3	Mu
	Stilbenes							
341	trans-Resveratrol 3,5-disulfate	$C_{14}H_{12}O_9S_2$	5.200	[M+H]+	388.9996	389.0017	5.4	KP
342	d-Viniferin	$C_{28}H_{22}O_6$	14.935	[M−H]−	453.1343	453.1329	−3.1	KP
343	4-Hydroxy-3,5,4'-trimethoxystilbene	$C_{17}H_{18}O_4$	18.847	** [M−H]−	285.1132	285.1136	1.4	KP
344	Resveratrol 3-*O*-glucoside (Polydatin)	$C_{20}H_{22}O_8$	20.822	[M−H]−	389.1242	389.1236	−1.5	Mu, KP
345	Rhaponticin	$C_{21}H_{24}O_9$	21.317	** [M−H]−	419.1347	419.1332	−3.6	DP, KP
346	Resveratrol	$C_{14}H_{12}O_3$	25.797	[M−H]−	227.0713	227.0709	−1.8	Mu
347	trans-Resveratrol 3-*O*-glucuronide	$C_{20}H_{20}O_9$	29.097	** [M−H]−	403.1034	403.1040	1.5	KP
348	Pallidol	$C_{28}H_{22}O_6$	45.234	** [M+H]+	455.1489	455.1487	−0.4	KP
349	Dihydroresveratrol	$C_{14}H_{14}O_3$	52.207	[M−H]−	229.0870	229.0875	2.2	DP
	Other polyphenols							
	Alkylphenols							
350	4-Vinylphenol	C_8H_8O	33.550	** [M−H]−	119.0502	119.0504	1.7	KP, Mu
351	3-Ethylphenol	$C_8H_{10}O$	61.273	** $[M+H]^+$	123.0805	123.0805	0.0	Mu
	Coumarins and derivatives							
352	Esculetin	$C_9H_6O_4$	4.589	[M−H]−	177.0193	177.0210	9.6	KP
353	Urolithin C	$C_{13}H_8O_5$	4.930	[M−H]−	243.0299	243.0300	0.4	KP
354	Psoralen	$C_{11}H_6O_3$	5.928	[M−H]−	185.0244	185.0244	0.0	KP
355	6-Methylcoumarin	$C_{10}H_8O_2$	11.449	** $[M-H]^-$	159.0451	159.0445	−3.8	DP, KP
356	7-Methoxycoumarin	$C_{10}H_8O_3$	12.070	[M+H]+	177.0546	177.0544	−1.1	QP, KP, Mu
357	Scopoletin	$C_{10}H_8O_4$	15.996	[M−H]−	191.0350	191.0350	0.0	QP, KP, Mu, DP
358	Umbelliferone	$C_9H_6O_3$	16.840	[M+H]+	163.0390	163.0387	−1.8	KP, QP, DP
359	Mellein	$C_{10}H_{10}O_3$	22.175	** $[M+H]^+$	179.0703	179.0704	0.6	KP, Mu
360	Fraxetin	$C_{10}H_8O_5$	23.187	[M+H]+	209.044973	209.0441	−4.2	Mu
361	Urolithin A 3,8-*O*-diglucuronide	$C_{25}H_{24}O_{16}$	27.910	** $[M+H]^+$	581.1137	581.1140	0.5	DP, KP
362	5,7-Dimethoxycoumarin	$C_{11}H_{10}O_4$	33.498	** $[M+H]^+$	207.0652	207.0654	1.0	KP, Mu, QP
363	Coumarin	$C_9H_6O_2$	59.885	[M+H]+	147.0441	147.0441	0.0	Mu, QP, KP, DP
	Phenolic terpenes							
364	Rosmadial	$C_{20}H_{24}O_5$	28.356	[M+H]+	345.1697	345.1662	−10.1	KP

365	Carnosol	$C_{20}H_{26}O_4$	61.168	[M+H]+	331.1904	331.1925	6.3	KP
366	Carnosic acid	$C_{20}H_{28}O_4$	61.286	** $[M-H]^-$	331.1915	331.1937	6.6	KP, QP, Mu, DP
	Tyrosols and derivatives							
367	Oleuropein	$C_{25}H_{32}O_{13}$	6.228	** [M+H]+	541.1916	541.1912	−0.7	KP
368	Tyrosol 4-sulfate	$C_8H_{10}O_5S$	6.399	[M−H]−	217.0176	217.0188	5.5	KP
369	Ligstroside	$C_{25}H_{32}O_{12}$	17.239	** [M+H]+	525.1967	525.1974	1.3	KP
370	*p*-HPEA-EA	$C_{19}H_{22}O_7$	20.892	** [M−H]−	361.1293	361.1320	7.5	KP
371	Etrogol	$C_{13}H_{18}O_2$	28.296	[M+H]+	207.1380	207.1373	−3.4	Mu, KP
372	Tyrosol	$C_8H_{10}O_2$	31.579	[M+H]+	139.0754	139.0749	−3.6	KP
373	Oleoside 11-methylester	$C_{17}H_{24}O_{11}$	33.412	[M−H]−	403.1246	403.1246	0.0	QP, Mu, KP
374	Hydroxytyrosol	$C_8H_{10}O_3$	34.292	[M−H]−	153.0557	153.0562	3.3	Mu, KP
375	3,4-DHPEA-EDA	$C_{17}H_{20}O_6$	37.848	$[M-H]^-$	319.1187	319.1192	1.6	Mu, KP
	Xanthones							
376	Artonin P	$C_{25}H_{20}O_8$	7.566	** [M−H]−	447.1085	447.1084	−0.2	Mu
377	Beta-Mangostin	$C_{25}H_{28}O_6$	15.601	** [M−H]−	423.1813	423.1780	−7.8	DP
378	Mangiferin 6'-gallate	$C_{26}H_{22}O_{15}$	17.152	** [M−H]−	573.0886	573.0892	1.0	KP, DP
379	Mangiferin	$C_{19}H_{18}O_{11}$	19.274	** [M−H]−	421.0771	421.0783	2.8	KP, QP
380	Mangostinone	$C_{23}H_{24}O_5$	19.624	** $[M-H]^-$	379.1551	379.1557	1.6	KP
381	Trapezifolixanthone	$C_{23}H_{22}O_5$	21.047	[M−H]−	377.1394	377.1424	8.0	KP
382	Bellidifolin-8-*O*-glucoside	$C_{20}H_{20}O_{11}$	24.370	[M−H]−	435.0933	435.0933	0.0	Mu
383	Alpha-Mangostin	$C_{24}H_{26}O_6$	26.736	$[M+H]^+$	411.180738	411.1809	0.4	Mu
384	Garcinone B	$C_{23}H_{22}O_6$	54.024	** $[M+H]^+$	395.1489	395.1489	0.0	Mu, KP
	Other polyphenols							
386	Caffeoylspermidine	$C_7H_{19}N_3$	3.495	[M+H]+	146.1652	146.1644	−5.5	QP, Mu
387	Amaronol B	$C_{16}H_{14}O_8$	3.796	$[M-H]^-$	333.0616	333.0629	3.9	KP
388	Norathyriol	$C_{13}H_8O_6$	3.831	$[M-H]^-$	259.0248	259.0256	3.1	KP
389	Quinic Acid	$C_7H_{12}O_6$	4.076	$[M-H]^-$	191.0561	191.0564	1.6	Mu
390	Salvianolic acid D	$C_{11}H_{10}O_6$	4.217	$[M-H]^-$	237.0404	237.0382	−9.3	KP
391	2,3-Dimethylbenzofuran	$C_{10}H_{10}O$	4.387	$[M+H]^+$	147.0805	147.0802	−2.0	KP
392	2-Hydroxy-4-methoxyacetophenone 5-sulfate	$C_9H_{10}O_7S$	4.799	** $[M-H]^-$	261.0074	261.0081	2.7	KP, DP
393	Pyrogallol	$C_6H_6O_3$	6.257	$[M-H]^-$	125.0244	125.0235	−7.2	Mu, QP, KP
394	Demethoxycurcumin	$C_{20}H_{18}O_5$	8.851	** $[M-H]^-$	337.1081	337.1086	1.5	KP, Mu
395	Corylin	$C_{20}H_{16}O_4$	12.143	$[M-H]^-$	319.0976	319.0969	−2.2	QP, KP
396	Lithospermic acid	$C_{27}H_{22}O_{12}$	12.682	** $[M-H]^-$	537.1038	537.1029	−1.7	DP
397	4-Nitrophenol	$C_6H_5NO_3$	14.881	$[M+H]^+$	140.0342	140.0336	−4.3	Mu
398	Bisdemethoxycurcumin	$C_{19}H_{16}O_4$	14.966	$[M+H]^+$	309.1122	309.1125	1.0	KP
399	Juglone	$C_{10}H_6O_3$	15.422	$[M-H]^-$	173.0244	173.0257	7.5	QP, DP
400	Flemichapparin C	$C_{17}H_{10}O_6$	17.873	$[M+H]^+$	311.0550	311.0550	0.0	KP, QP
401	Salvianolic acid C	$C_{26}H_{20}O_{10}$	22.815	$[M-H]^-$	491.0983	491.1008	5.1	KP

402	Salvianolic acid G	$C_{20}H_{18}O_{10}$	30.969	** $[M+H]^+$	419.0973	419.0972	−0.2	KP, DP
403	p-Anisaldehyde	$C_8H_8O_2$	31.579	$[M+H]^+$	137.0597	137.0590	−5.1	KP
404	Estragole	$C_{10}H_{12}O$	34.495	$[M+H]^+$	149.0961	149.0959	−1.3	Mu
405	Ovalitenin B	$C_{19}H_{18}O_4$	44.848	$[M+H]^+$	311.1278	311.1270	−2.6	KP, Mu
406	Tephcalostan B	$C_{20}H_{12}O_6$	50.203	** $[M-H]^-$	347.0561	347.0558	−0.9	Mu, KP
407	Chavicol	$C_9H_{10}O$	55.619	$[M+H]^+$	135.0805	135.0803	−1.5	Mu, KP, QP
408	Feniculin	$C_{14}H_{18}O$	61.649	$[M+H]^+$	203.1431	203.1424	−3.4	KP, QP
409	Genipic acid	$C_9H_{12}O_4$	64.694	$[M+H]^+$	185.0809	185.0815	3.2	KP
410	Isopropylbenzene	C_9H_{12}	68.576	$[M+H]^+$	121.1012	121.1009	−2.5	Mu
	Terpenoids and sesquiterpenoids							
411	Bakkenolide B	$C_{21}H_{28}O_6S$	14.598	** $[M-H]^-$	407.1534	407.1526	−2.0	DP, KP
412	Isoobacunoic acid 17-β-D-glucoside	$C_{32}H_{44}O_{14}$	17.565	$[M+H]^+$	653.2804	653.2800	−0.6	QP
413	Citrusin	$C_{28}H_{34}O_{11}$	27.610	$[M-H]^-$	545.2028	545.2016	−2.2	Mu, QP
414	Esculentic acid	$C_{30}H_{48}O_5$	28.542	$[M+H]^+$	489.3580	489.3585	1.1	Mu
415	Limocitrin	$C_{17}H_{14}O_8$	29.826	[M+H]+	347.0767	347.0772	1.5	Mu
416	Albiflorin	$C_{23}H_{28}O_{11}$	31.642	$[M+H]^+$	481.1710	481.1721	2.4	Mu
417	Soyasaponin ag	$C_{54}H_{86}O_{22}$	53.635	$[M-H]^-$				Mu
418	Limonin	$C_{26}H_{30}O_8$	65.083	** $[M+H]^+$	471.2014	471.2004	−2.1	QP, KP
419	Corosolic acid	$C_{30}H_{48}O_4$	66.762	$[M-H]^-$	471.3480	471.3476	−0.8	Mu, KP
420	Perilloside B	$C_{16}H_{24}O_7$	67.749	$[M+H]^+$	329.1595	329.1588	−2.1	KP, Mu
421	Longifolene	$C_{15}H_{24}$	69.139	$[M+H]^+$	205.1951	205.1949	−1.0	Mu
	Alkaloids							
422	Caffeine	$C_8H_{10}N_4O_2$	15.909	$[M+H]^+$	195.0877	195.0859	−9.2	KP
423	Boldine	$C_{19}H_{21}NO_2$	17.219	$[M+H]^+$	326.1393	326.1402	2.9	Mu
424	Caffeoylcholine	$C_{14}H_{20}NO_4$	18.816	$[M+H]^+$	267.1470	267.1489	7.0	Mu
425	Glycocitridine	$C_{13}H_{13}NO_5$	19.423	$[M+H]^+$	264.0872	264.0884	4.6	Mu
426	Anabasamine	$C_{16}H_{19}N_3$	26.142	$[M+H]^+$	254.1657	254.1662	2.0	Mu
427	Convolidine	$C_{15}H_{19}NO_4$	35.926	$[M-H]^-$	276.1236	276.1225	−4.0	Mu

Mu = Muntries, KP= Kakadu plum, QP= Quandong peach, DP= Davidson plum ** = found in both modes; $[M+H]^+$ = positive mode of ionization, $[M-H]^-$ = negative mode of ionization.

Table S3. LC-MS semi-quantification of abundant phenolic metabolites (%)

No.	Compounds	Mu	QP	KP	DP
1	2,3-Dimethylellagic acid	0.00	0.00	4.64	2.10
2	3-*p*-Coumaroylquinic acid	1.28	1.61	0.00	0.00
3	3-Sinapoylquinic acid	0.00	1.32	0.00	0.00
4	Caffeic acid	2.38	0.00	1.25	2.60
5	Chlorogenic acid	1.19	2.49	0.00	0.00
6	Cinnamic acid	0.00	12.84	9.15	0.00
7	Ellagic acid	1.27	2.50	0.93	6.18
8	Ferulic acid	0.00	0.00	0.15	0.62
9	Gallic Acid	2.77	0.00	4.85	0.00
10	*p*-Coumaric acid	8.28	10.85	4.53	11.98
11	*p*-Hydroxybenzoic acid	0.00	4.94	0.00	1.28
12	Protocatechuic acid	10.30	8.94	6.83	0.00
13	Quinic acid	2.03	14.31	0.00	0.00
14	Sinapic acid	0.00	2.68	3.39	0.00
15	Syringic acid	0.90	0.00	5.49	0.00
	Total phenolic acids (%)	*36.39*	*62.46*	*41.22*	*24.76*
16	Cyanidin	1.01	1.32	0.00	1.97
17	Cyanidin 3-galactoside	1.84	1.36	0.00	0.00
18	Cyanidin 3-*O*-(6"-p-coumaroyl-glucoside)	0.00	0.00	0.00	2.32
19	Cyanidin 3-*O*-galactoside	0.00	0.00	0.00	5.28
20	Cyanidin 3-*O*-rutinoside	0.00	0.00	0.00	2.79
21	Cyanidin 3-rhamnoside	0.00	1.42	0.00	0.00
22	Cyanidin 3-rhamnoside 5-glucoside	0.83	1.27	0.00	0.00
23	Cyanidin 3-sambubioside	1.34	0.00	0.00	1.92
24	Delphinidin	1.89	2.82	0.00	0.00
25	Delphinidin 3-*O*-glucoside	1.94	3.86	0.00	4.16
26	Delphinidin 3-*O*-sambubioside	0.00	0.00	0.00	20.50
27	Petunidin 3-*O*-galactoside	1.24	0.00	0.00	1.09
28	Cyanidin 3-glucoside	0.00	1.25	0.00	3.06
29	Peonidin 3-sambubioside	0.00	0.72	0.00	5.68
30	Petunidin 3-sambubioside	0.00	0.00	0.00	5.23
	Total anthocyanins (%)	*10.10*	*14.02*	*0.00*	*54.00*
31	3'-*O*-Methylviolanone	0.00	0.00	4.55	0.00
32	Chrysin	1.18	0.03	1.23	0.00
33	Dalbergin	2.09	1.66	1.56	0.00
34	Diosmin	3.98	0.00	2.44	0.00
35	Epicatechin	10.73	6.22	4.62	0.00
36	Genistein	0.00	0.00	1.32	0.00

37	Isorhamnetin	2.63	0.00	8.59	0.00
38	Kaempferol	0.66	0.91	2.37	0.00
39	Luteolin	4.18	3.76	5.87	0.00
40	Myricetin	2.27	0.00	0.00	3.15
41	Naringin	0.00	0.00	0.00	3.63
42	Procyanidin B2	9.04	0.00	0.00	0.00
43	Phloretin	0.00	0.00	1.48	0.00
44	Quercetin	3.47	1.96	7.78	4.33
45	Quercetin-3-glucoside	0.00	0.00	1.89	0.00
46	Taxifolin	1.50	0.00	0.00	0.00
47	Rutin	0.90	0.80	1.45	2.99
48	Diosmetin	1.50	0.00	0.00	0.00
	Total flavonoids (%)	*44.13*	*15.34*	*45.13*	*14.11*
49	Coumarin	6.02	5.32	3.96	4.23
50	Pyrogallol	9.36	2.90	9.69	0.00
	Total other phenolics	*9.38*	*8.12*	*13.65*	*4.23*

Table S4. Virtual toxicological screening of most abundant phytochemical metabolites in native Australian fruits

No.	Compounds	MW	AMES toxicity	Max. tolerated dose (human)	hERG I inhibitor	hERG II inhibitor	Oral Rat Acute Toxicity (LD50)	Oral Rat Chronic Toxicity (LOAEL)	Hepatotoxicity	Skin Sensitization	T.Pyriformis toxicity pIGC50	Minnow toxicity LC50
1	3-*p*-Coumaroylquinic acid	338.312	No	-0.09	No	No	1.74	2.51	No	No	0.29	4.61
2	3-Sinapoylquinic acid	398.364	No	0.86	No	No	1.99	3.81	No	No	0.29	5.24
3	Caffeic acid	180.159	No	1.15	No	No	2.38	2.09	No	No	0.29	2.25
4	Chlorogenic acid	354.311	No	-0.13	No	No	1.97	2.98	No	No	0.29	5.74
5	Cinnamic acid	148.161	No	1.11	No	No	2.09	2.65	No	No	0.25	1.72
6	Ellagic acid	302.194	No	0.48	No	No	2.40	2.70	No	No	0.30	2.11
7	Ferulic acid	194.186	No	1.08	No	No	2.28	2.07	No	No	0.27	1.83
8	Gallic Acid	170.120	No	0.70	No	No	2.22	3.06	No	No	0.29	3.19
9	*p*-Coumaric acid	164.160	No	1.11	No	No	2.16	2.53	No	No	0.32	1.61
10	*p*-Hydroxybenzoic acid	138.122	No	0.85	No	No	2.26	2.48	No	No	0.27	1.81
11	Protocatechuic acid	154.121	No	0.81	No	No	2.42	2.02	No	No	0.27	2.45
12	Quinic acid	192.167	No	1.63	No	No	1.13	3.53	No	No	0.29	4.87
13	Sinapic acid	224.212	No	1.19	No	No	2.24	2.32	No	No	0.26	2.18
14	Syringic acid	198.174	No	1.37	No	No	2.16	2.42	No	No	0.28	2.55
15	Cyanidin	287.247	No	0.50	No	No	2.46	2.54	No	No	0.29	2.55
16	Cyanidin 3-galactoside	449.388	No	0.56	No	Yes	2.55	4.20	No	No	0.29	6.40
17	Cyanidin 3-*O*-(6"-p-coumaroyl-glucoside)	595.533	No	0.11	No	No	2.64	3.80	No	No	0.29	4.35
18	Cyanidin 3-*O*-galactoside	449.388	No	0.56	No	Yes	2.55	4.20	No	No	0.29	6.40
19	Cyanidin 3-*O*-rutinoside	595.530	No	0.46	No	Yes	2.50	3.29	No	No	0.29	5.70
20	Cyanidin 3-rhamnoside	433.389	No	0.50	No	No	2.57	2.56	No	No	0.29	4.12
21	Cyanidin 3-rhamnoside 5-glucoside	595.530	No	0.50	No	Yes	2.51	5.42	No	No	0.29	8.98
22	Cyanidin 3-sambubioside	581.503	No	0.47	No	Yes	2.50	4.08	No	No	0.29	5.60
23	Delphinidin	303.246	No	0.51	No	No	2.55	2.93	No	No	0.29	3.31
24	Delphinidin 3-*O*-glucoside	465.387	No	0.51	No	Yes	2.59	4.09	No	No	0.29	7.65
25	Delphinidin 3-*O*-sambubioside	597.502	No	0.45	No	Yes	2.49	4.26	No	No	0.29	6.94
26	Petunidin 3-*O*-galactoside	479.414	No	0.50	No	Yes	2.60	4.09	No	No	0.29	6.80
27	Cyanidin 3-glucoside	449.388	No	0.56	No	Yes	2.55	4.20	No	No	0.29	6.40
28	Peonidin 3-sambubioside	595.530	No	0.46	No	Yes	2.50	4.75	No	No	0.29	9.14
29	Petunidin 3-sambubioside	611.529	No	0.44	No	Yes	2.48	4.41	No	No	0.29	10.88
30	3'-*O*-Methylviolanone	611.529	No	0.44	No	Yes	2.48	4.41	No	No	0.29	10.88
31	Chrysin	254.241	No	0.02	No	No	2.29	0.96	No	No	0.54	1.75

32	Dalbergin	268.268	No	0.10	No	Yes	2.01	0.89	No	No	0.47	0.58
33	Diosmin	608.549	No	0.57	No	Yes	2.51	3.34	No	No	0.29	5.35
34	Epicatechin	290.271	No	0.44	No	No	2.43	2.50	No	No	0.35	3.59
35	Genistein	270.240	No	0.48	No	No	2.27	2.19	No	No	0.38	1.94
36	Isorhamnetin	316.265	No	0.58	No	No	2.41	2.50	No	No	0.30	2.21
37	Kaempferol	286.239	No	0.53	No	No	2.45	2.51	No	No	0.31	2.89
38	Luteolin	286.239	No	0.50	No	No	2.46	2.41	No	No	0.33	3.17
39	Myricetin	318.237	No	0.51	No	No	2.50	2.72	No	No	0.29	5.02
40	Naringin	580.539	No	0.43	No	Yes	2.50	4.20	No	No	0.29	6.04
41	Procyanidin B2	578.526	No	0.44	No	Yes	2.48	4.35	No	No	0.29	8.70
42	Phloretin	274.272	No	0.31	No	No	2.38	3.32	No	No	0.34	2.48
43	Quercetin	302.238	No	0.50	No	No	2.47	2.61	No	No	0.29	3.72
44	Quercetin-3-glucoside	464.379	No	0.57	No	Yes	2.54	4.42	No	No	0.29	8.06
45	Taxifolin	304.254	No	0.35	No	No	2.26	3.10	No	No	0.29	4.69
46	Rutin	610.521	No	0.45	No	Yes	2.49	3.67	No	No	0.29	7.68
47	Diosmetin	300.266	No	0.42	No	No	2.34	2.27	No	No	0.34	1.74
48	Coumarin	146.145	No	0.44	No	No	2.11	1.90	No	No	0.37	1.56
49	Pyrogallol	126.111	No	-0.27	No	No	2.05	2.37	No	No	0.13	2.73
50	3-Ethylphenol	122.167	No	0.54	No	No	2.20	2.04	No	Yes	0.03	1.48
51	4-Vinylphenol	120.151	No	0.55	No	No	2.22	2.10	No	Yes	0.05	1.48
52	Caffeoylcholine	266.317	No	-0.38	No	No	2.10	1.01	No	No	0.65	1.71
53	Caffeine	194.194	No	0.00	No	No	2.80	0.70	Yes	No	0.29	2.34
54	Boldine	327.380	No	0.21	No	Yes	2.51	0.71	No	No	0.73	1.44
55	Glycocitridine	263.249	No	0.76	No	No	2.06	1.83	No	No	0.74	2.05
56	Anabasamine	253.349	No	-0.42	No	No	3.01	1.76	Yes	No	0.38	1.48
57	Convolidine	277.320	No	-0.22	No	No	2.76	1.39	Yes	No	0.28	2.19
58	Longifolene	204.357	No	0.07	No	No	1.58	1.35	No	No	1.37	0.37
59	Corosolic acid	472.710	No	0.12	No	No	2.51	1.86	Yes	No	0.29	0.28
60	Esculentic acid	488.709	No	0.08	No	No	2.59	0.58	No	No	0.29	1.11
61	Estragole	148.205	Yes	1.06	No	No	1.90	1.98	No	Yes	0.42	1.40
62	Granatin B	952.648	No	0.44	No	Yes	2.48	9.02	No	No	0.29	18.28
63	Punicafolin	938.665	No	0.44	No	Yes	2.48	10.61	No	No	0.29	15.32
64	Valolaginic acid	970.663	No	0.44	No	No	2.48	10.38	No	No	0.29	24.50
65	Prodelphinidin B4 3'-gallate	762.629	No	0.44	No	Yes	2.48	7.84	No	No	0.29	7.51
66	Diphyllin	380.352	Yes	-0.34	No	Yes	2.31	0.47	No	No	0.29	0.78
67	Resveratrol	228.247	Yes	0.33	No	No	2.53	1.53	No	No	0.75	1.52
68	Corilagin	634.455	No	0.44	No	Yes	2.48	6.33	No	No	0.29	11.28
69	Punicalin	782.528	No	0.44	No	No	2.48	4.64	No	No	0.29	17.18
70	Kurigalin	636.471	No	0.44	No	Yes	2.49	7.00	No	No	0.29	4.42
71	Hoslundal	310.305	No	-0.09	No	No	2.03	1.06	No	No	0.53	0.05

72	Hesperidin	610.565	No	0.53	No	Yes	2.51	3.17	No	No	0.29	7.13
73	Neoeriocitrin	596.538	No	0.48	No	Yes	2.49	4.39	No	No	0.29	6.98
74	Tricin	330.292	No	0.35	No	No	2.23	1.82	No	No	0.33	1.75
75	Isorhamnetin	316.265	No	0.58	No	No	2.41	2.50	No	No	0.30	2.21
76	Quercetin	302.238	No	0.50	No	No	2.47	2.61	No	No	0.29	3.72
77	Dryopteric acid	348.307	No	0.49	No	No	2.73	3.48	No	No	0.29	4.23
78	Malvidin	331.300	No	0.55	No	No	2.35	2.41	No	No	0.33	1.22
79	Fraxetin	208.169	No	0.20	No	No	1.85	1.96	No	No	0.37	2.25
80	Coumarin	146.145	No	0.44	No	No	2.11	1.90	No	No	0.37	1.56
81	Umbelliferone	162.144	No	0.69	No	No	2.05	1.75	Yes	No	0.55	1.71
82	Mangiferin	422.342	No	0.58	No	No	2.40	4.28	No	No	0.29	5.90

MW = Molecular weight

Explanations

Minnow toxicity

LC50 values below 0.5Mm (log LC50 < −0.3) are regarded as high acute toxicity.

T.Pyriformis toxicity

pIGC50 (negative logarithm of the concentration required to inhibit 50% growth in log µg/L) is considered, with a value > −0.5 log µg/L is considered toxic.

AMES toxicity: if the AMES value is positive then the compound will be mutagenic.

Maximum tolerated dose: a value less than is equal to 0.477 log(mg/kg/day) is considered low and high if greater than 0.477 log(mg/kg/day).

Table S5. The estimated docking score (kcal/mol) and glide energy (kcal/mol) of phenolic metabolites in alanine aminotransferase (3IHJ)

No.	Entry Name	Energy	Glide rotatable bonds	Docking score	Glide ligand efficiency	Glide energy
1	Rutin	62.29	16.00	−10.59	−0.25	−64.34
2	Delphinidin 3-sambubioside	60.89	17.00	−9.13	−0.22	−46.14
3	Neoeriocitrin	76.54	15.00	−8.76	−0.21	−55.24
4	Cyanidin 3−rhamnoside 5−glucoside	83.02	16.00	−8.36	−0.20	−48.95
5	Delphinidin 3-glucoside	51.45	13.00	−8.23	−0.25	−50.04
6	Chlorogenic acid	34.27	11.00	−8.18	−0.33	−46.04
7	Corilagin	54.96	14.00	−7.96	−0.18	−59.21
8	(-)-Epicatechin gallate	14.98	11.00	−7.95	−0.25	−48.43
9	Cyanidin-3-glucoside	53.38	12.00	−7.91	−0.25	−46.31
10	Naringin	78.20	14.00	−7.90	−0.19	−48.32
11	3-Feruloylquinic acid	38.22	11.00	−7.90	−0.30	−46.14
12	Cyanidin 3-*O*-galactoside	57.67	12.00	−7.80	−0.24	−43.29
13	Punicafolin	44.15	24.00	−7.79	−0.12	−47.01
14	Cyanidin 3-sambubioside	76.86	16.00	−7.76	−0.19	−42.55
15	3-*p*-Coumaroylquinic acid	34.46	10.00	−7.75	−0.32	−40.53
16	Isoquercitrin	37.29	12.00	−7.62	−0.23	−44.62
17	Caftaric acid	13.44	12.00	−7.44	−0.34	−43.18
18	Diosmin	64.01	15.00	−7.32	−0.17	−34.53
19	Dihydrorobinetin	20.82	6.00	−6.88	−0.31	−31.20
20	Myricetin	19.16	7.00	−6.73	−0.29	−42.84
21	Procyanidin B2	35.98	13.00	−6.67	−0.16	−45.85
22	Taxifolin	25.75	6.00	−6.45	−0.29	−37.99
23	Apigenin 8-C-glucoside	39.72	10.00	−6.36	−0.21	−40.37
24	Petunidin 3-glucoside	53.67	13.00	−6.34	−0.19	−45.07
25	3-*O*-Sinapoylquinic acid	41.80	12.00	−6.33	−0.23	−41.68
26	Mangiferin	43.00	10.00	−6.14	−0.21	−36.14
27	Quercetin	20.01	6.00	−6.06	−0.28	−36.21
28	Dihydromyricetin	25.03	7.00	−5.90	−0.26	−37.01
29	Quercetin 3-*O*-glucuronide	41.37	12.00	−5.80	−0.17	−50.73
30	Quinic acid	32.61	6.00	−5.79	−0.45	−24.85
31	Pelargonidin	23.50	5.00	−5.75	−0.29	−44.16
32	Pelargonidin chloride	23.50	5.00	−5.75	−0.29	−44.16
33	(+)-Catechin	16.89	6.00	−5.73	−0.27	−34.22
34	Malvidin	29.74	7.00	−5.72	−0.24	−48.79
35	Malvidin chloride	29.74	7.00	−5.72	−0.24	−48.79
36	(+)-Gallocatechin	14.26	7.00	−5.70	−0.26	−38.33
37	Hesperetin	26.78	5.00	−5.59	−0.25	−37.02
38	Cyanidin	22.76	6.00	−5.57	−0.27	−34.00
39	Cyanidin chloride	22.76	6.00	−5.57	−0.27	−34.00
40	Kaempferol	20.88	5.00	−5.54	−0.26	−37.01
41	Petunidin chloride	25.08	7.00	−5.50	−0.24	−35.83
42	Ellagic acid	22.03	4.00	−5.44	−0.25	−32.25
43	Isorhamnetin	24.13	6.00	−5.42	−0.24	−30.35
44	Luteolin	16.66	5.00	−5.37	−0.26	−33.28
45	Peonidin	26.82	6.00	−5.30	−0.24	−40.32
46	Delphinidin	21.55	7.00	−5.17	−0.24	−40.75
47	Chrysin	16.32	3.00	−5.06	−0.27	−31.17
48	Diosmetin	20.13	5.00	−5.05	−0.23	−33.27
49	(-)-Epicatechin	15.92	6.00	−5.04	−0.24	−32.45
50	Kaempferol 3-*O*-glucoside	41.60	11.00	−5.03	−0.16	−39.36

51	Acacetin	20.25	4.00	−4.97	−0.24	−32.89
52	Dihydroquercetin	28.22	6.00	−4.95	−0.23	−34.97
53	Fraxetin	13.44	3.00	−4.89	−0.33	−24.13
54	Gallic acid	4.88	5.00	−4.87	−0.41	−20.51
55	Sinapic acid	16.27	6.00	−4.83	−0.30	−27.23
56	Naringenin	23.37	4.00	−4.77	−0.24	−34.66
57	Dihydroresveratrol	9.40	6.00	−4.55	−0.27	−47.88
58	Sakuranetin	26.76	4.00	−4.54	−0.22	−30.83
59	Genistein	19.37	4.00	−4.50	−0.23	−30.22
60	2,3-Di-*O*-methylellagic acid	34.48	4.00	−4.49	−0.19	−40.95
61	Phloretin	26.07	8.00	−4.40	−0.22	−36.95
62	Syringic acid	13.55	5.00	−4.28	−0.31	−25.14
63	Vanillic acid	10.20	4.00	−4.25	−0.35	−21.90
64	Protocatechuic acid	5.68	4.00	−4.13	−0.38	−22.35
65	Carnosol	51.16	3.00	−4.11	−0.17	−29.05
66	Salicylic acid	10.70	3.00	−3.98	−0.40	−19.98
67	3-*O*-Methylviolanone	38.41	5.00	−3.94	−0.16	−32.68
68	7,8-Dihydroxyflavone	10.56	3.00	−3.87	−0.20	−30.56
69	Rosmanol	44.95	4.00	−3.81	−0.15	−25.81
70	Scopoletin	14.08	2.00	−3.70	−0.26	−22.75
71	Ferulic acid	12.04	5.00	−3.68	−0.26	−24.04
72	Resveratrol	13.54	5.00	−3.58	−0.21	−29.41
73	Umbelliferone	10.10	1.00	−3.56	−0.30	−20.31
74	Caffeic acid	7.81	5.00	−3.56	−0.27	−24.02
75	Dalbergin	22.85	3.00	−3.49	−0.18	−29.26
76	*p*-Hydroxybenzoic acid	6.48	3.00	−3.46	−0.35	−19.45
77	Coumarin	10.50	0.00	−3.28	−0.30	−21.25
78	*p*-coumaric acid	8.48	4.00	−3.27	−0.27	−21.94
79	Benzoic acid	7.12	2.00	−3.21	−0.36	−17.46
80	Carnosic acid	40.70	5.00	−3.20	−0.13	−24.72
81	4-Aminobenzoic acid	8.30	2.00	−3.16	−0.32	−19.60
82	Caffeine	29.44	0.00	−3.10	−0.22	−22.14
83	Cinnamic acid	7.29	3.00	−3.09	−0.28	−22.08
84	3-Methylcoumarin	11.69	0.00	−2.94	−0.25	−18.96
85	Estragole	10.04	3.00	−2.85	−0.26	−19.41
86	Pyrogallol	3.96	3.00	−2.81	−0.31	−16.13
87	4-Nitrophenol	6.61	2.00	−2.77	−0.28	−15.06
88	4-Vinylphenol	5.97	2.00	−2.70	−0.30	−14.26
89	3-Ethylphenol	4.22	2.00	−2.69	−0.30	−11.11
90	Polydatin	44.59	11.00	−2.61	−0.09	−68.54
91	*m*-Toluic acid	7.02	2.00	−2.28	−0.23	−20.63
